# Supplementary material for: Small molecule in situ resin capture provides a compound first approach to natural product discovery
Source: Nat Commun. 2024 Jun 19;15:5230. doi: 10.1038/s41467-024-49367-x (PMC11187115; doi:10.1038/s41467-024-49367-x)
Supplement: Supplementary file 1 — Supplementary Information [file 41467_2024_49367_MOESM1_ESM.pdf]

Supplementary Information for:

Small Molecule in situ Resin Capture Provides a Compound First Approach to Natural Product Discovery.

Authors

Alexander Bogdanov [1], Mariam N. Salib [2], Alexander B. Chase [1,3], Heinz Hammerlindl [4], Mitchell N. Muskat [1], Stephanie Luedtke [5], Elany Barbosa da Silva [5], Anthony J. O'Donoghue [5], Lani F. Wu [4], Steven J. Altschuler [4], Tadeusz F. Molinski [2*], Paul R. Jensen [1*].

Affiliations

[1] Scripps Institution of Oceanography, University of California, San Diego, La Jolla, CA 92093, USA.

[2] Department of Chemistry and Biochemistry, University of California, San Diego, La Jolla, CA 92093, USA.

[3] Department of Earth Sciences, Southern Methodist University, Dallas, TX 75275, USA.

[4] Department of Pharmaceutical Chemistry, University of California, San Francisco, San Francisco, CA 94158, USA.

[5] Skaggs School of Pharmacy and Pharmaceutical Sciences, University of California, San Diego, La Jolla, CA 92093, USA.

* Corresponding authors.

E-mail: pjensen@ucsd.edu, tmolinski@ucsd.edu

This document includes:

Supplementary Notes

Supplementary Figures 1 to 61

Supplementary Tables 1 to 18

Supplementary References 1 to 8

Supplementary Notes

Detailed structure elucidation of cabrillostatin (**1**)

The ^{1}H NMR spectrum exhibited three doublet resonances attributable to three methyl groups (δ_H 0.90, *J* = 6.5 Hz; 0.96, *J* = 6.7; 1.20, *J* = 6.3 Hz) positioned on methine carbons. In addition, three downfield resonances (δH 5.00, m; 4.11, ddd and 4.08, dt) were attributable to CH protons bonded to oxygen or nitrogen. The complete structure elucidation of the compound was accomplished by 2D NMR experiments (^{1}H-^{1}H COSY, HSQC, HMBC, Supplementary Table 1). The ^{1}H-^{1}H COSY experiment established the two spin systems in the molecule covering major parts of the carbon skeleton (16 out of 18 carbons, Supplementary Figure 2). A spin system comprising two methyl doublets at 0.96 and 0.90 ppm, coupled to a CH at 1.64 ppm, was further coupled to geminal CH_2 signals at 1.45 and 1.35 ppm, in turn sequentially correlated with a CH-N at 4.08 ppm then a CH-O at 4.11 ppm. The latter signal was finally correlated with another CH_2; the geminal protons at 2.54 (dd *J* = 4.5, 17.0 Hz) and 2.36 (dd *J* = 7.7, 17.0) ppm. HMBC correlation of the latter CH_2 to a carbonyl group at δ_C 173.3 ppm established the β-hydroxy-γ-amino acid residue, statine. The statine residue accounted for eight carbons, two oxygens and one nitrogen. Ten additional carbons and one oxygen remained to be assigned for completion of the backbone of the molecule. The second spin system contained one methyl doublet at 1.20 ppm that was coupled to a CH-O signal at 5.00 ppm. This downfield resonance was coupled to a methylene CH-12a at 1.46, that in turn correlated with another CH_2 at 1.32 and 1.44 ppm. The spin system extended further into the 'methylene envelope' at ~1.35 ppm. The distal end of this spin system comprised CH_2 protons at 2.18 and 2.20 ppm, characteristic of a methylene α to a carbonyl. The latter signals sequentially correlated with vicinal methylene protons at 1.48 and 1.71 ppm and overlapped methylene signals at 1.35 ppm. The CH_2 signal at 2.18 ppm exhibited an HMBC correlation to an amide carbonyl at 175.7 ppm establishing the connection of the spin system to the statine residue and a 9-hydroxy-decanonyl substructure. Both carbonyls accounted for two degrees of unsaturation (two C=O

double bonds) therefore, the remaining unsaturation is a ring. Despite the absence of a long range HMBC correlation from the methine proton at 5.00 ppm to the carbonyl at 173.3 ppm (due to low sample amount), the downfield shift of the proton is supportive of an ester or lactone functionality. The MS and MS-MS data provided additional confirmation of the structure (Supplementary Figures 3-4). Elimination of H_2O (fragment *m/z* 310.2377, M–18 Da) can only be explained with the presence of an unfunctionalized alcohol, i.e., the β-hydroxy group of the statine residue. Based on this structural feature and the provenance of the compound, we named the natural product cabrillostatin (**1**). The relative stereochemistry of the statine residue was tentatively assigned based on the NMR shifts in comparison with published values for statine or isostatine containing natural products.[1-3] Experimental ^{1}H NMR shifts and the *J* values of the CH-3, CH-4, and CH_2-2 resonances are consistent with a *syn* (e. g. 3*S*, 4*S* as present in miraziridin A [1] and pepstatin [3]) orientation of the NH and OH groups. The configuration of the C-13 distal stereocenter remains unassigned.

<u>Detailed structure elucidation of cabrillospiral A (**2**)</u>

NMR experiments (^{1}H, ^{1}H-^{1}H COSY, HSQC, HMBC, NOESY, 600 MHz, 1.7 mm microcryoprobe, CD_3CN) were conducted with 50 μg of **2**. After pooling compound from two SMIRC extracts (combined ~100 μg of **2**) the NMR experiments (^{1}H, COSY, and HSQC spectra) were repeated in CD_3OD resulting in better peak shapes with resolved signals and *S/N*. An LR-HSQMBC (optimized for *J* = 8 Hz) experiment [4] revealed additional correlations not observed in CD_3CN. The ^{13}C NMR chemical shifts were indirectly detected with heteronuclear coupled experiments, HSQC, and HMBC. Extensive analysis of the NMR data recorded in both solvents led to the structure (Supplementary Tables 2-3).

HSQC (CD_3CN) of **2** revealed 14 methines, seven methylenes, two methyls, and five quaternary carbons. In addition, one formyl group was identified from a sharp proton resonance at 9.58 ppm. A *para*-substituted phenol was established from ^{1}H, HSQC, and HMBC data with two coupled

aromatic H_2 doublets at 7.01 and 6.72 ppm (J = 8.0 Hz) that showed $^3J_{CH}$ HMBC cross peaks to quaternary signal C-24 at δ_C 154.2 ppm (characteristic of a phenol) and to the *para* C-21 δ_C 133.7 indicating an aliphatic substituent at this position. Two extensive ^{1}H-^{1}H spin systems (*A* and *B*) were identified from COSY and HSQC NMR (Supplementary Figure 7A). The aromatic protons in the *meta* position to the phenol OH exhibited $^3J_{CH}$ HMBC correlations to a benzylic 20-CH_2, δ_C 37.3 and established a connection to the first spin system *A*. Diastereotopic CH_2 signals (H-20a, δ 2.89; H-20b, δ 2.25) were coupled to H-19 (δ 1.89, m) and further correlated to a methyl doublet (δ_H 0.80, J = 6.8 Hz) and to 18-CH-O (δ_H 3.46, δ_C 78.3). From here, the spin system extended to a CH-O (4.20 ppm) that was vicinally coupled to a CH_2 (H-16a, δ 2.02; H-16b, δ 1.88), which subsequently correlated to the terminal CH_2 (H-15a, δ 2.37; H-15b, δ 1.92) of the spin system *A*.

The spin system *B* comprised 10 carbons and extended from a diastereotopic CH_2 (H-4a 3.29 ppm, doublet J = 17.5 Hz; H-4b 3.10, dd J = 4.9, 17.5 Hz) to 12-CH-O. H-4b was vicinally coupled to a CH-O at H-5 (δ 4.44), which further correlated with CH-O (H-6, δ 4.48). This COSY cross-peak was more pronounced in CD_3OD, where both proton resonances were better resolved (4.43 and 4.55 ppm, respectively). The system continued over CH_2-7 to a CH-8 that was coupled to the second CH_3 doublet of the molecule (H_3-26, δ 1.04, J = 6.2 Hz) and to a deshielded CH (H-9, δ 4.13), which in turn correlated to a CH-O (H-10, δ 4.32). H-10 coupled with the shielded proton H-11b of a diastereotopic CH_2 (H-11a, δ 2.39; H-11b, δ 1.73) and finally H-11a was correlated with a CH-O (H-12, δ 4.11).

The *A* and *B* spin systems were connected with the long-range heteronuclear correlations (HMBC). Crosspeaks of H_2-15 and H-16a with a deshielded quaternary C-14 (δ_C 109.6) placed it vicinal to C-15 at the end of spin system *A*. A further three-bond HMBC correlation of H-15a to a deshielded tertiary C-13, δ 63.2) connecting it with the quaternary C-14. On the other side, H-13 exhibited weak HMBCs to C-14 and to C-12, and together with an HMBC from H-12 to C-13 and

from OH-12 to both C-12 and C-13 providing evidence for the vicinity of CH-12 and CH-13 despite a missing COSY correlation and finally connected the spin systems *A* and *B*. The chemical shift of C-14 is characteristic for the spiro-center carbon in a 5,6 spiroketal. The substructure was established by connecting tertiary carbons C-10 (δ_C 67.3, 6-ring B) and C-17 (δ_C 86.2, 5-ring C) over oxygen bridges with the spiro-center C-14. To fully elucidate the planar structure, four remaining carbons (aldehyde, methylene at δ_C 54.7, and two quaternary carbons), three degrees of unsaturation, and the positions of Cl and Br needed to be assigned. Both methylene protons H_2-4 exhibited strong HMBC to a quaternary carbon C-3 at 179.4 ppm and proton H-4b to another quaternary C-2 resonance at 116.2 ppm that almost overlapped with two aromatic carbons C-23/23′ (δ_C 116.1) in the *ortho* position of the phenol ring D making the interpretation of the NMR data difficult. The aldehyde proton showed HMBC cross peaks with the hydroxymethyl terminus (C-27 at 54.7 ppm and the quaternary C-2). The methylene protons appeared as a broad singlet at 4.24 ppm in CD_3CN and lacked clear 2D correlations in CD_3CN, except a very weak HMBC to C-3. However, in CD_3OD they appeared as an 'AB quartet' (δ H-27a 4.33, H-27b 4.30, J = 11.3 Hz) and exhibited clear HMBC correlations to quaternary C-2, C-3, and to the aldehyde C-1 with an unusual shift of 192.1 ppm indicative of an α-β unsaturated aldehyde thus locating the CH=O and the -CH_2OH groups at C-2. The tetrahydrofuran (ring A) via oxygen bridge from C-6 to C-3 explained the deshielded resonances of both carbons (δ_C 87.7 and 179.4 respectively) and accounted for the last remaining degree of unsaturation. The positions of the halogens were determined based on the NMR shifts of the respective carbons, with inclusion of Cl within the spirobicyclic ring, and the analysis of the MS/MS fragmentation. In the high-resolution MS/MS spectrum we observed two major fragments with characteristic isotopic patterns characteristic for the chlorine containing *m/z* 277.0991 (calcd for $C_{16}H_{18}ClO_2$, 277.0990, 0.36 ppm) and bromine containing *m/z* 287.0268 (calcd for $C_{12}H_{16}BrO_3$, *m/z* 287.0277, -3.13 ppm) parts of the compound (Supplementary Figure 6). No fragments containing both halogens (other than those resulting

from multiple losses of H_2O) were observed, likely indicating a considerable spatial distance between the locations of the two halogens within the molecule. Even though it is challenging to predict how such a complex molecule would fragment in the ESI-MS, the calculated formulas and the associated degree of unsaturation allowed us to propose the structures of the major MS/MS fragments. The Br was assigned to the CH-9 (δ_C 65.7) and the Cl to CH-13 (δ_C 63.2); thus, securing the entire planar structure of **2**.

Stereochemical assignments of cabrillospiral A (**2**)

The stereochemical elucidation of structure **2**, with 11 chiral centers distributed across four independent stereogenic substructures, is a major challenge. Interpretation of the NOESY data and *J*-*J* coupling constants provided key insights. The first stereogenic system consists of the vinylogous formate ester and associated tetrahydrofuran (ring A, Supplementary Figure 7A). NOE between the formyl proton H-1 and the methylene protons H_2-4 demonstrated the *E* configuration of the $\Delta^{2,3}$ double bond. Clear NOE correlations from the proton H-4a to both oxygenated methine protons H-5 and H-6 provided evidence of their same facial orientation and established the relative configurations in ring A given the energetically favorable equatorial orientation of the aliphatic chain. The analysis of *J*-*J* coupling constants and NOESY data provided some information on the relative configuration associated with the [5,6]-spiroketal moiety. The most energetically stable conformation of the spiroketal benefits from two anomeric effects. The small coupling constant of 3.2 Hz between H-12 and H-13 indicates a dihedral angle of nearly >60 degrees and thus axial orientations of the OH and Cl substituents. The axial H-11b exhibits a large vicinal coupling (J = 11 Hz) to H-10 supporting their di-axial disposition and the energetically favorable equatorial orientation of the bulky aliphatic substituent at C-10.

NMR analysis, especially scalar coupling analysis, resolved the partial stereochemistry of several structural elements in **2** that were not immediately obvious from the NOE data. Instead, we clarified our understanding of the relative conformations within each sub-structure by analysis of vicinal coupling constants (^3J) in ^{1}H NMR and further refined by DFT calculations of the simplified model compounds **4a-4d** (Supplementary Figure 8). The *cis*-1,2-disubstituted furanylidene group (ring A, vinylogous formate ester) was suggested by observation of a ^3J ~ 3.5 Hz vicinal coupling constant of H-5 to H-6 and supported by DFT calculations of both *cis* and *trans* isomers of model compound **4** (Supplementary Figure 9) and Monte-Carlo searching of optimized geometries in the lowest-energy conformers (>90% of Boltzmann population). Only the *cis*-isomer, with an H-5–H-6 dihedral angle (q ~44.8˚), satisfies the Karplus relationship requirement (electronegative substituents are considered) for the $^3J_{H5\text{-}H6}$ ~3.5 Hz coupling constant.[5] NOE correlations from both H-5 and H-6 to H-4a further support the *cis* orientation, even though no NOE between H-5 and H-6 were observed due to signal overlap. The electronic circular dichroism spectrum of **2** showed a negative Cotton effect at λ 260 nm. Considering the free rotation of the phenyl D ring, the only remaining chromophore that could cause the observed Cotton effect is the α,β unsaturated aldehyde associated with the A ring. We performed DFT calculations with energy minimized model compounds 4a-4d and compared the Boltzmann weighted combined ECD spectra (Supplementary Figure 10) with the experimental ECD spectrum of cabrillospiral A (Supplementary Figure 7C) and determined the absolute configuration at the tetrahydrofuran ring to be 5*S*, 6*S*. To our surprise, the major contributor to the Cotton effect was the configuration at C-5, the bulky substituent at C-6 had no significant influence.

Investigation of the spiroketal ring B-C system of **2** led to a surprising interpretation: the C-12, C-13, and C-14 (spiro-C-O) substituents must each be axial with successively alternating α,β-orientations. This counter-intuitive conclusion assumes that the thermodynamically equilibrated spiro-system, like those of almost all known [6,5]- and [6,6]-spiroketal polyketides, is stabilized by

the anomeric effect: the C-O bond external to the pyran ring is invariably axial [6,7]. To test this 'triaxial hypothesis', model compound **5** (Supplementary Figure 8) was minimized (DFT) in a manner similar to that described above. The three lowest-energy conformers (>99% Boltzmann populations, Supplementary Figure 11) each benefit energetically from the expected anomeric effect, but they also exhibit C-12–C-14 triaxial disposition of substituents in each case. We rationalize this result by an understanding that the three polar substituents OH, Cl, and C-O at C-12–C-14 prefer conformations that minimize the net dipole of **5**, and that **5** is anchored by the anomeric effect at C-14. Unlike in some sugars, where intramolecular H-bonding plays a large role, this strong axial tendency is not compensated by vicinal hydrogen bond donor-acceptor effects (Cl is a weaker H-bond acceptor). So tenacious is this triaxial effect in [6,5]-spiroketal **5**, that the next highest energy conformer (~0.2% of Boltzmann population) is the twist-boat (Supplementary Figure 11), not the tri-equatorial chair conformer. In **2**, C-12–C-14 triaxial rigidity would be reinforced by a preference of the bulky branched substituent at C-10 to lay equatorial.

Two stereo-substructures remain to be assigned: the relative configurations at C-8–C-9 and C-17–C-19, but all stereoelements must finally be united by defining the inter-stereoelement relationships to secure the complete stereostructure of **2**. This challenging task, requiring a multipronged approach that includes a combination of ^{1}H and ^{13}C NMR chemical shift calculation guided by synthesis of suitable models, is underway.

<u>Cabrillospiral B (**3**)</u>

Cabrillospiral A (**2**) eluted with two minor compounds (Supplementary Figure 7B) that shared the same chromophore and were indistinguishable from **2** by MS/MS. After repeated rounds of isolation, we obtained one of the analogs in sufficient amount (27 μg) to record ^{1}H NMR, HSQC, and COSY experiments in CD_3OD. We assigned the structure of this minor isomer, cabrillospiral B (**3**), using the following reasoning: The few differences in the NMR spectra to that of **2** were found around ring A (Supplementary Table 4) and the most significant were the CH-O-5 (δ_C 73.6;

δ_H 4.28, dd (2.9, 5.9 Hz) vs. δ_C 70.5; δ_H 4.43 dd (3.5, 4.9)), the CH-O-6 (δ_C 90.9; δ_H 4.55 m vs. δ_C 88.5; δ_H 4.55 m), the CH_2-7 (δ_C 38.9; δ_H a 1.55 m, b 1.76 m vs. δ_C 34.9; δ_H a 1.85 m, b 1.92 m), and minor differences observed at CH-8, CH_3-26, and CBr-H-9. CH_2-4 resonances were obscured by overlap with the broad residual CHD_2OH solvent signal (low sample amount) and because of a possible deuterium exchange. Finally, the hydroxymethyl H_2-27 appeared as a broad singlet instead of an AB quartet in **2** while the chemical shifts remained unchanged. The electronic circular dichroism (ECD) spectra of **2** and **3** showed opposite Cotton effects at λ 260 nm (Supplementary Figure 7C) assigned to the vinylogous ester (ring A). Taken together, the NMR data (changes in chemical shifts in A-ring and at CH_2-7) and ECD evidence prompted us to propose **3** as diastereomer of **2**; specifically, the C-5 epimer (Supplementary Figure 7C).

Spectral data for cabrillostatin (**1**): for ^{1}H and ^{13}C NMR see Supplementary Table 1; UV/Vis: λmax 210 nm; HRMS (*m/z*): $[M+H]^+$ calcd. for $C_{18}H_{33}NO_4$, 328.2483, found 328.2485 (0.61 ppm); analysis (calcd., found for $C_{18}H_{33}NO_4$): C (65.82, 65.82), H (10.43, 10.43), Nr (4.26, 4.26), O (19.48, 19.48).

Spectral data for cabrillospirals A and B (**2-3**): for ^{1}H and ^{13}C NMR see Supplementary Tables 2-4; UV/Vis: λmax 268 nm; HRMS (*m/z*): $[M-H_2O+H]^+$ calcd. for $C_{29}H_{38}BrClO_8$, 629.1512, found 629.1510 (0.32 ppm); analysis (calcd., found for $C_{29}H_{38}BrClO_8$): C (55.2, 55.2), H (6.23, 6.23), Br (12.66, 12.66), Cl (5.62, 5.62) O (20.29, 20.29).

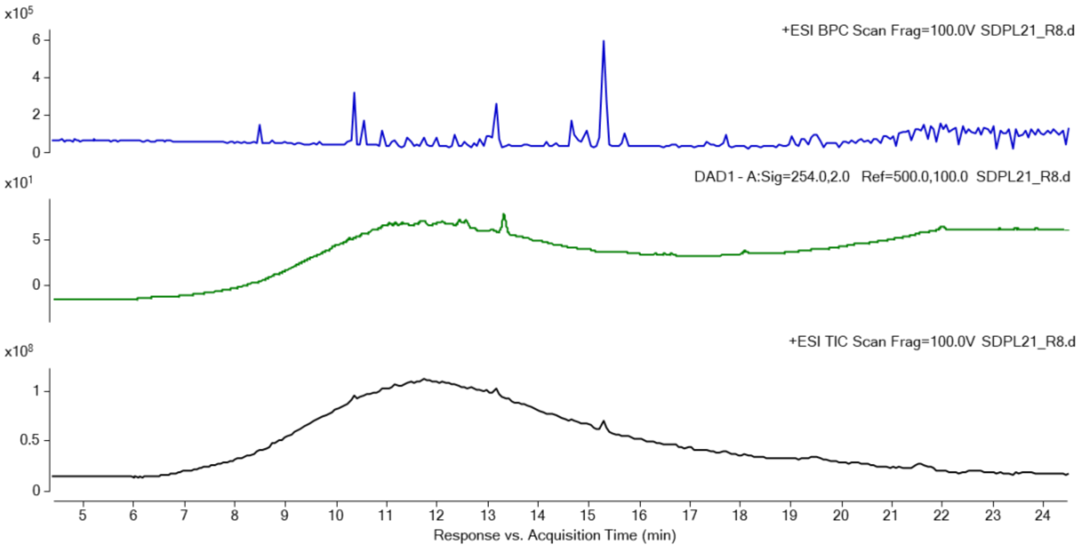

Supplementary Figure 1. LCMS analysis of a crude SMIRC extract from the CSMR deployment site. Upper chromatogram: base peak chromatogram (BPC), middle: diode array detection (DAD) UV 254 nm, lower: total ion count (TIC).

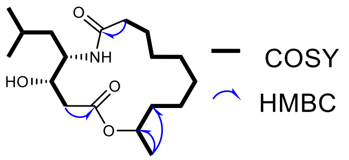

Supplementary Figure 2. Structure elucidation of cabrillostatin (1). Bold bonds indicate ^{1}H-^{1}H COSY correlations, blue arrows indicate key HMBC correlations.

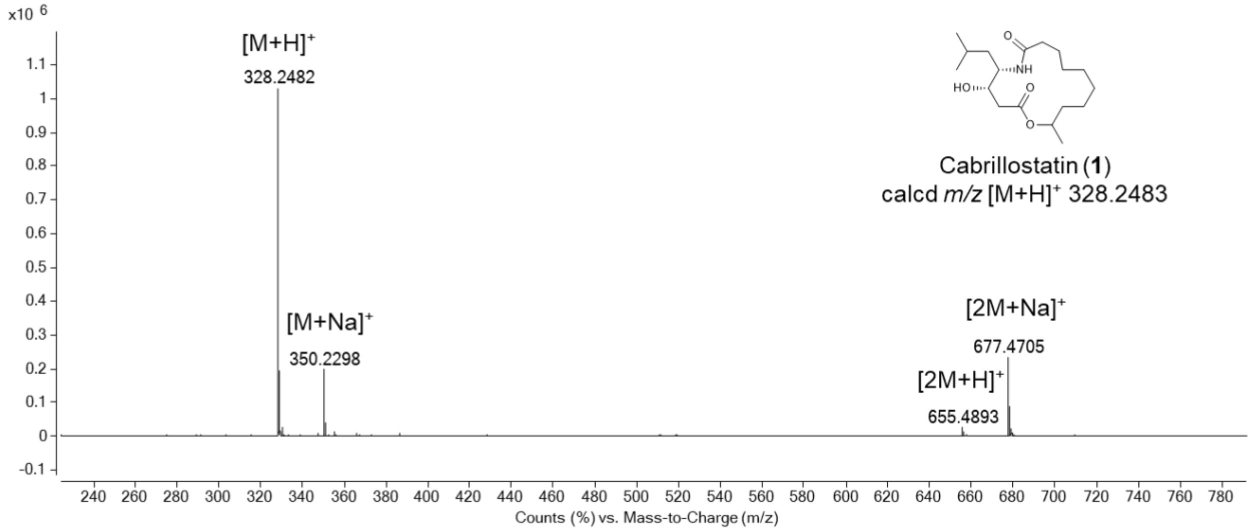

Supplementary Figure 3. HR-ESIMS of cabrillostatin (1).

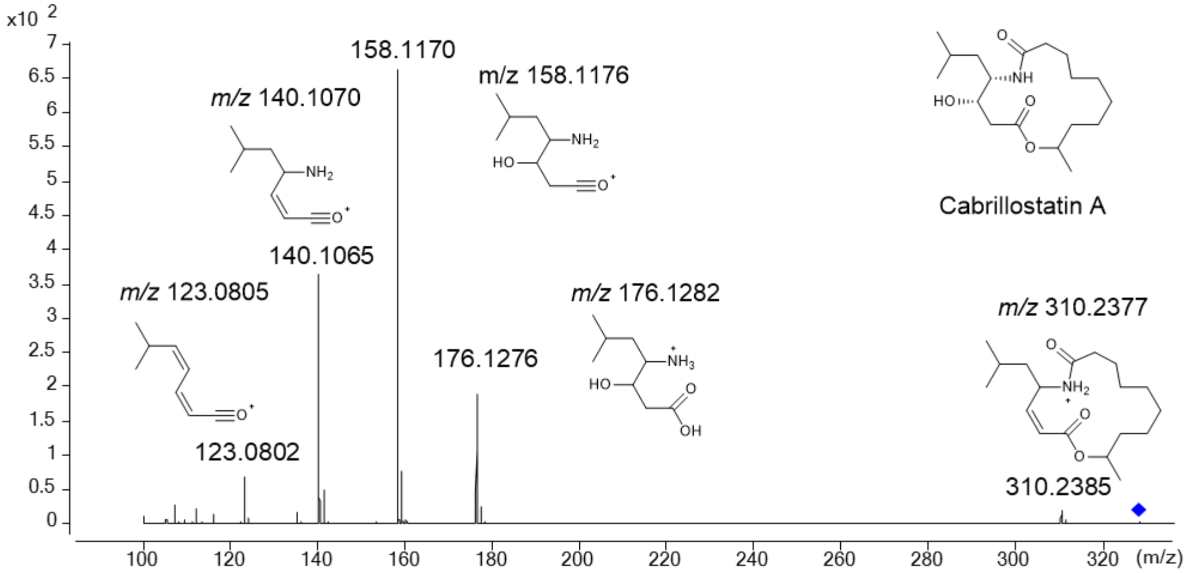

Supplementary Figure 4. **HR-ESIMS/MS of cabrillostatin (1).** Fragments annotated based on predicted fragmentation pattern.

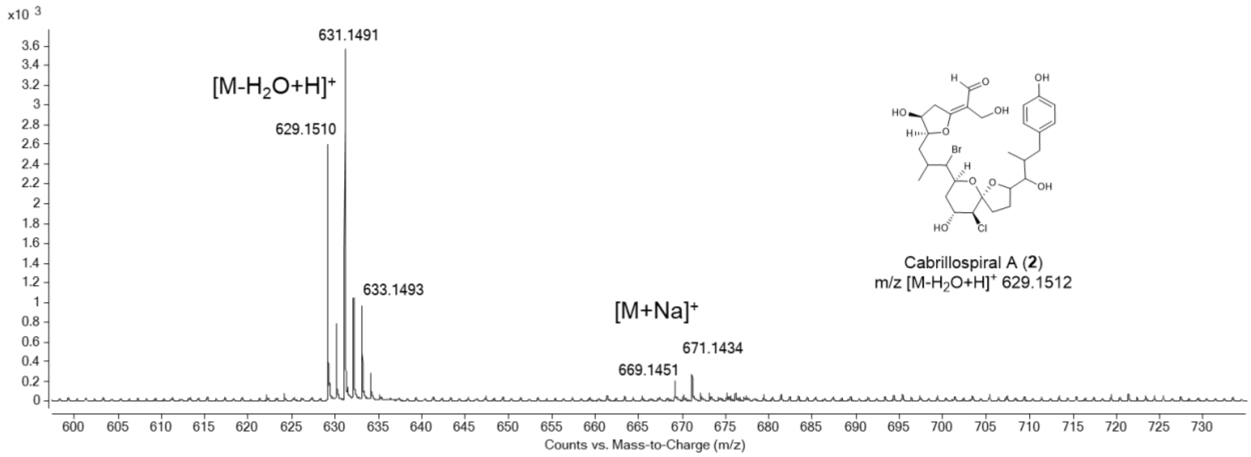

Supplementary Figure 5. HR-ESIMS spectrum and structure of cabrillospiral A (2).

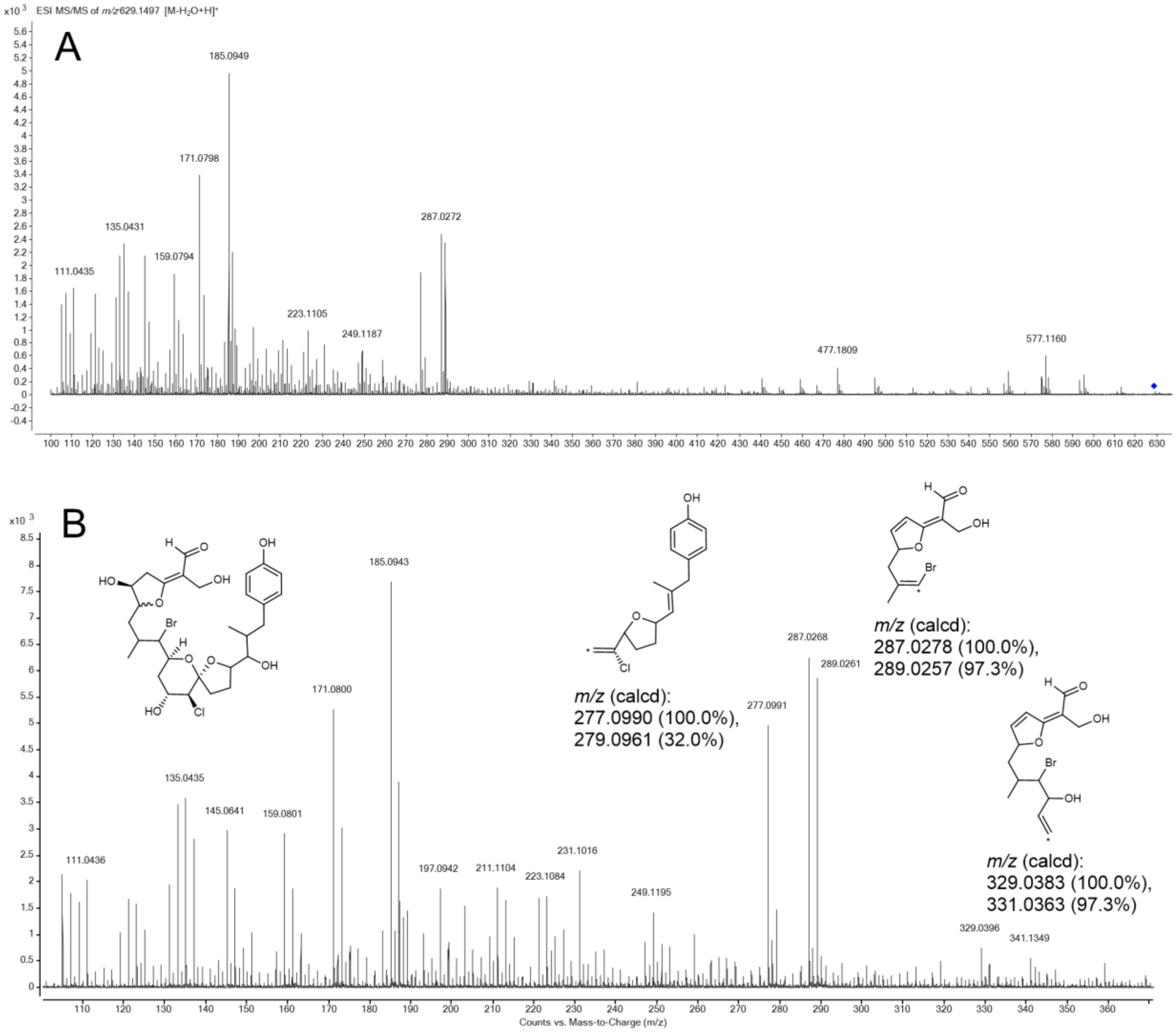

Supplementary Figure 6. HR-ESIMS/MS analysis of cabrillospiral A (2). (**A**) HR-ESIMS/MS spectrum of the [M-H2O+H]$^+$ precursor ion of **2**. (**B**) Expanded region with proposed halogen containing fragments.

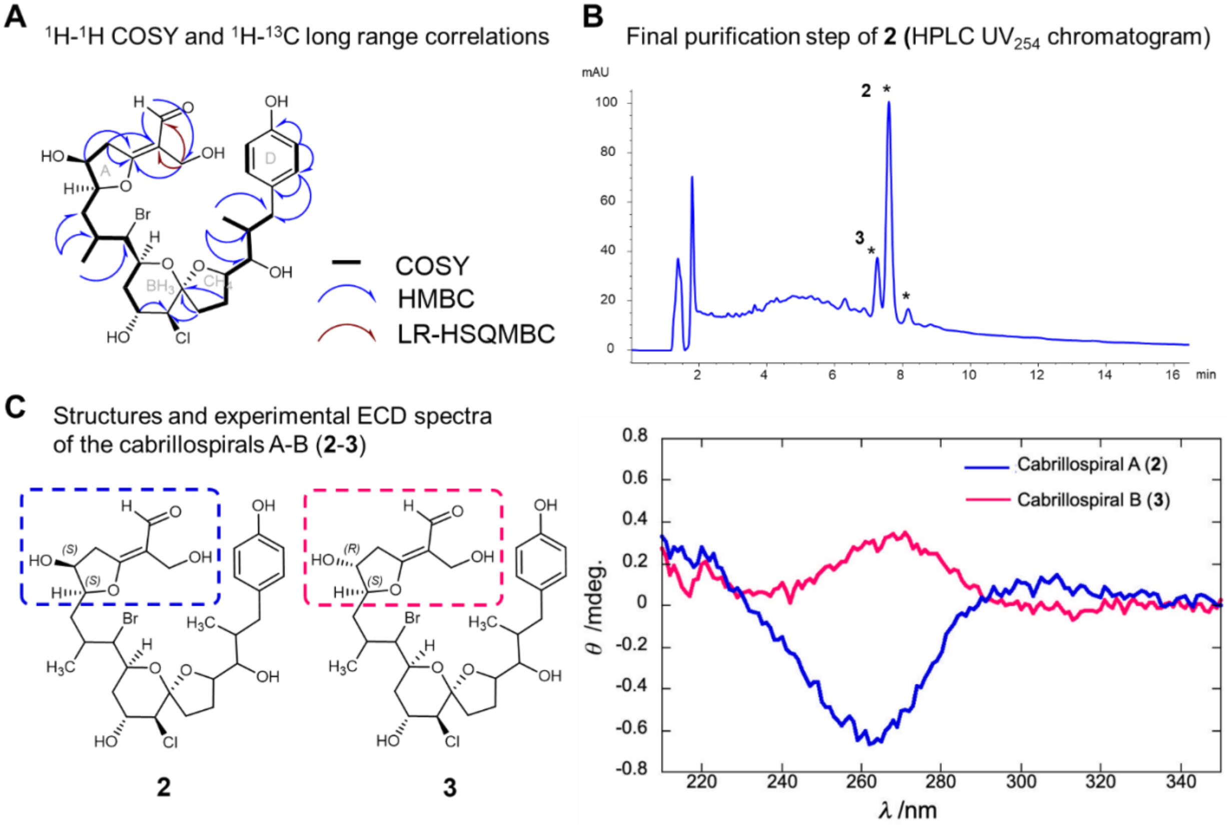

Supplementary Figure 7. Additional data for cabrillospirals A (2) and B (3). (**A**) HMBC and COSY correlations. (**B**) HPLC (high performance liquid chromatography) chromatogram from final purification step. Asterisks indicate peaks collected. (**C**). Experimental ECD (electronic circular dichroism) spectra of **2** and **3** and proposed structures of stereoisomeric **2** and **3.** Boxed segments highlight regions of the structures that exhibit the highest deviations in ^{1}H and ^{13}C NMR chemical shifts ($\Delta\delta$).

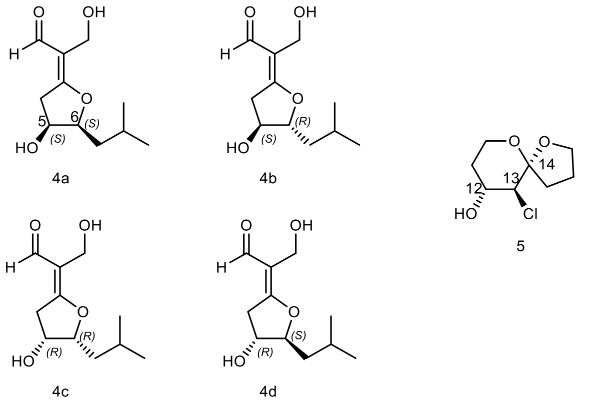

Supplementary Figure 8. Model compounds 4a-d and 5 for stereo-determination (^3J and DFT calculations) of ring A and spiroketal rings B and C in cabrillospiral A (2), respectively. DFT (density functional theory).

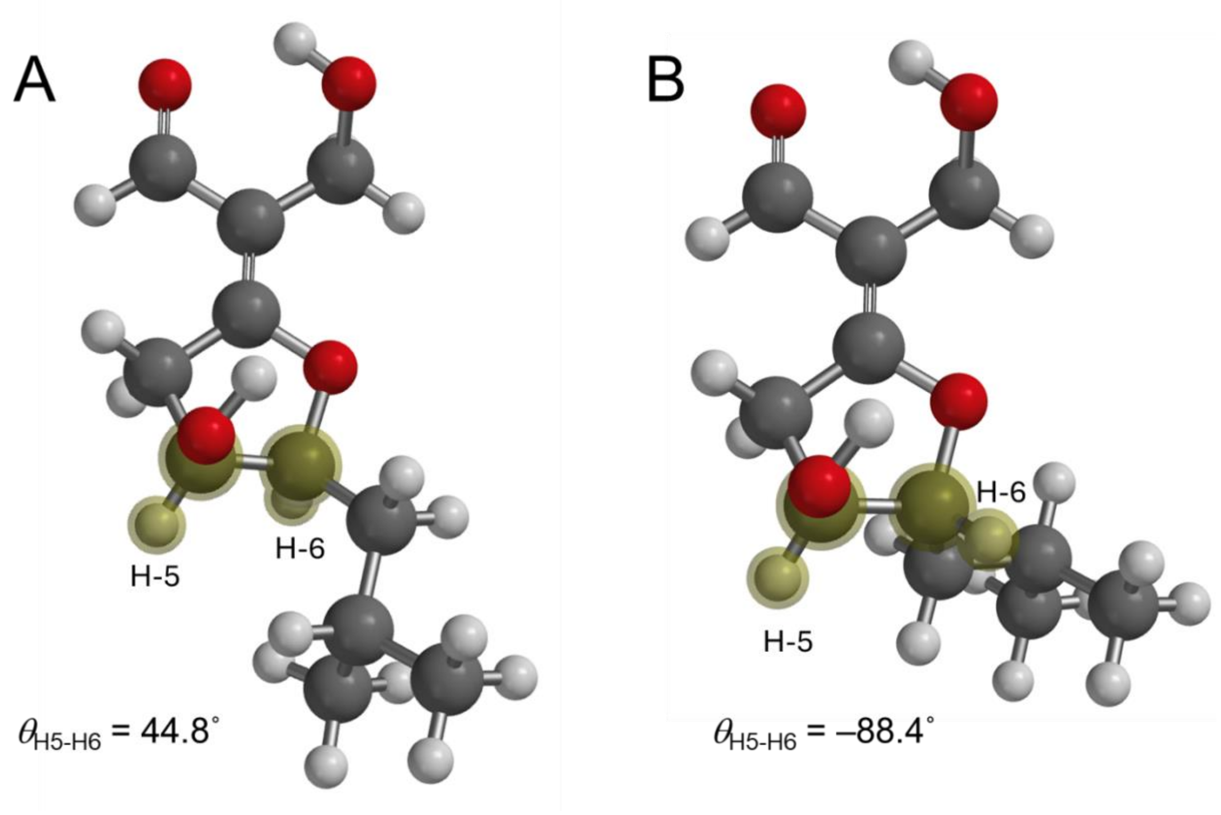

Supplementary Figure 9. DFT energy-minimized geometries of the lowest energy conformers (ωB97X-D, 6-31G*) for A (5*S*,6*S*)-*cis*-4 and B (5*S*,6*R*)-*trans*-4b. DFT (density functional theory).

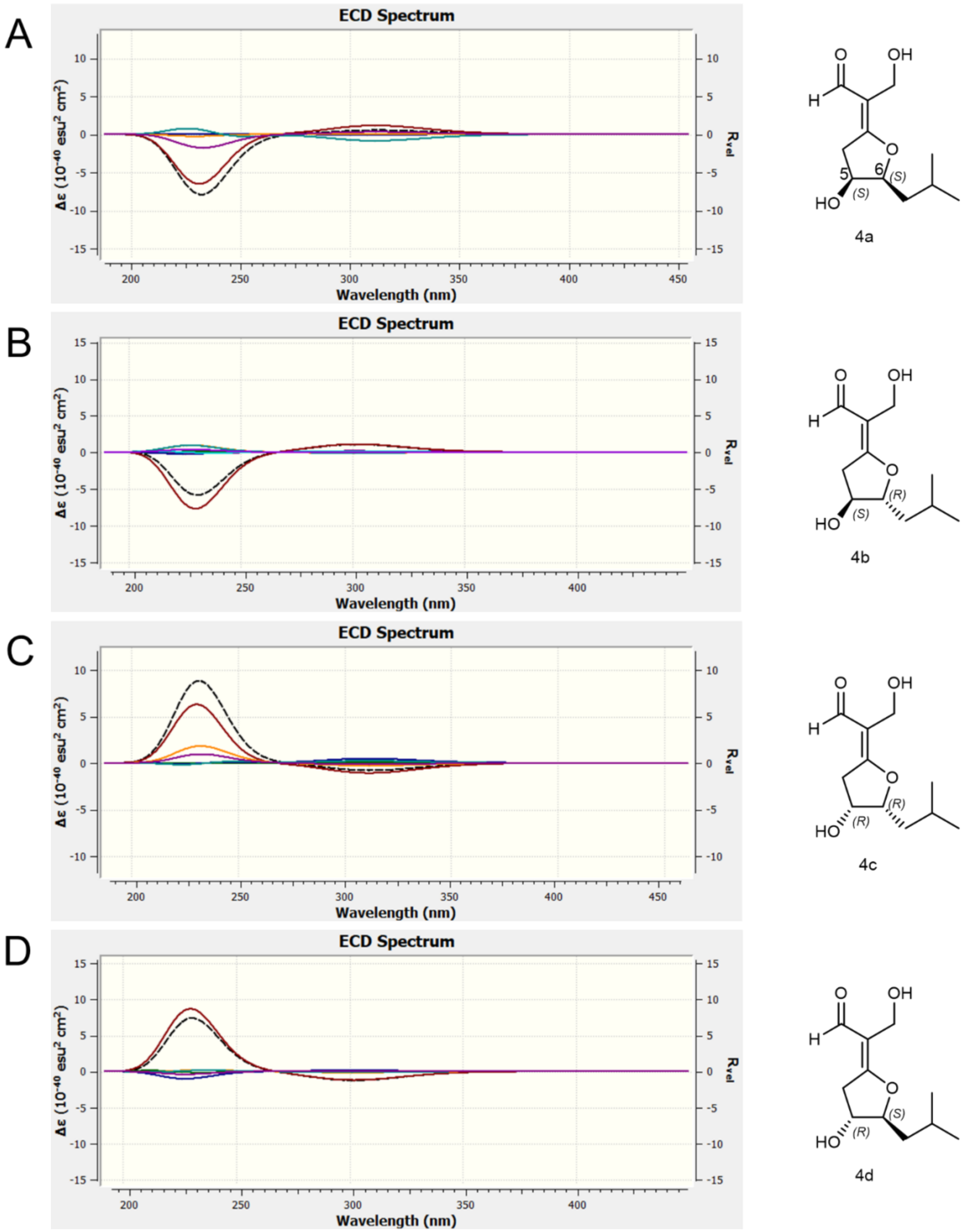

Supplementary Figure 10. (A-D) DFT calculated (ωB97X-D, 6-31G*) ECD spectra of model compounds 4a-4d. Combined and Boltzmann distribution weighted spectra are displayed as dashed black curves. Colored curves represent individual weighted spectra of the six lowest

energy conformers for each model compound. DFT (density functional theory), ECD (electronic circular dichroism).

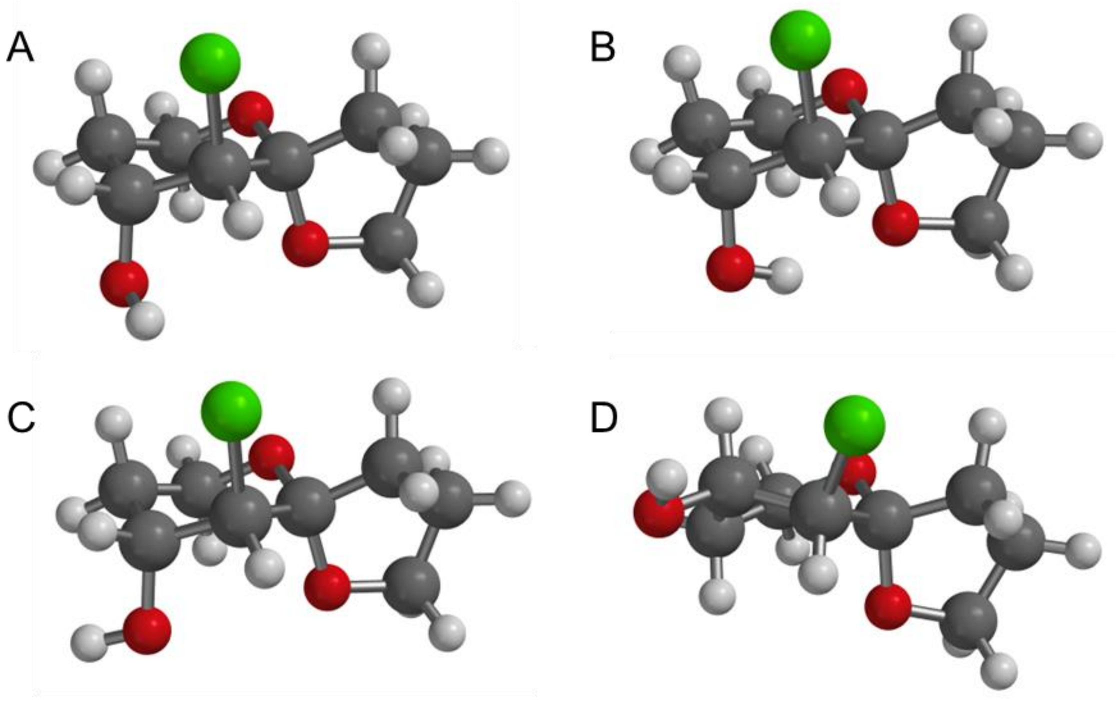

Supplementary Figure 11. Optimized geometries, minimized energies (DFT), and Boltzmann populations (%) of the four lowest energy structures of model compound 5. (**A**) **5a** E = 0 kcal.mol^{-1}, (99.2%). (**B**) **5b** 3.36 (0.2). (**C**) **5c** 3.57 (0.2). (**D**) **5d** 3.75 (0.2). DFT (density functional theory).

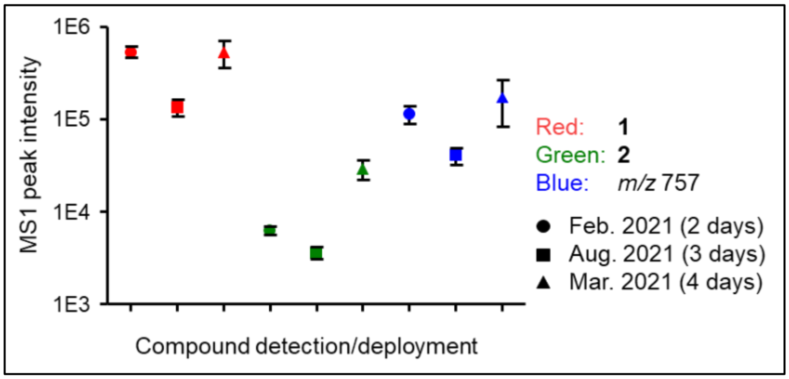

Supplementary Figure 12. **Reproducibility of three target compounds at the CSMR site.** In all cases, the relative abundance of parent ions corresponding to three compounds were highest for the longest deployments. Cabrillostatin (**1**), cabrillospiral A (**2**), unknown compound (*m/z* 757). Mean =/- standard deviation shown. Two days (n=4), three days (n=4), four days (n=3).

A

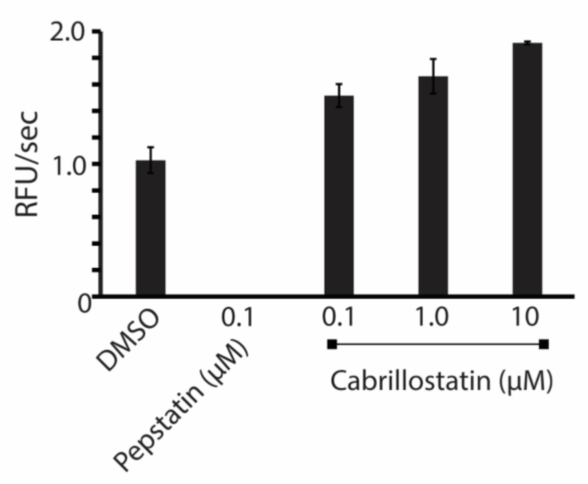

B

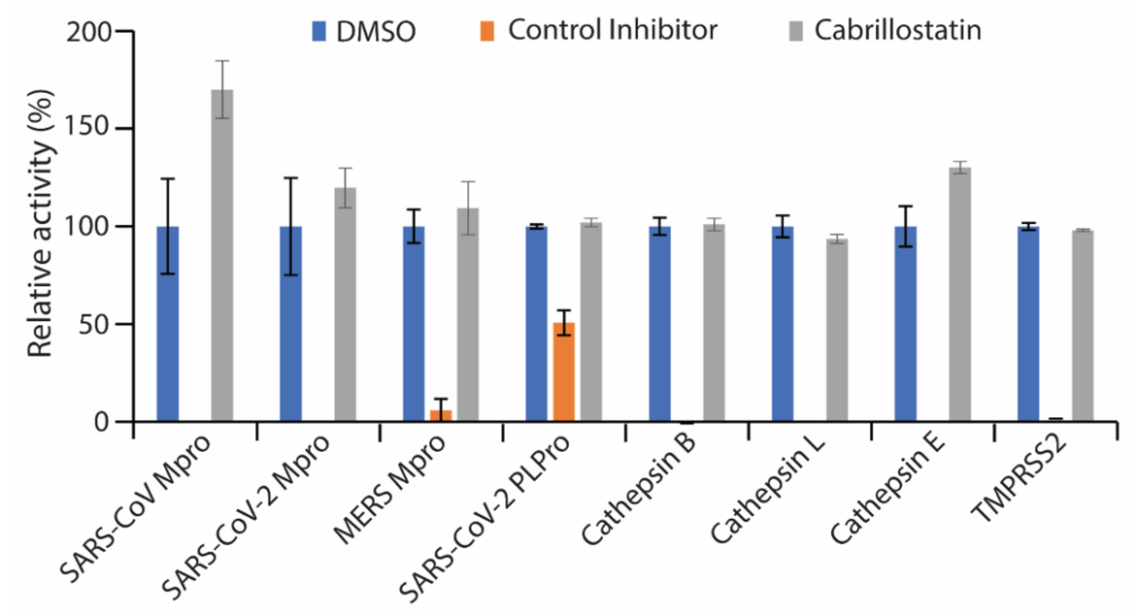

Supplementary Figure 13. Effects of cabrillostatin (1) on protease activity. (A) Cabrillostatin tested at three concentrations potentiates cathespin D activity in a concentration dependent manner. RFU (relative fluorescence units). **(B)** Cabrillostatin was inactive against eight other proteases. SARS-CoV Mpro, SARS-CoV-2 Mpro, MERS Mpro, cathepsin B, and cathepsin L are cysteine proteases. Cathepsin E is an aspartic acid protease. TMPRSS2 is a serine protease. The following control inhibitors were used: 10 μM of GC-376 for Mpro enzymes, 10 μM of compound 159 for PLpro, 10 μM of E-64 for cathepsin B and L, 10 μM of pepstatin for cathepsin E and 1 μM of camostat for TMPRSS2.

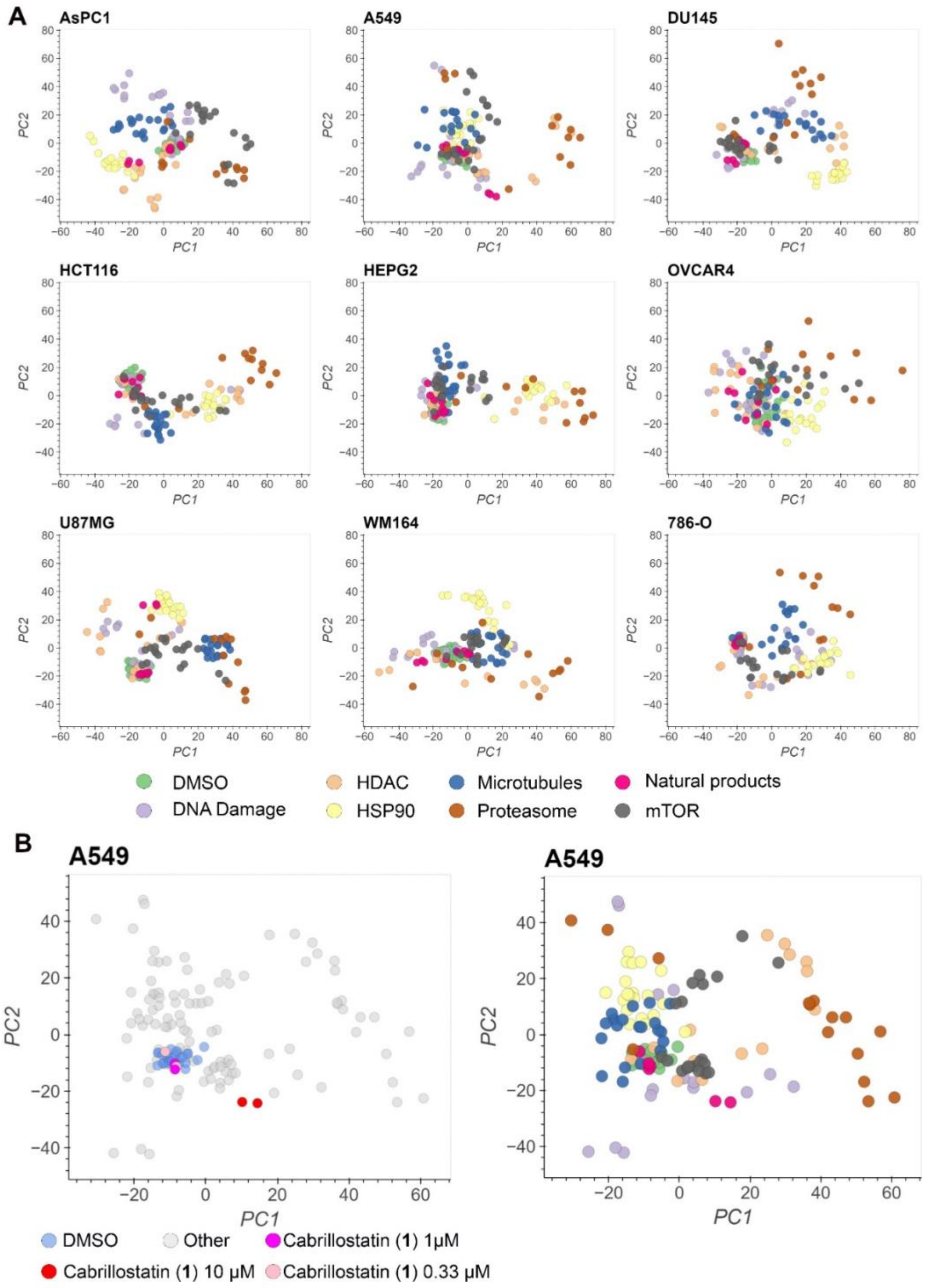

Supplementary Figure 14. Bioactivity of cabrillostatin (1) and cabrillospirals (2-3). (**A**) Principal component analyses (PCA) of high dimensional phenotypic profiles of new natural products, reference compounds/targets, and DMSO in nine diverse cell lines (data as shown in Figure 5A). Colors indicate the different compound/target categories. (**B**) PCA of high dimensional phenotypic profiles of a cabrillostatin dilution series. Cabrillostatin doses (left) and compound/target categories (right) are highlighted by color (color legend same as in **A**).

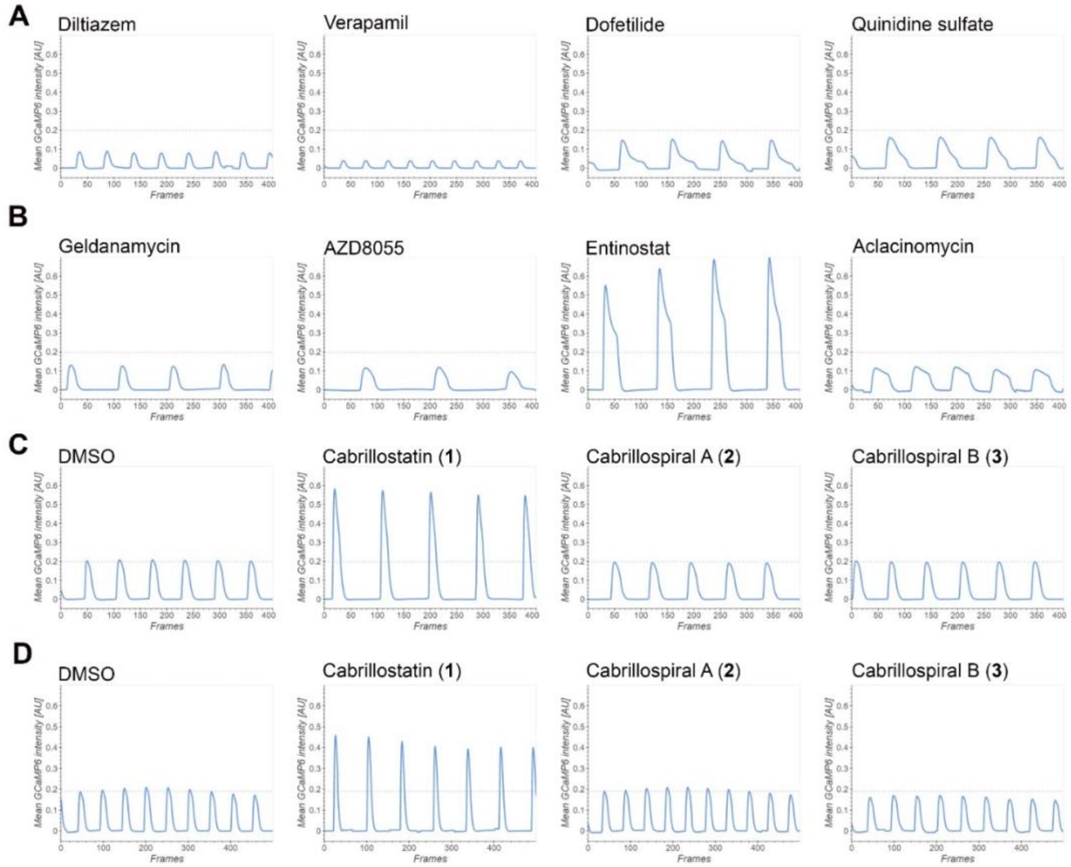

Supplementary Figure 15. Activity of cabrillostatin (1) on induced pluripotent stem cell derived cardiomyocytes (IPSC-CM). Representative Ca^{2+} transient traces of anti-arrhythmic compounds (**A**), anti-cancer drugs (**B**) or new natural products in different IPSC-CM differentiation batches (**C** and **D**).

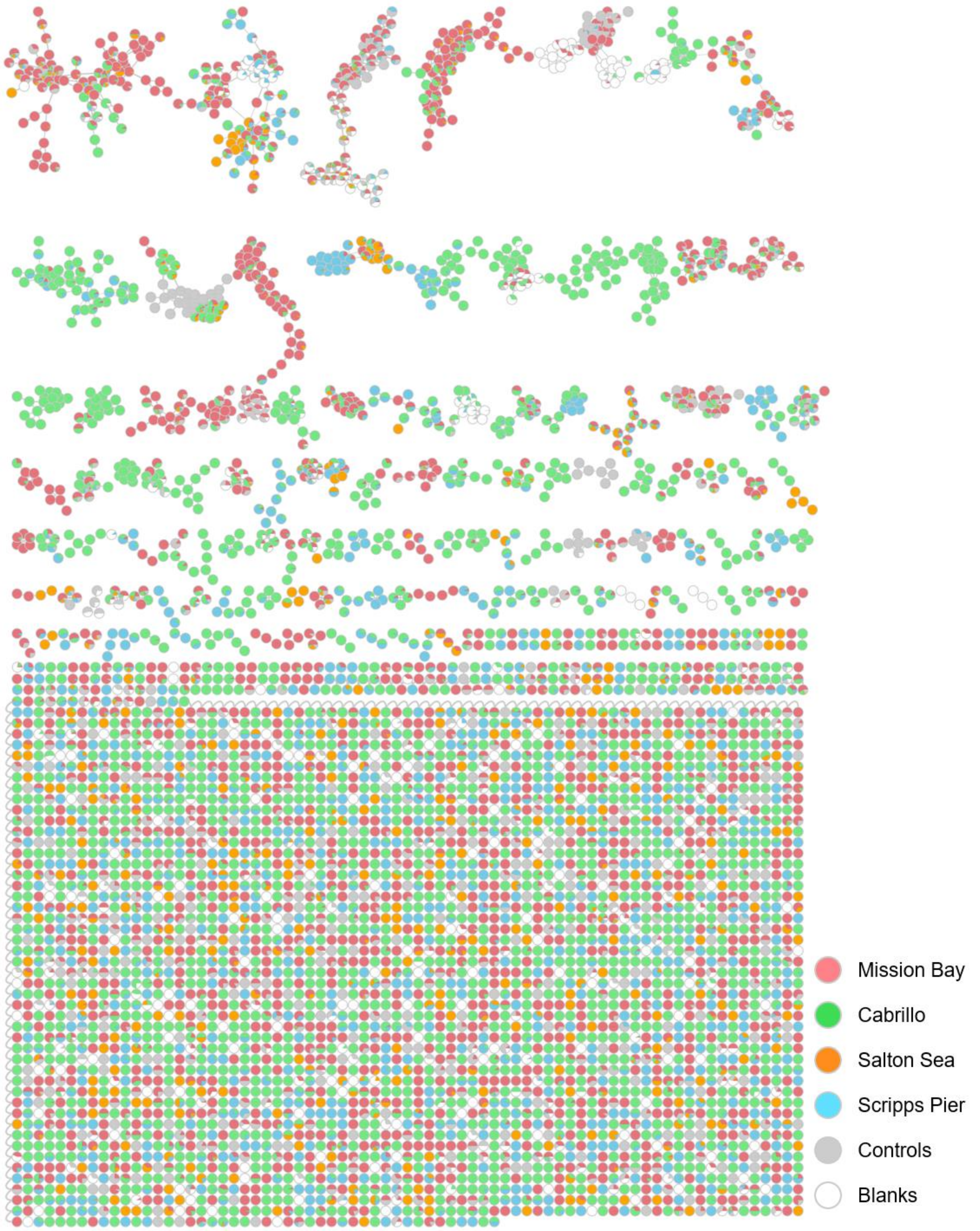

Supplementary Figure 16. Molecular network created with LCMS data of crude extracts and fractions from four different SMIRC deployment sites. Nodes represent ions and are color-coded by site.

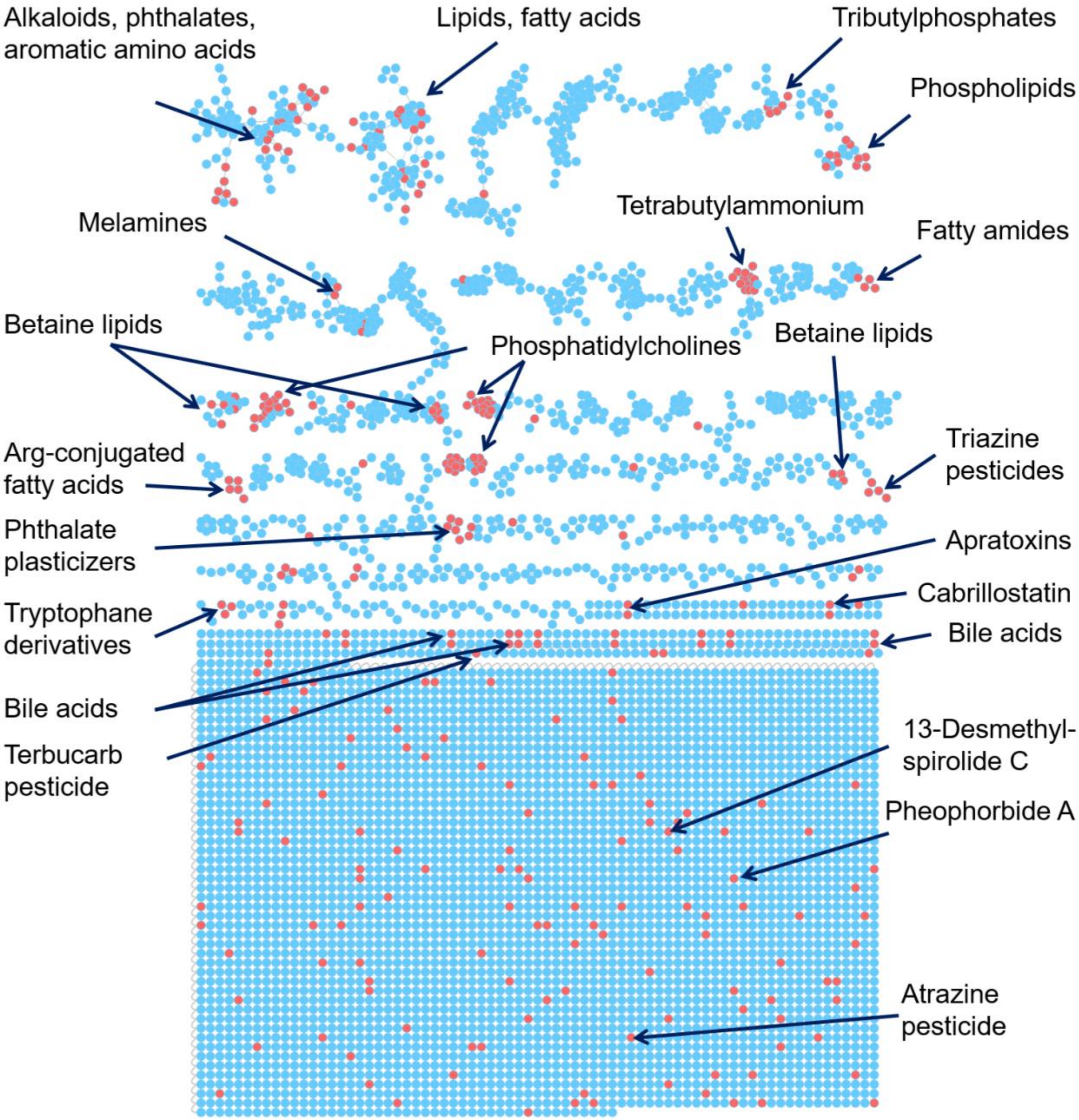

Supplementary Figure 17. Molecular network created with LCMS data of crude extracts and fractions from four different SMIRC deployment sites. Annotated nodes (annotation rate 6.33%) are shown in red. Annotated compounds and compound families are indicated with arrows. Nodes in blue represent unidentified chemical space.

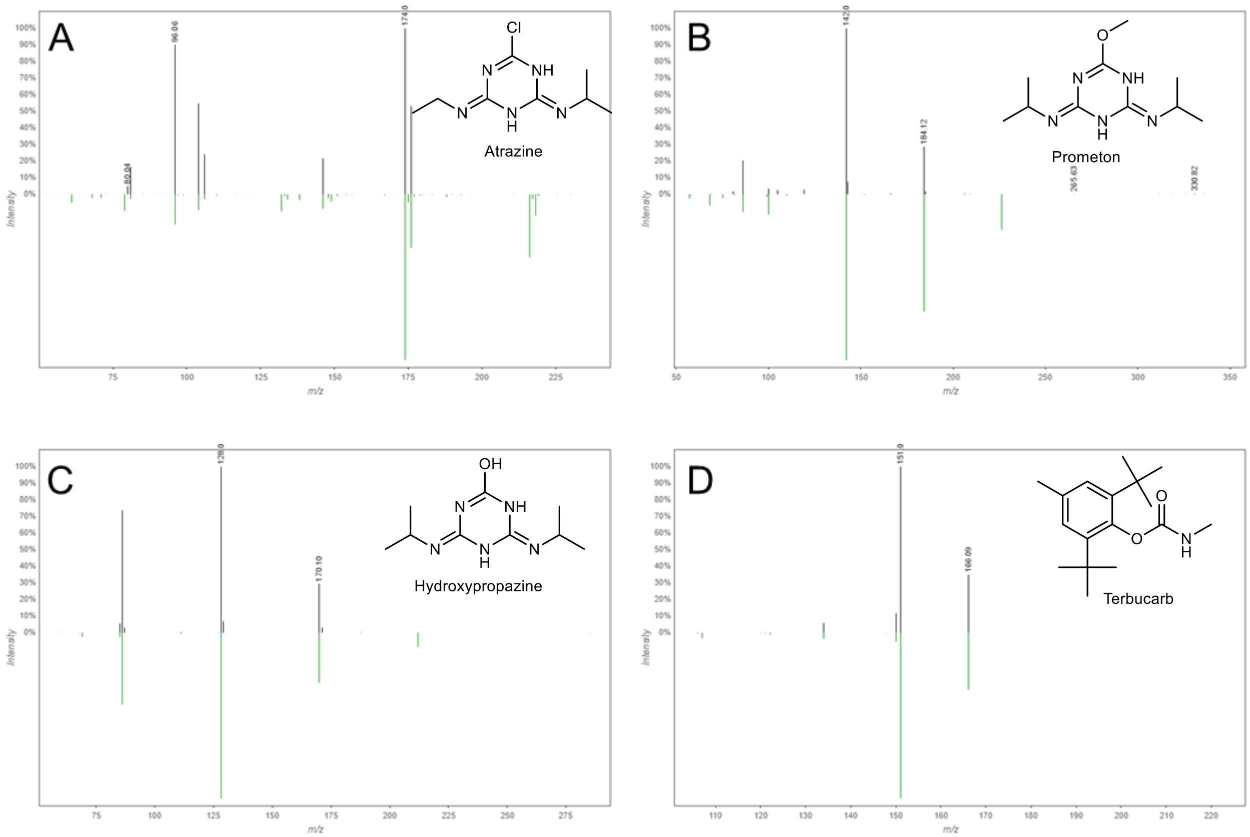

Supplementary Figure 18. GNPS mirror plots showing the detection of pesticides in SMIRC extracts. (A) Atrazine. **(B)** Prometon. **(C)** Hydroxypropazine. **(D)** Terbucarb.

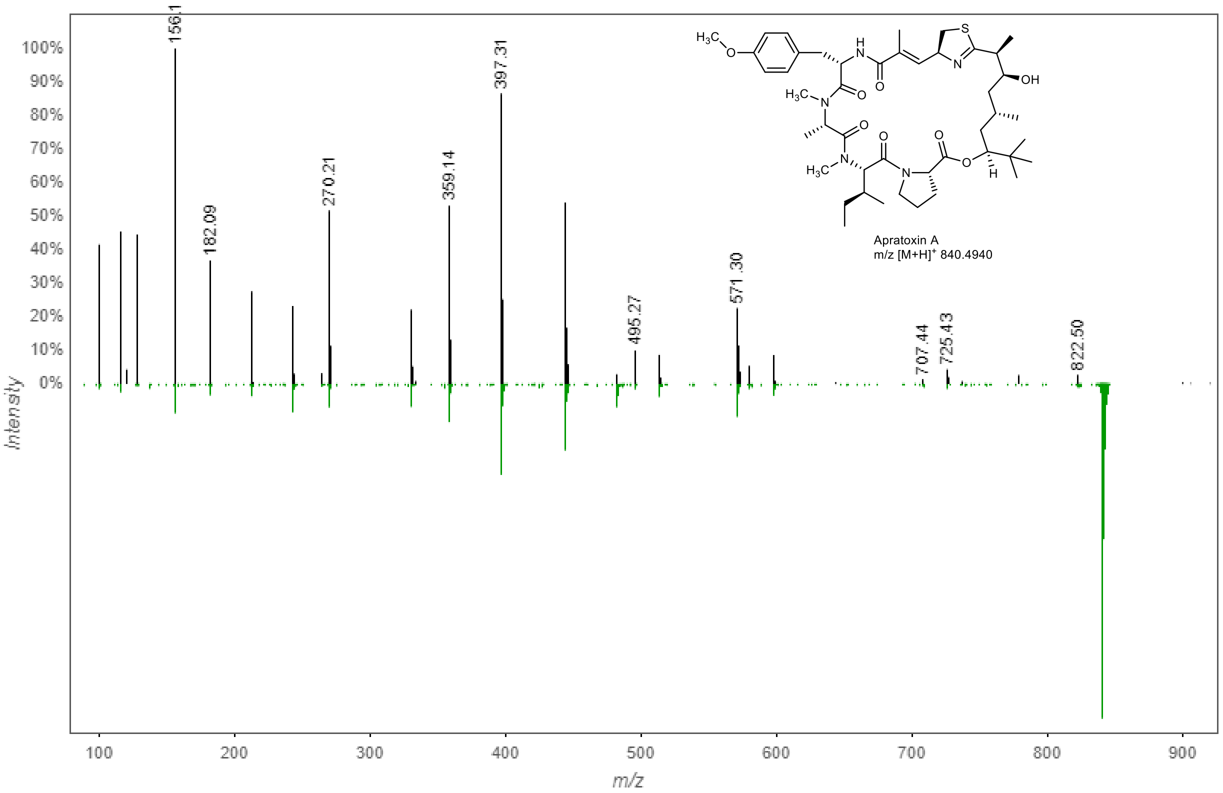

Supplementary Figure 19. GNPS mirror-plot showing the identification of apratoxin A in a SMIRC extract from the Fiesta Island, San Diego deployment site.

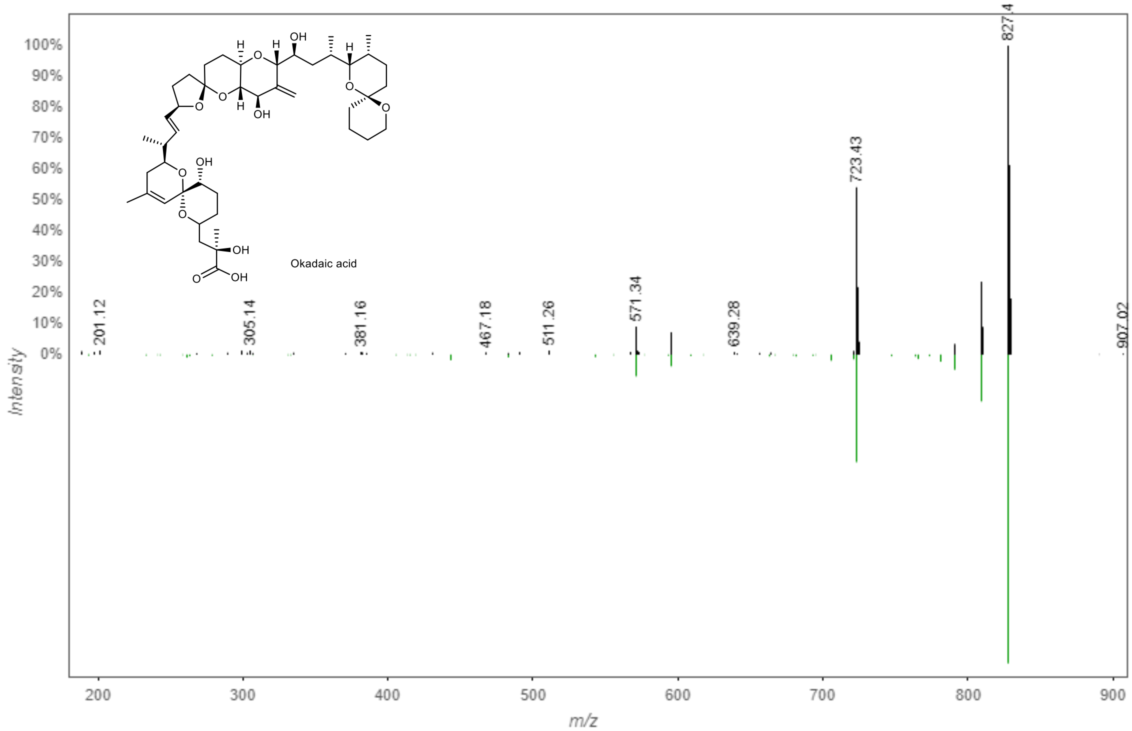

Supplementary Figure 20. GNPS mirror-plot showing the identification of okadaic acid in a SMIRC extract from the CSMR, San Diego deployment site.

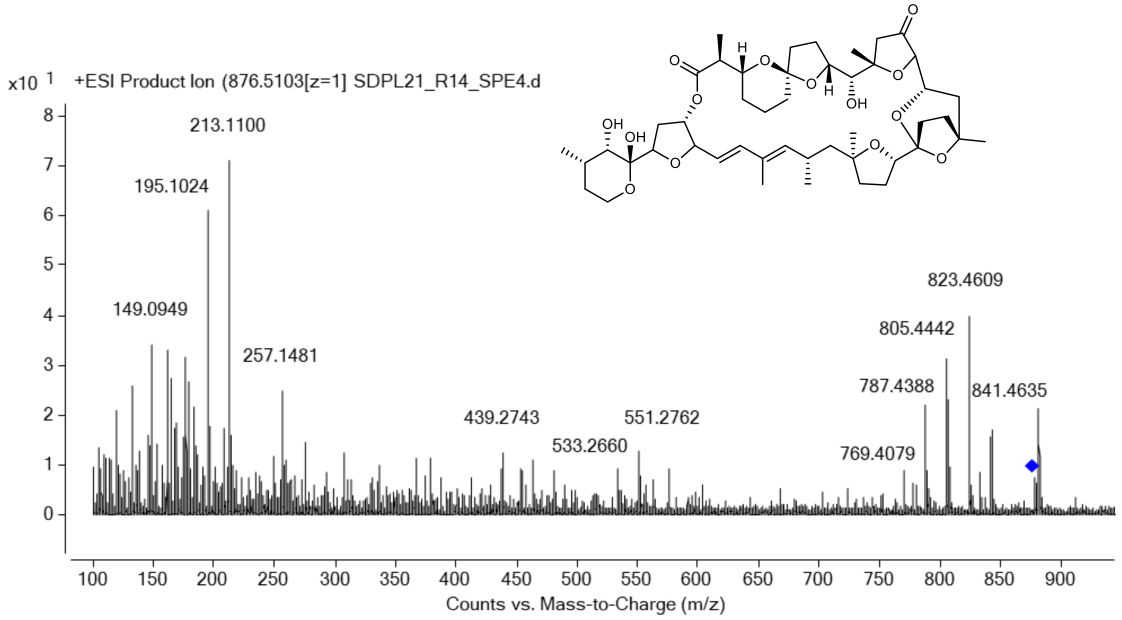

Supplementary Figure 21. MS/MS spectrum and structure of pectenotoxin-2 (PTX-2) detected in a SMIRC extract from the CSMR, San Diego deployment site. MS/MS spectrum of compound detected. The reference spectrum of PTX-2 can be found in Suzuki et al. 2003, figure 3, panel C [8].

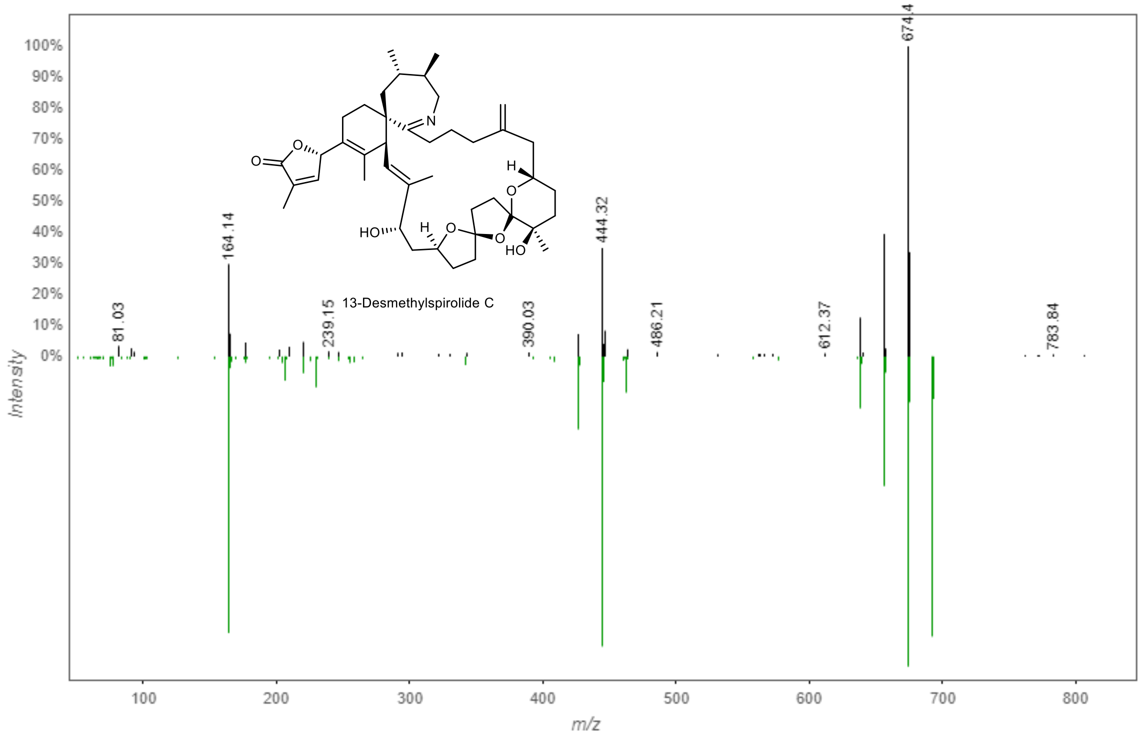

Supplementary Figure 22. A GNPS mirror-plot showing the identification of 13-desmethylspirolide in a SMIRC extract from the Scripps Pier, San Diego, deployment site.

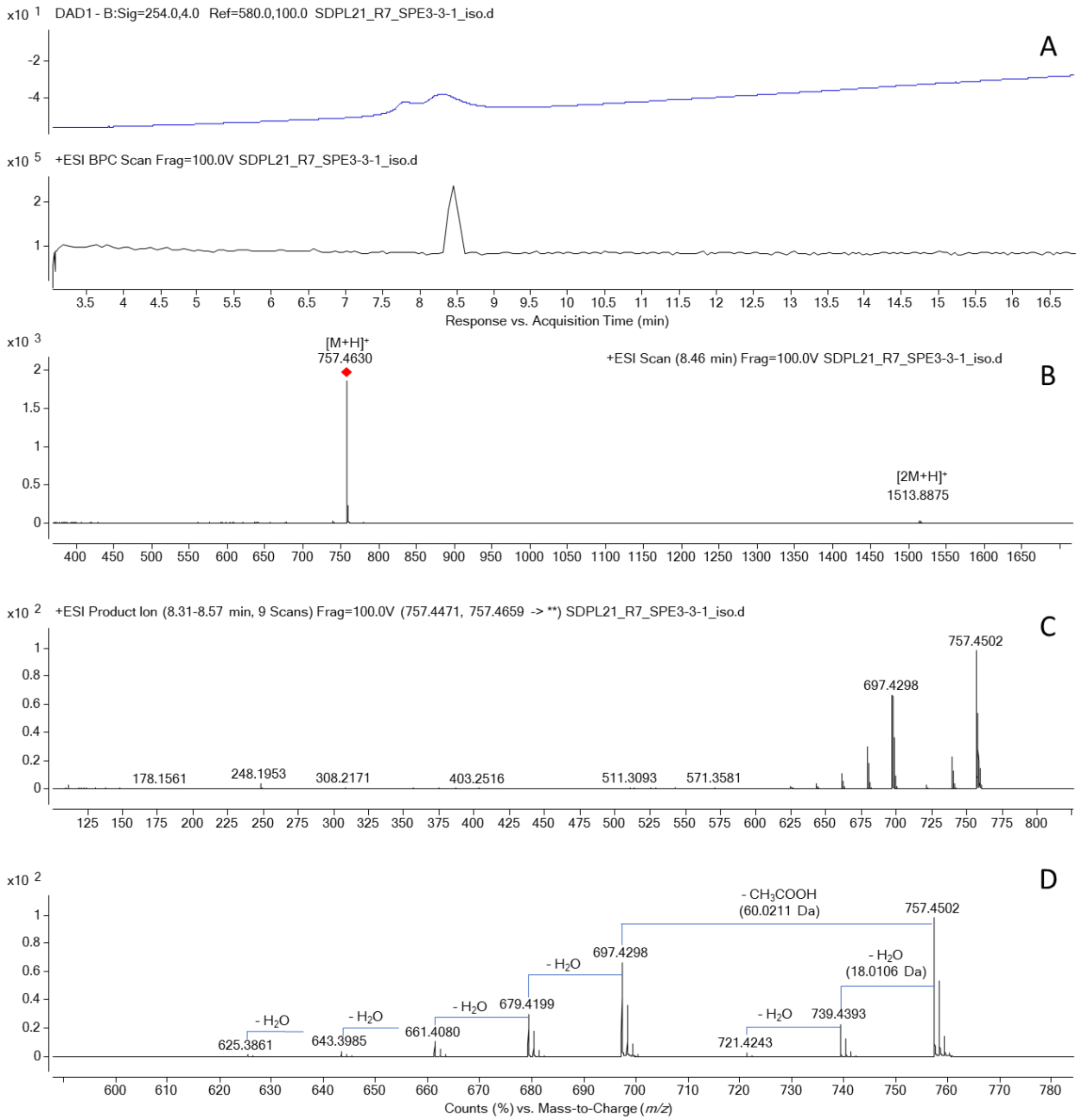

Supplementary Figure 23. LCMS of the compound *m/z* 757.463 [M+H]$^+$ (calcd. formula $C_{42}H_{64}N_2O_{10}$; -0.49 ppm). (**A**) UV 254 nm and base peak chromatograms. (**B**) MS spectrum. (**C**) MS/MS spectrum full mass range. (**D**) Expanded MS/MS spectrum with annotated neutral fragment, showing one acetate loss and four consecutive losses of H_2O. DAD (diode array detection), ESI (electrospray ionization), BPC (base peak chromatogram).

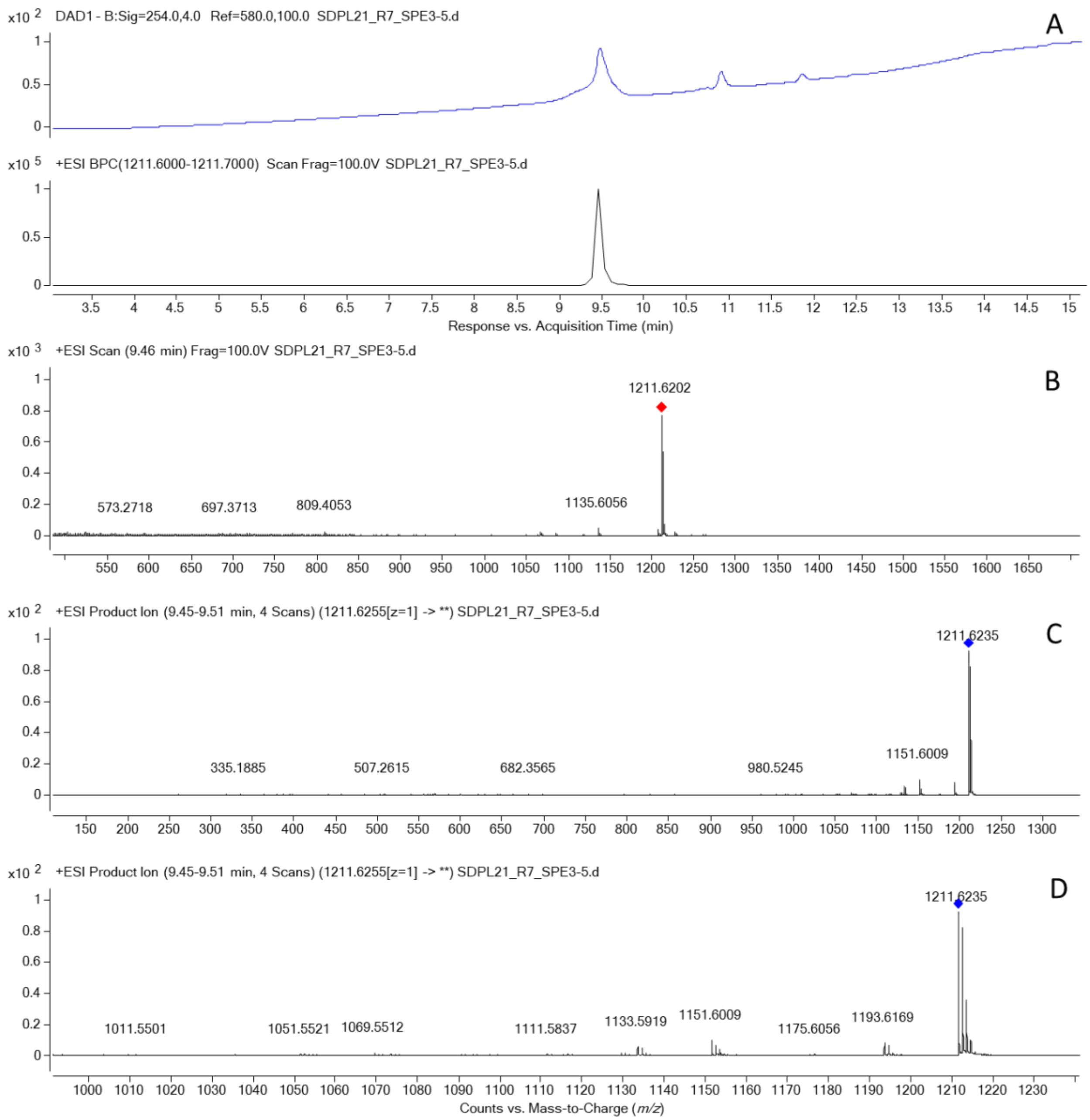

Supplementary Figure 24. LCMS of HPLC fraction containing semi pure compound *m/z* 1211.6202 $[M+Na]^+$ (exact *m/z* 1211.6324, calcd. MF $C_{63}H_{96}O_{21}$, -1.02 ppm). (**A**) UV (254 nm) and extracted ion chromatograms. (**B**) MS spectrum. (**C**) MS/MS spectrum of the $[M+Na]^+$ precursor ion. (**D**) Expanded MS/MS spectrum showing neutral losses of an acetate and several H_2O. Overall limited fragmentation can indicate a polyether-like carbon skeleton. DAD (diode array detection), ESI (electrospray ionization), BPC (base peak chromatogram).

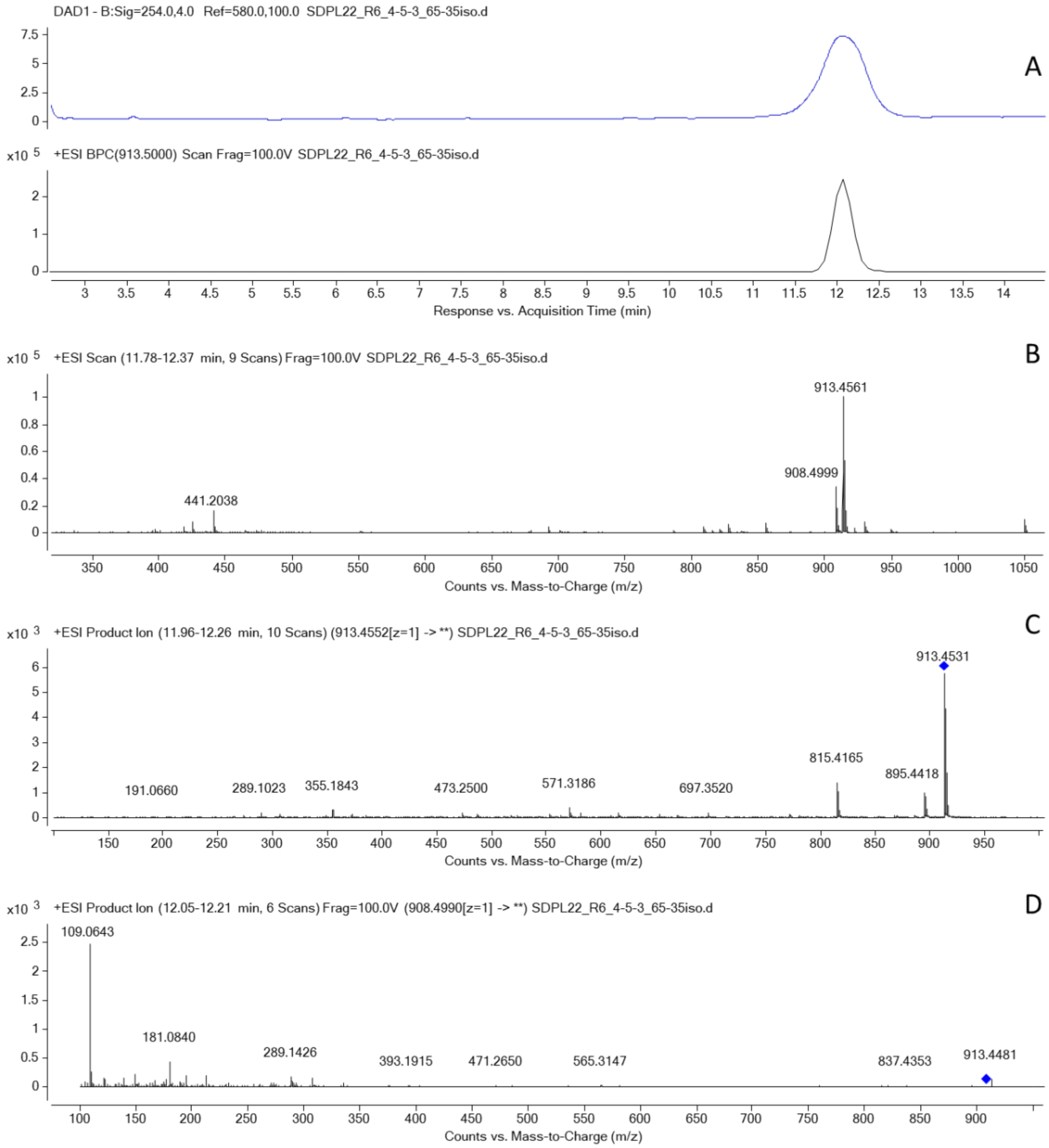

Supplementary Figure 25. LCMS of semi pure compound *m/z* 913.4561 [M+Na]$^+$ (calcd formula $C_{47}H_{70}O_{16}Na$; 0.54 ppm). (**A**) UV (254 nm) and extracted ion chromatograms. (**B**) MS spectrum. (**C**) MS/MS spectrum of *m/z* 913.4561 [M+Na]$^+$. (**D**) MS/MS spectrum of *m/z* 908.4990 [M+NH_4 Overall limited fragmentation can indicate a polyether-like carbon skeleton. DAD (diode array detection), ESI (electrospray ionization), BPC (base peak chromatogram).

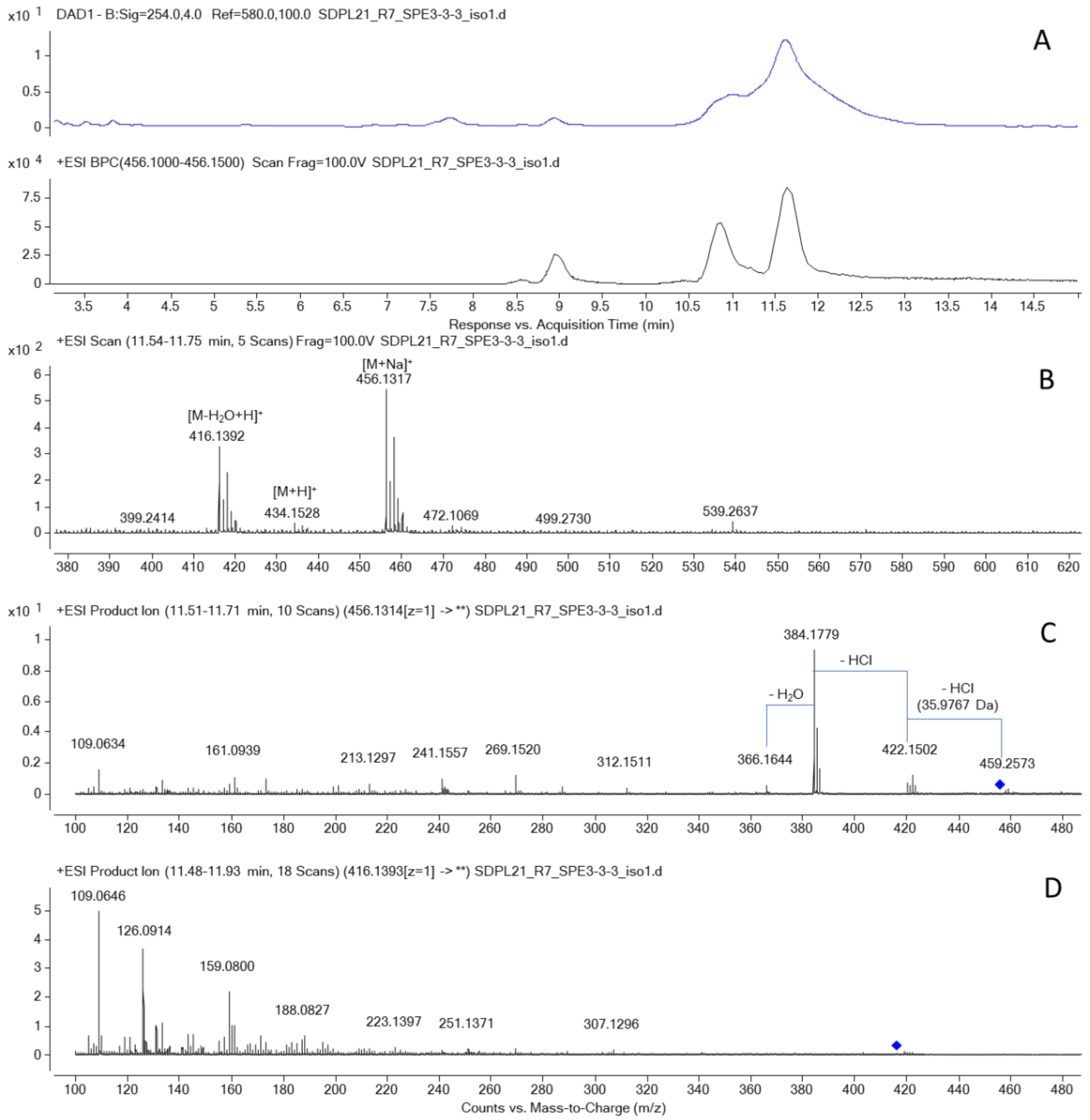

Supplementary Figure 26. LCMS of HPLC fraction containing four *m/z* 456.1317 [M+Na]$^+$ (calcd formula $C_{20}H_{29}Cl_2NO_5Na$; 0.44 ppm) isomers. (**A**) UV (254 nm) and extracted ion chromatograms. (**B**) MS spectrum of the major isomer (RT 11.7 min). (**C**) MS/MS spectrum of [M+Na]$^+$ precursor, major fragments resut from HCl and H_2O losses. (**D**) MS/MS spectrum of [M-H_2O+H]$^+$ precursor. Major peaks are highly unsaturated aromatic fragments *m/z*s 109.0646 (C_7H_8O), 159.0800 ($C_{11}H_{10}O$), and alkaloid fragments *m/z*s 188.0827 ($C_9H_{14}ClNO$) and 126.0914 ($C_7H_{11}NO$). DAD (diode array detection), ESI (electrospray ionization), BPC (base peak chromatogram).

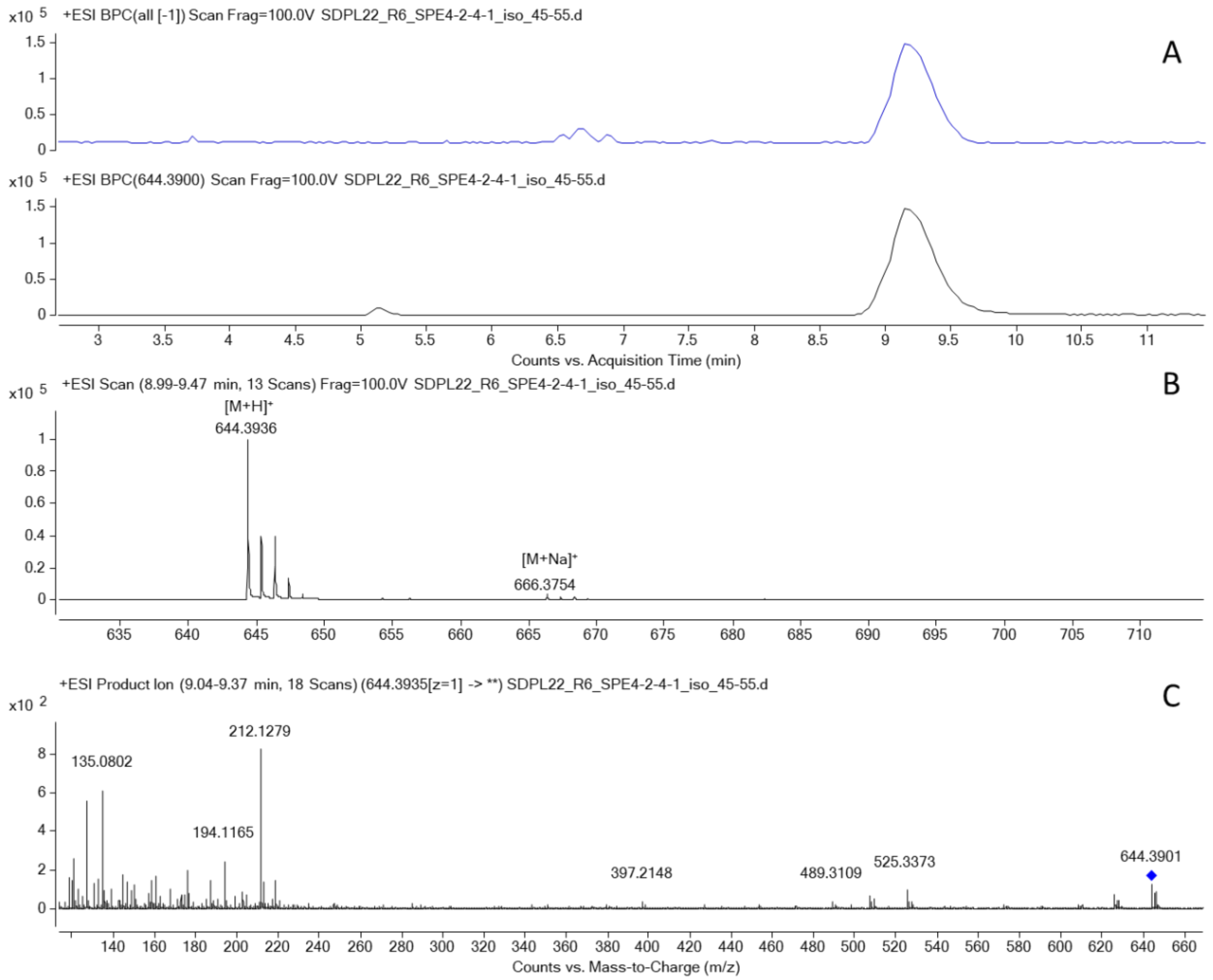

Supplementary Figure 27. LCMS of semi-pure compound *m/z* 644.3936 [M+H]$^+$ (calcd. MF $C_{34}H_{58}ClNO_8$, 1.91 ppm). (**A**) Base peak and extracted ion (*m/z* 644.39) chromatograms. (**B**) MS spectrum showing protonated [M+H]$^+$ and sodiated [M+Na]$^+$ ions. (**C**) MS/MS spectrum of the [M+H]$^+$ precursor ion shows multiple fragments resulting from H_2O losses and major nitrogen containing fragment *m/z* 212.1279 (calcd. MF $C_{11}H_{17}NO_3$). ESI (electrospray ionization), BPC (base peak chromatogram).

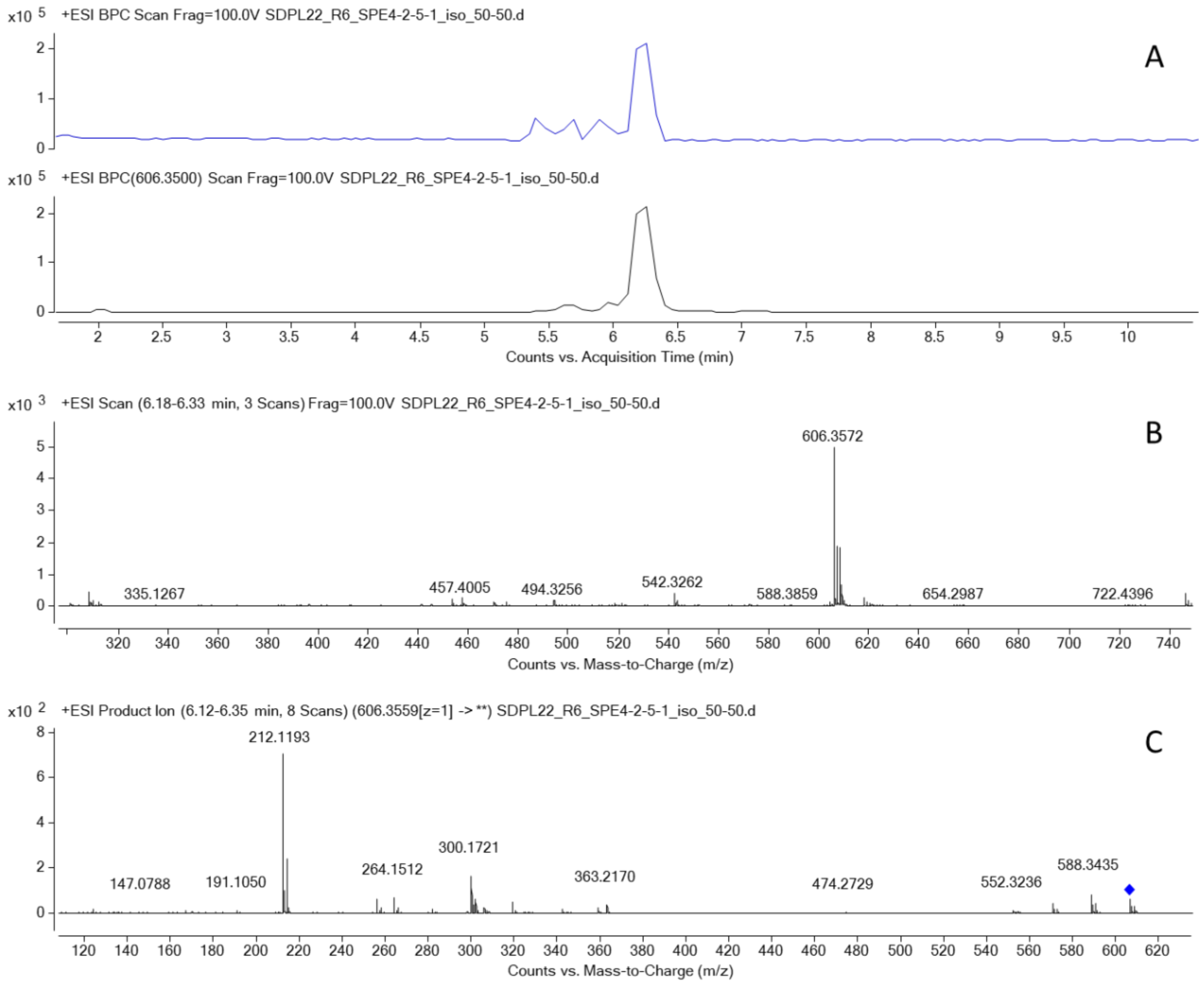

Supplementary Figure 28. LCMS of HPLC fraction containing compound *m/z* 606.3572 [M+H]$^+$ (calcd. formula $C_{34}H_{52}ClNO_6$; 0.67 ppm). (**A**) Base peak and extracted ion (*m/z* 606.35) chromatograms. (**B**) MS spectrum. (**C**) MS/MS spectrum of the [M+H]$^+$ precursor ion suggests relatedness to the compound *m/z* 644.3936 [M+H]$^+$. ESI (electrospray ionization), BPC (base peak chromatogram).

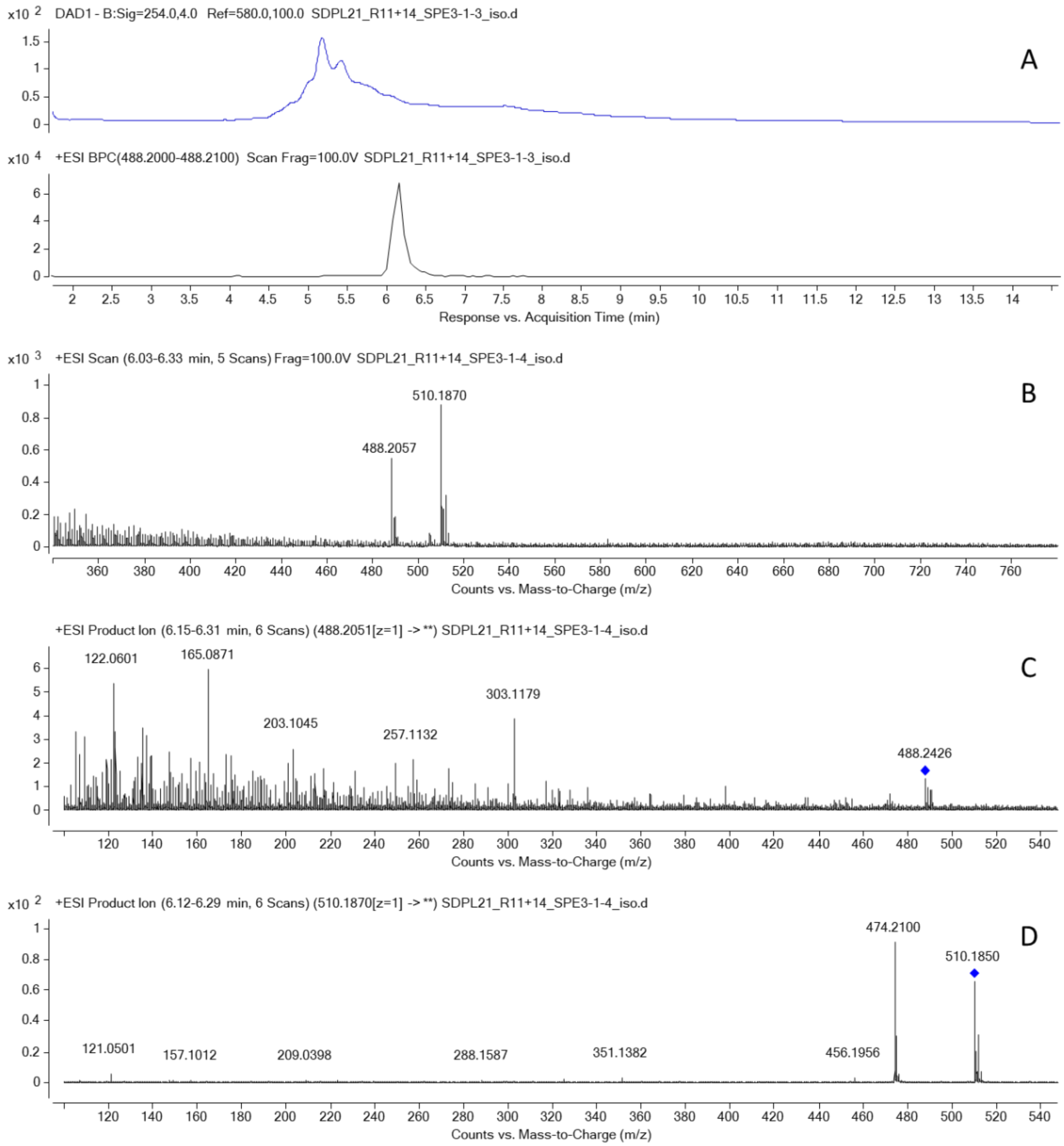

Supplementary Figure 29. LCMS of HPLC fraction containing compound *m/z* 488.2057 [M+H]$^+$ (calcd. formula $C_{23}H_{34}ClNO_8$; -2.18 ppm). (**A**) UV 254 nm and extracted ion (*m/z* 488.2-488.21) chromatograms. (**B**) MS spectrum showing protonated and sodiated ions. (**C**) MS/MS spectrum of [M+H]$^+$ precursor ion. (**D**) MS/MS spectrum of [M+Na]$^+$ precursor ion. DAD (diode array detection), ESI (electrospray ionization), BPC (base peak chromatogram).

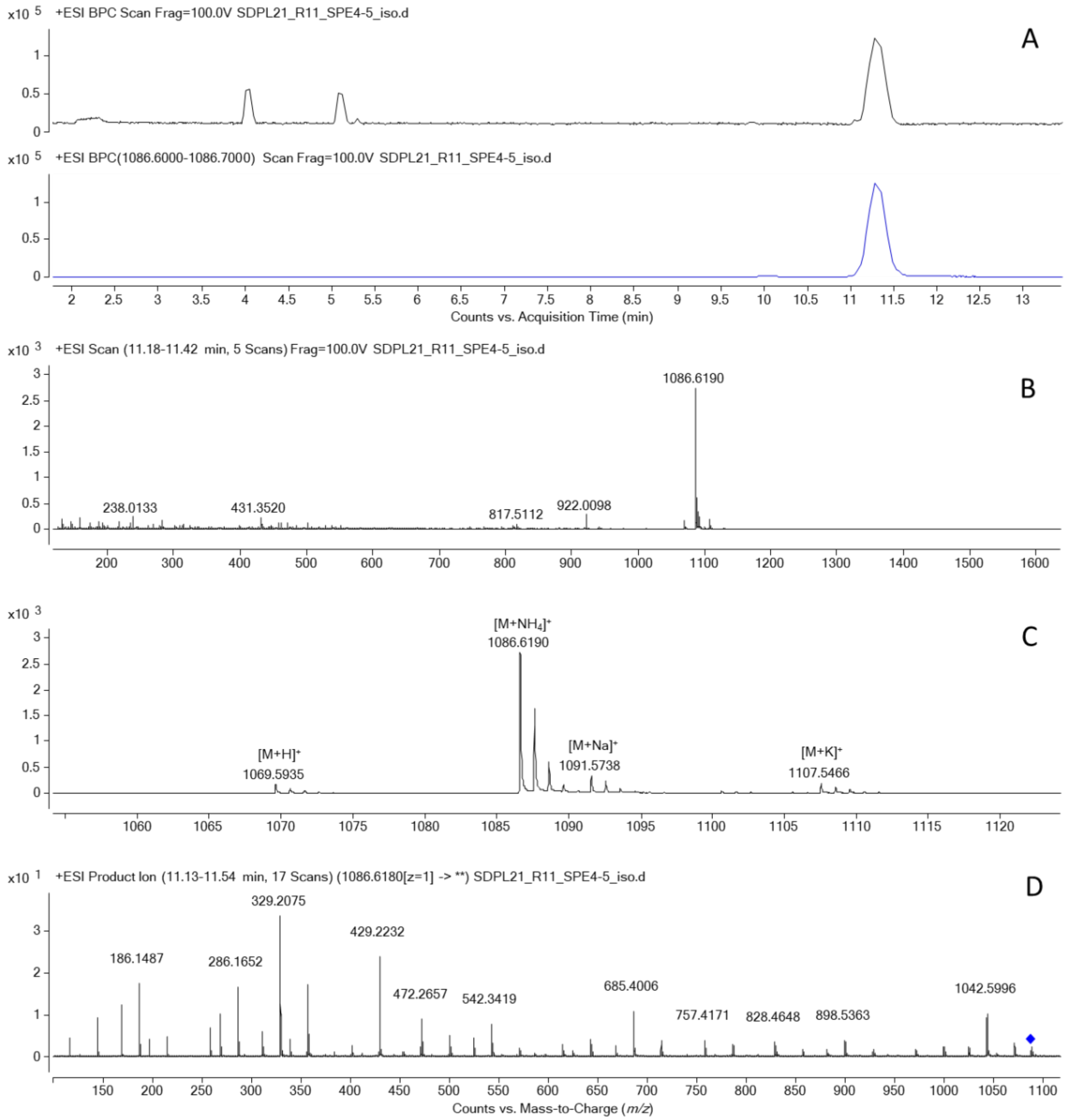

Supplementary Figure 30. LCMS of the semi pure compound *m/z* 1086.6190 $[M+NH_4]^+$ (calcd. MF $C_{52}H_{80}N_{10}O_{14}$, -0.34 ppm). (**A**) BPC and extracted ion (*m/z* 1089.6-1086.7) chromatograms. (**B**) MS spectrum. (**C**) Narrow range MS spectrum showing additional ionizations $[M+H]^+$, $[M+Na]^+$ and $[M+K]^+$. (**D**) MS/MS spectrum of the $[M+NH_4]^+$ precursor ion shows extensive fragmentation resembling a peptide. ESI (electrospray ionization), BPC (base peak chromatogram).

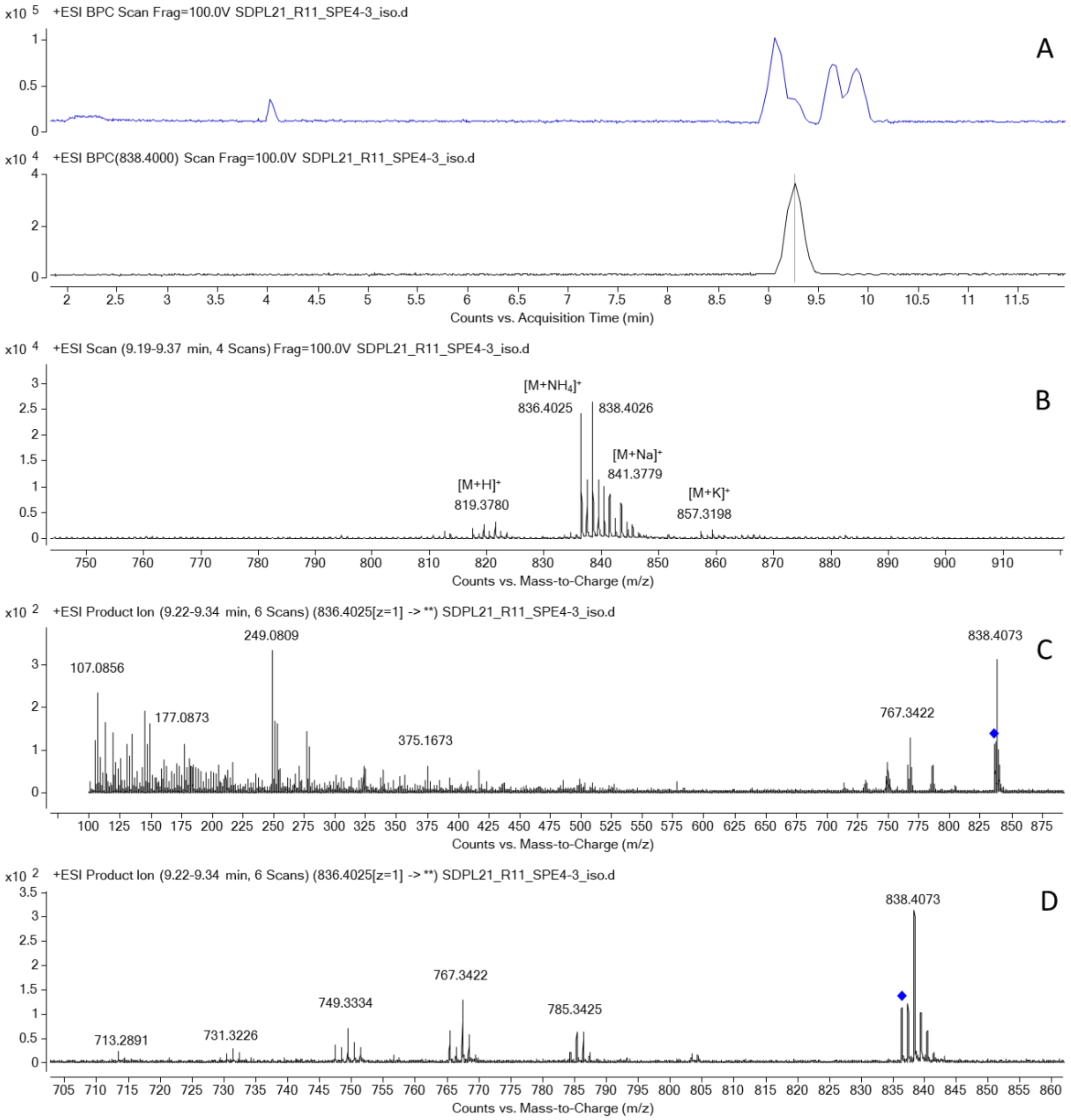

Supplementary Figure 31. LCMS of HPLC fraction containing compound *m/z* 836.4025 $[M+NH_4]^+$ (calcd. MF $C_{42}H_{65}Cl_3O_9$, -0.89 ppm). (**A**) Base peak and extracted ion (*m/z* 838.40) chromatograms. (**B**). MS spectrum showing $[M+H]^+$, $[M+Na]^+$ and $[M+K]^+$. (**C**) MS/MS spectrum of the $[M+NH_4]^+$ precursor. Of note is the overall similarity with the MS/MS spectrum of cabrillospiral A (**2**, Supplementary Figure 6) possibly indicating related compound class. (**D**) Expanded MS/MS spectrum of the $[M+NH_4]^+$ shows six consecutive losses of H_2O. ESI (electrospray ionization), BPC (base peak chromatogram).

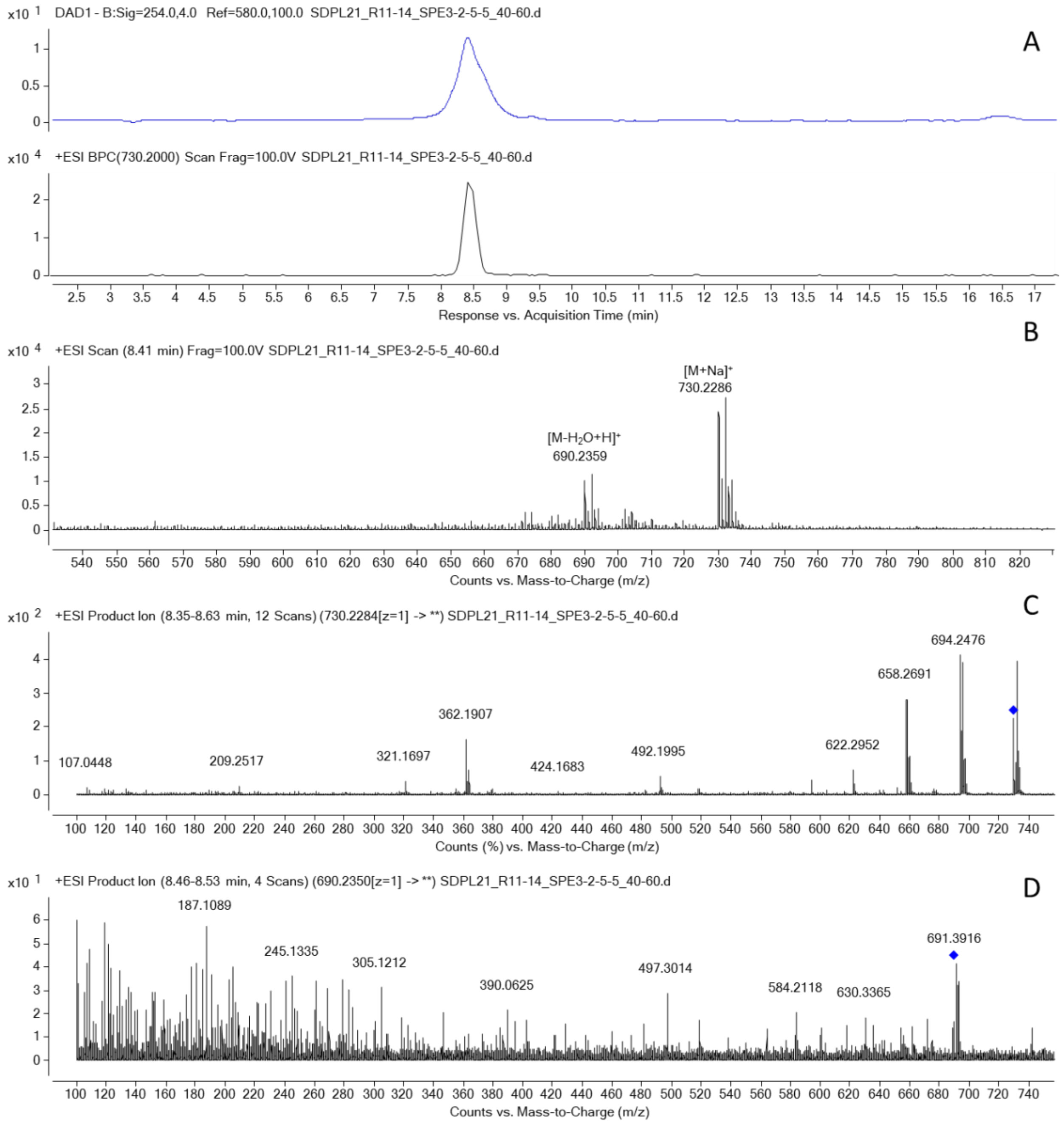

Supplementary Figure 32. LCMS of semi pure compound *m/z* 730.2286 $[M+Na]^+$ (calcd formula $C_{33}H_{48}Cl_3NO_9Na$; -0.12 ppm). (**A**) UV (254 nm) and extracted ion chromatograms. (**B**) MS spectrum showing $[M-H_2O+H]^+$ and $[M+Na]^+$ ionizations. (**C**) MS/MS spectrum of $[M+Na]^+$ precursor ion. Major fragments result from three consecutive HCl losses. (**D**) MS/MS spectrum of the $[M-H_2O+H]^+$ precursor ion. DAD (diode array detection), ESI (electrospray ionization), BPC (base peak chromatogram).

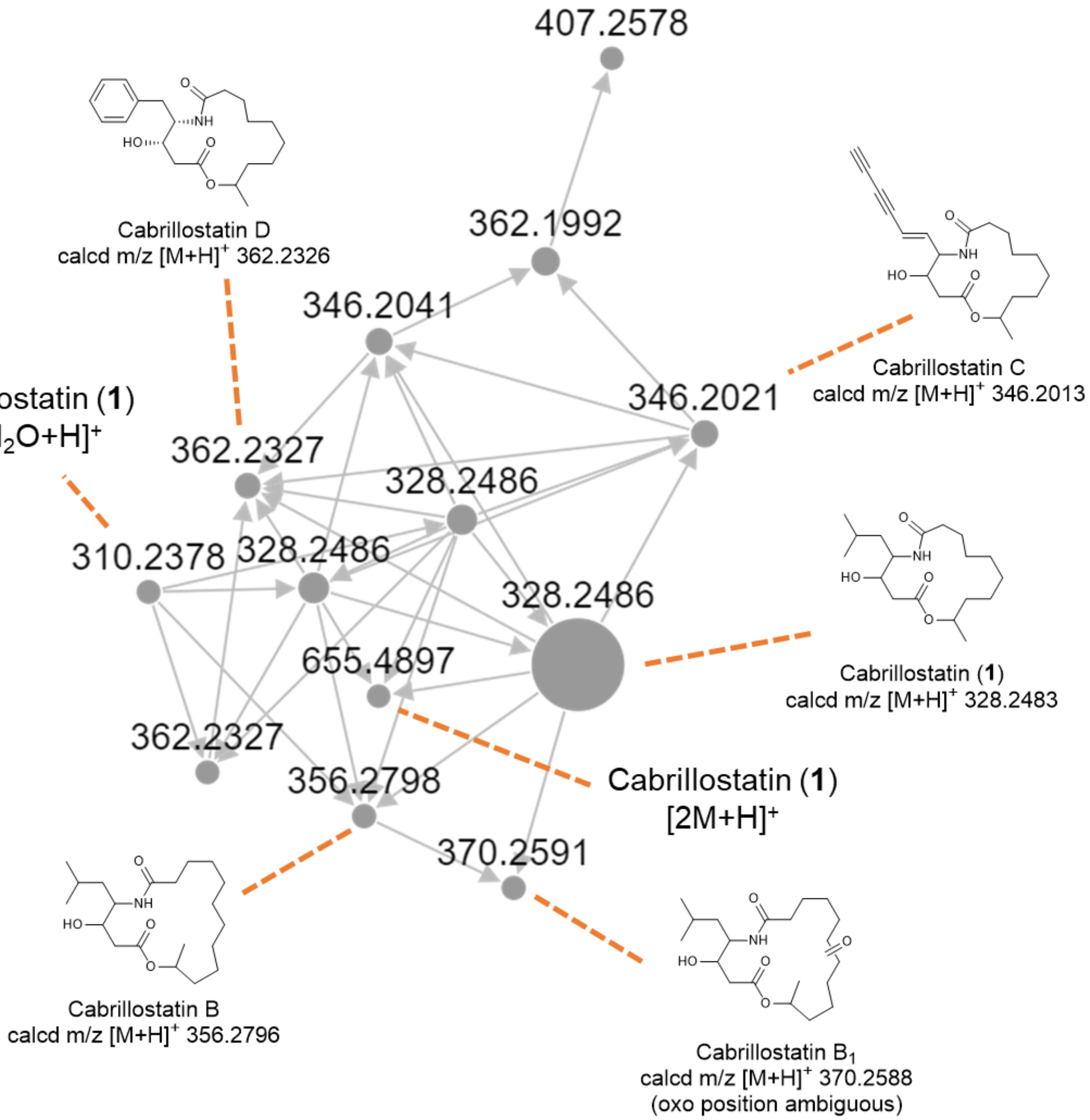

Supplementary Figure 33. Detection of cabrillostatin (1) in a molecular network constructed from an LCMS study of DOM. Water samples collected at Imperial Beach, San Diego, the Tijuana River estuary, and the Scripps Pier (*47*). Nodes (parent ions) are connected with edges (arrows) based on similarities in MS/MS fragmentation spectra. The structures of new compounds (cabrillostatins B, B1, C, and D) are proposed based on manual analysis of the MS and MS/MS spectra and comparison with those of **1**. Node size represents the sum precursor ion intensity and correlates with the relative abundance of the compounds.

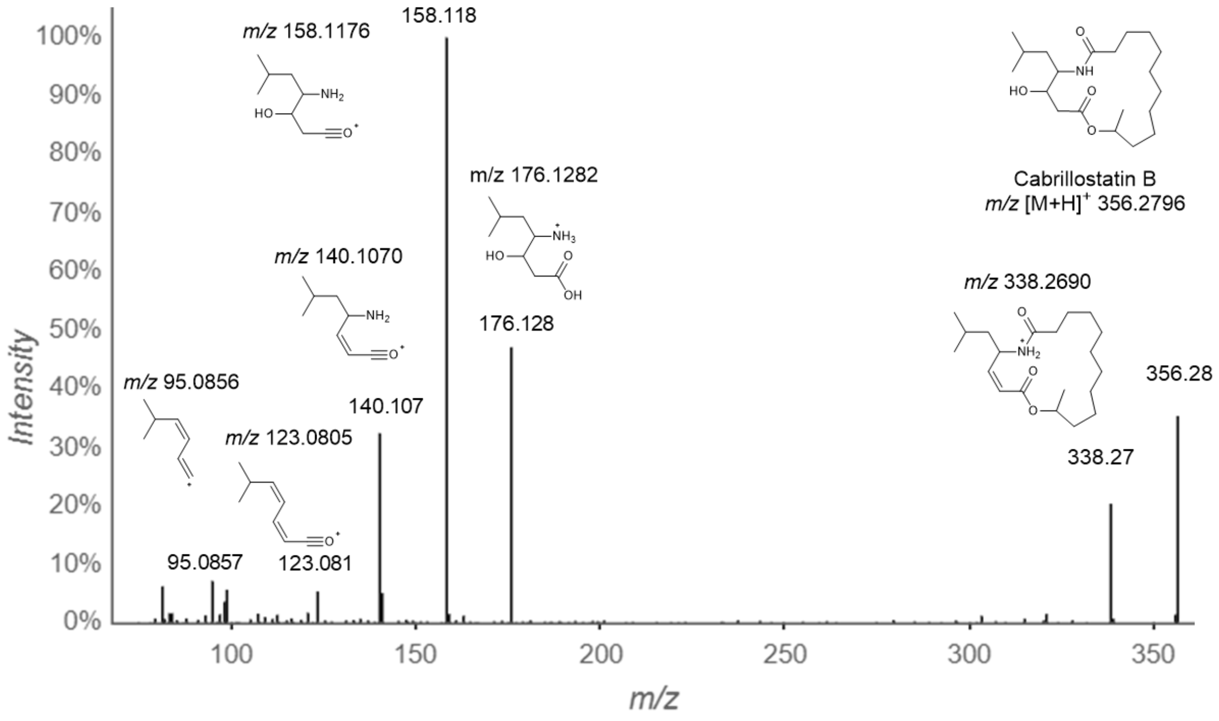

Supplementary Figure 34. Annotated MS/MS spectrum of cabrillostatin B.

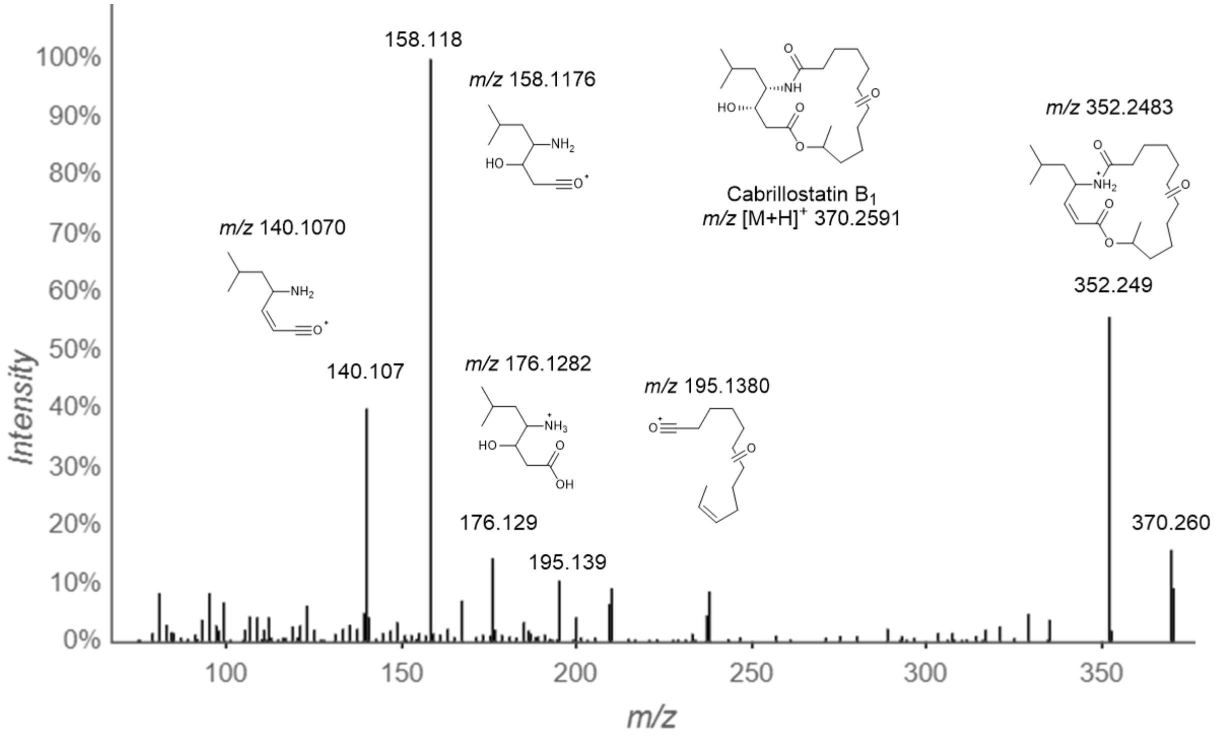

Supplementary Figure 35. Annotated MS/MS spectrum of cabrillostatin B1.

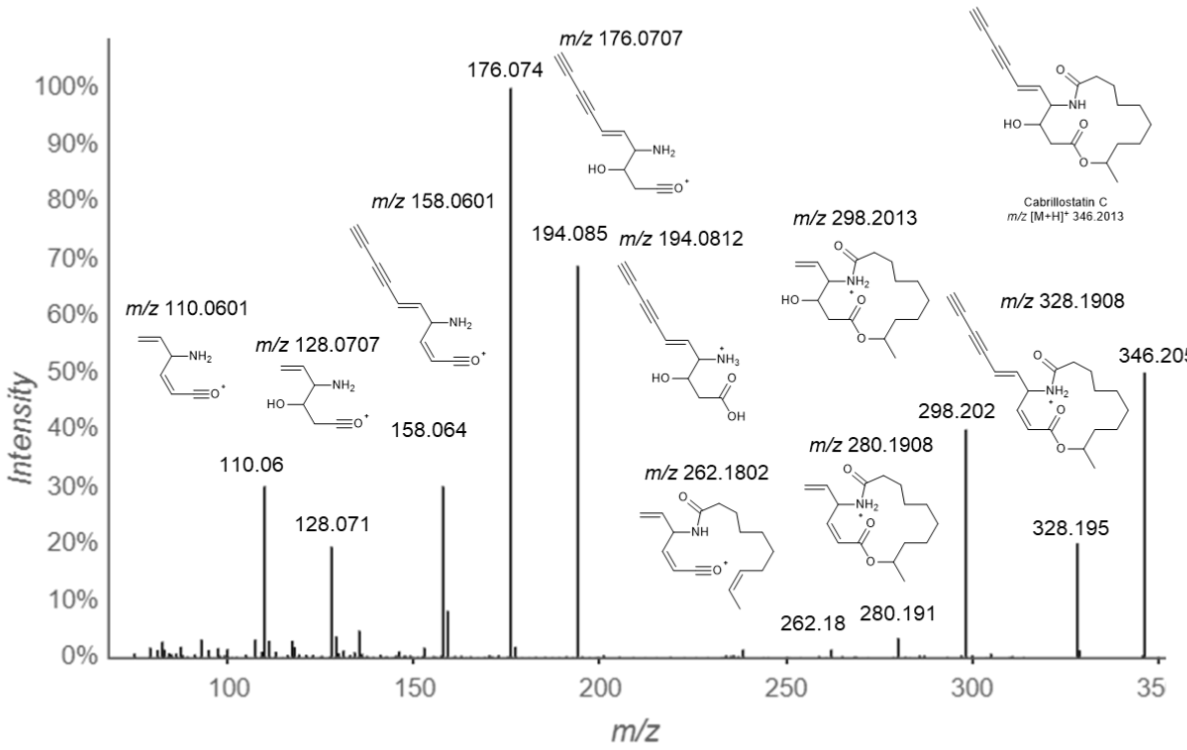

Supplementary Figure 36. Annotated MS/MS spectrum of cabrillostatin C.

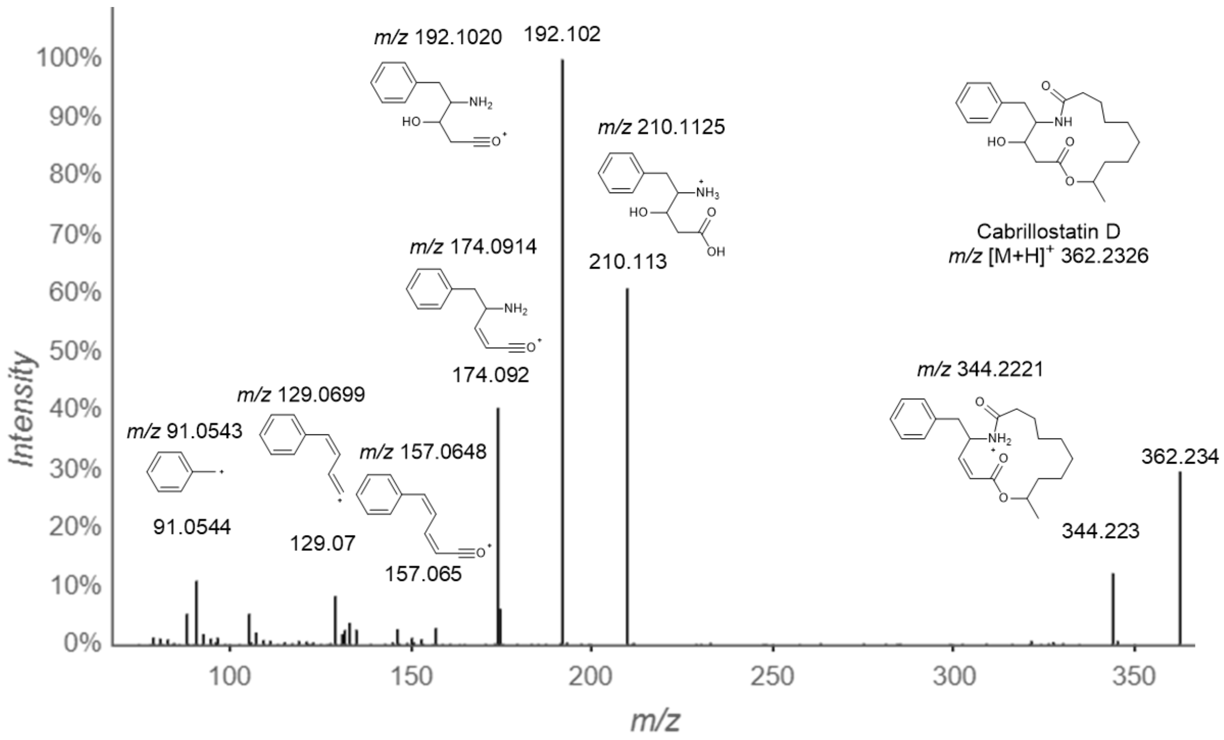

Supplementary Figure 37. Annotated MS/MS spectrum of cabrillostatin D.

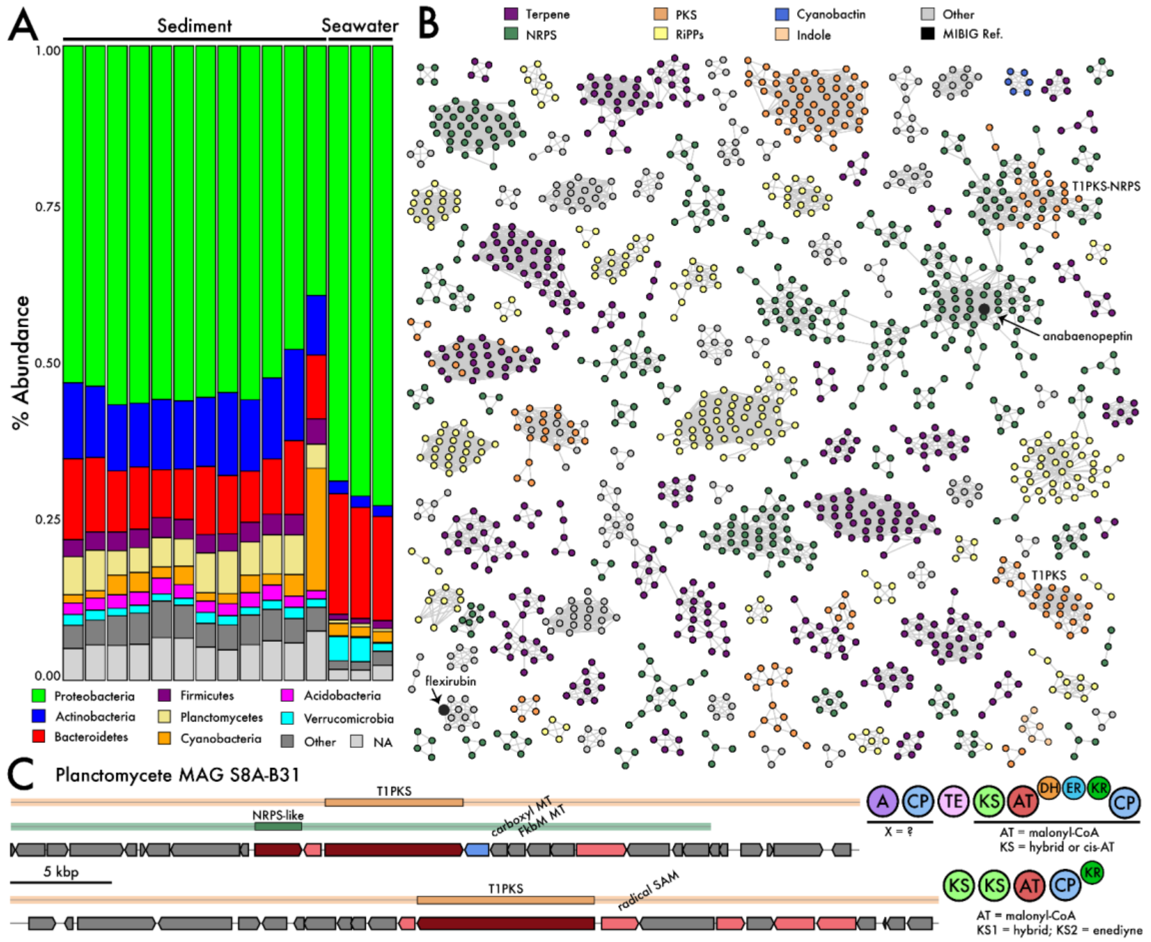

Supplementary Figure 38. CSMR microbial community composition and biosynthetic potential. (**A**) Relative abundances of the eight most abundant bacterial phyla across sediment and seawater samples. (**B**) Biosynthetic potential of bacterial communities clustered into gene cluster families (GCFs). Each node represents a predicted biosynthetic gene cluster (BGC) colored by its predicted product (reference MIBiG nodes in black). Singleton and doubleton GCFs not shown. (**C**) Planctomycete MAG (metagenome assembled genome) encodes two type 1 polyketide synthetase (T1PKS) BGCs (PKS domains shown) that are candidates for the biosynthesis of compounds **2-3**. A (adenylation domain), CP (carrier protein), TE (thioesterase), KS (ketosynthase), AT (acyl transferase), DH (dehydratase), ER (enoyl reductase), KR (keto reductase). AT substrate specificity and KS classification shown.

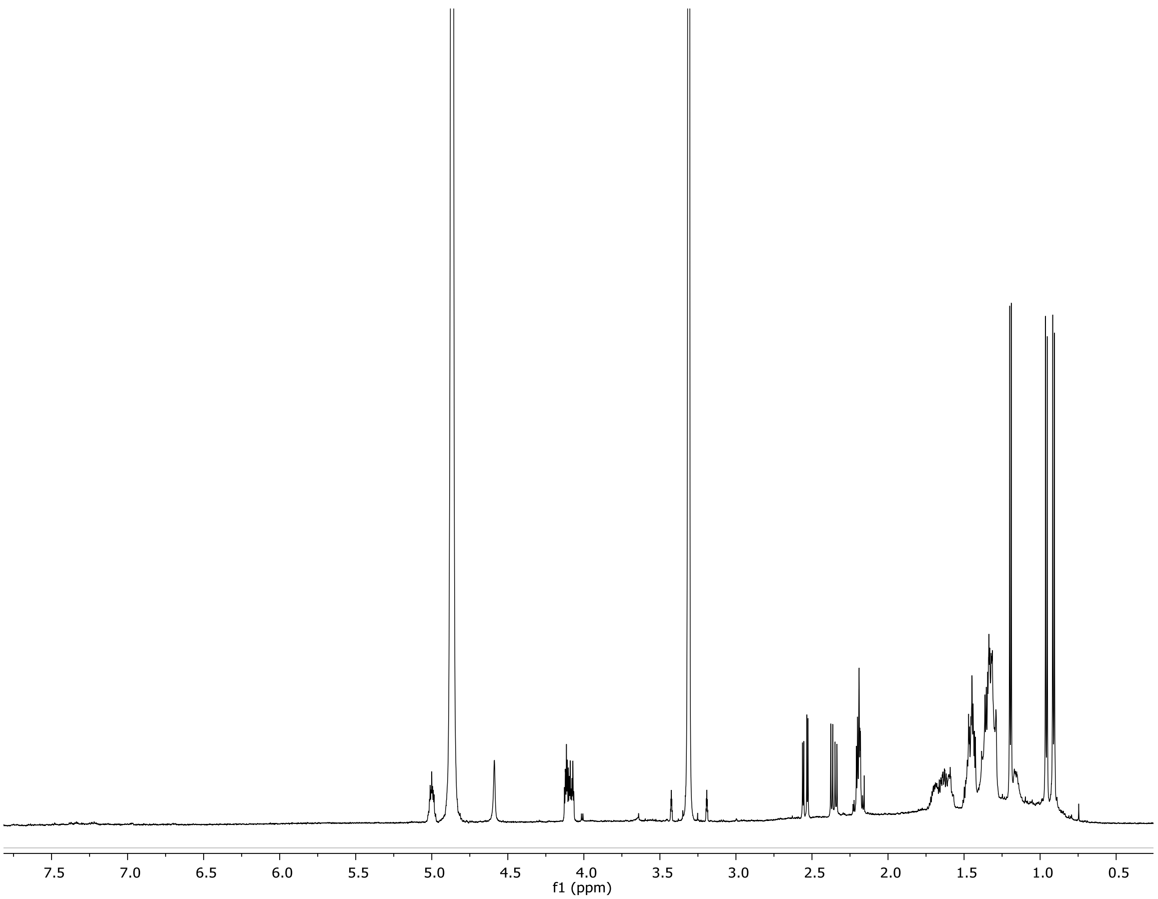

Supplementary Figure 39. ^{1}H NMR spectrum of cabrillostatin (1) in CD_3OD (600 MHz).

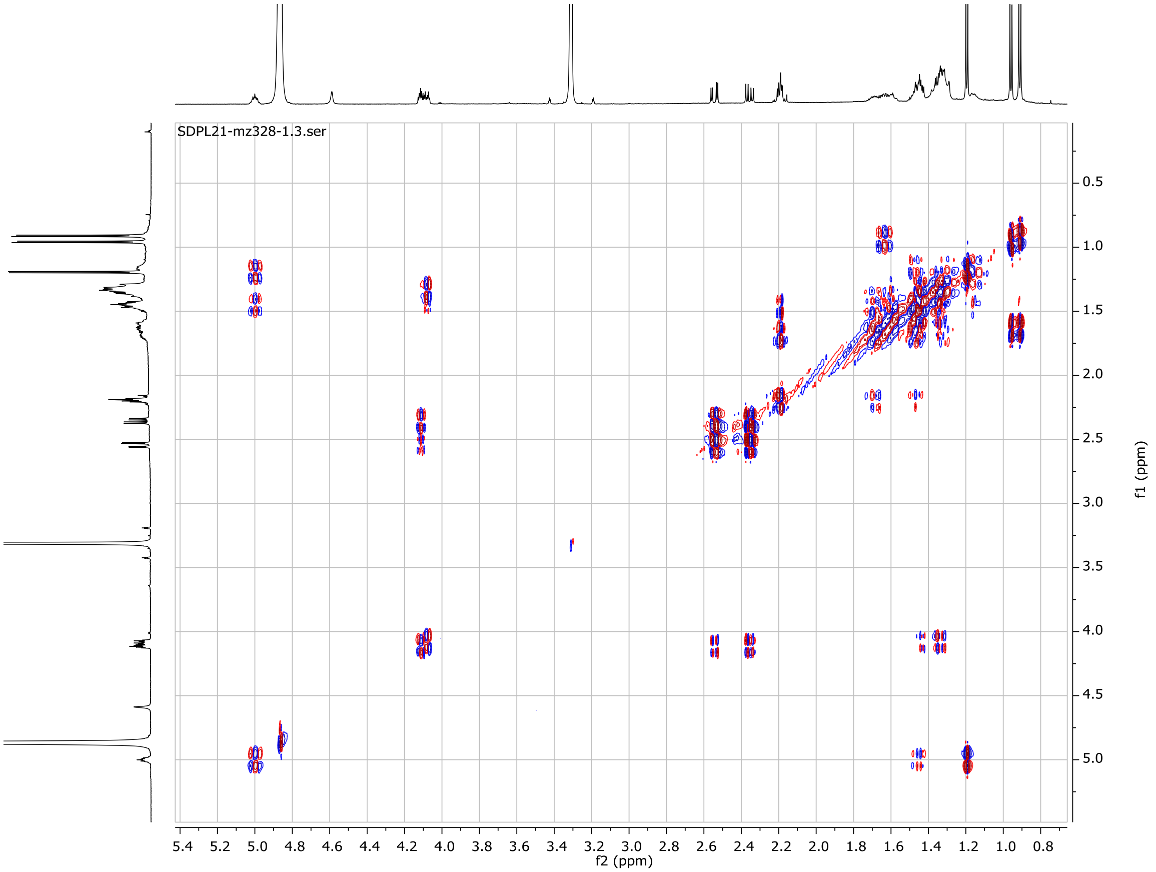

Supplementary Figure 40. DFT-COSY spectrum of 1 in CD3OD (600 MHz). DFT (density functional theory).

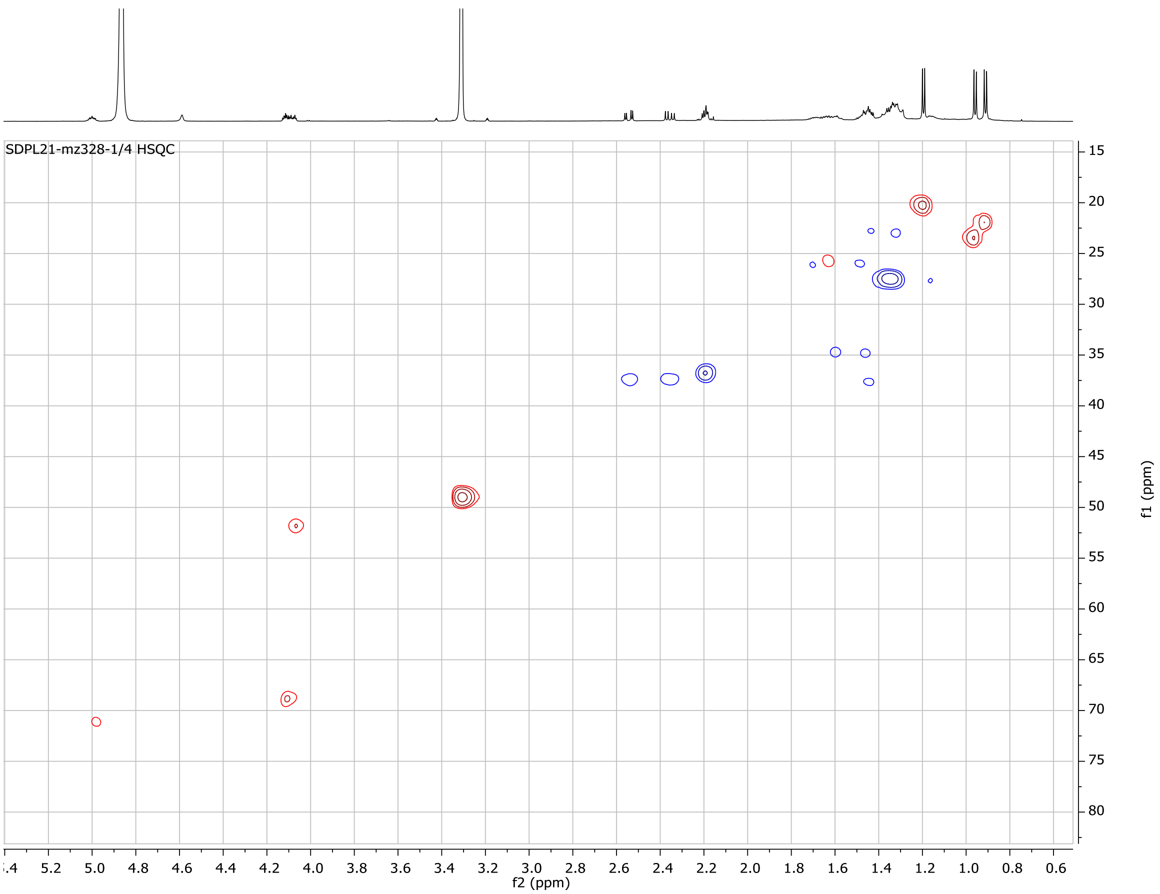

Supplementary Figure 41. HSQC spectrum of 1 CD_3OD (600 MHz).

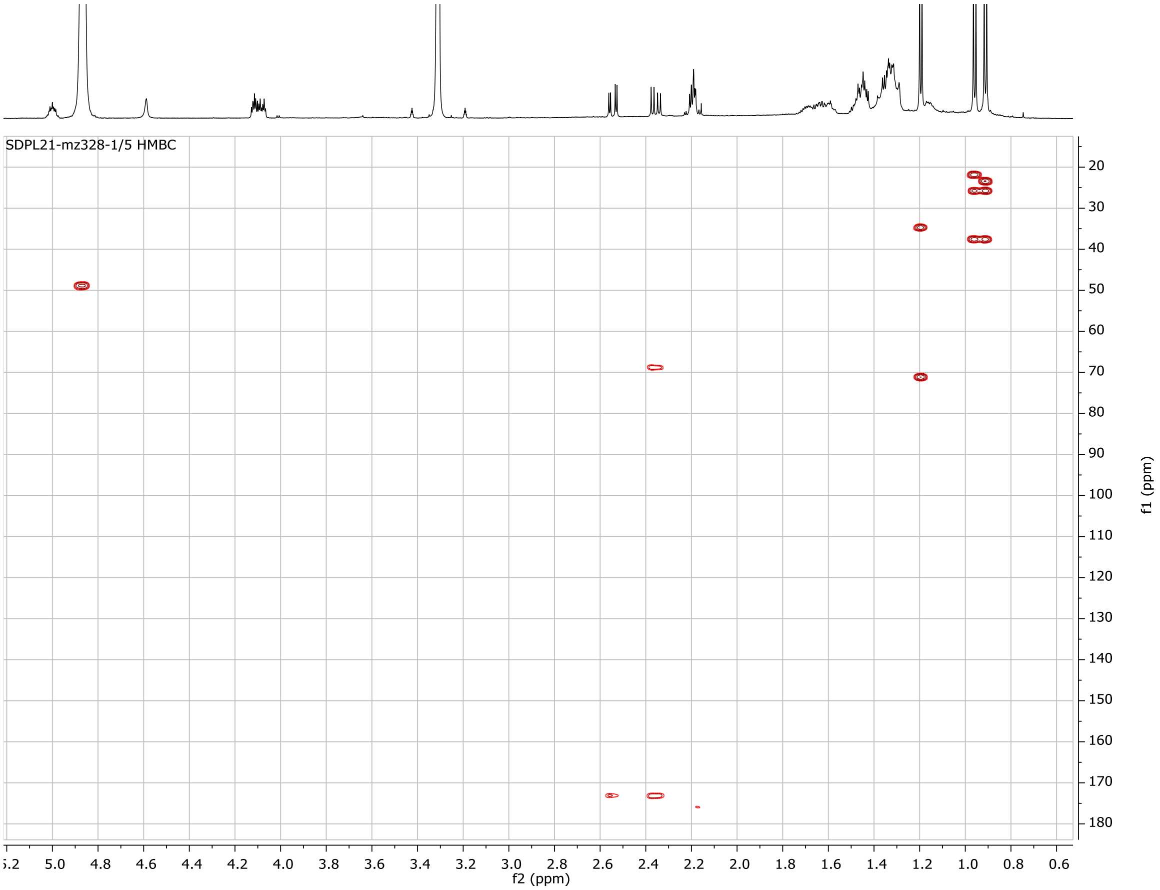

Supplementary Figure 42. HMBC spectrum of 1 in CD_3OD (600 MHz).

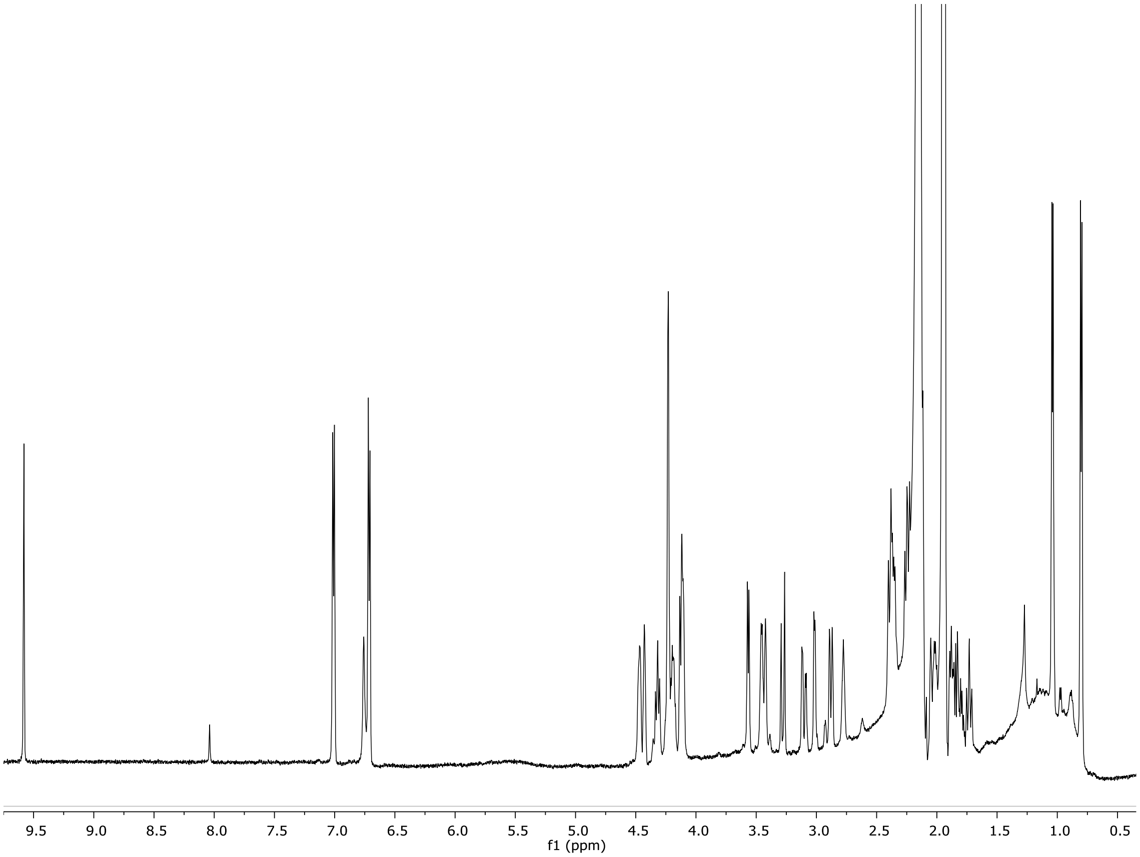

Supplementary Figure 43. ^{1}H NMR spectrum of cabrillospiral A (2) in CD_3CN (600 MHz).

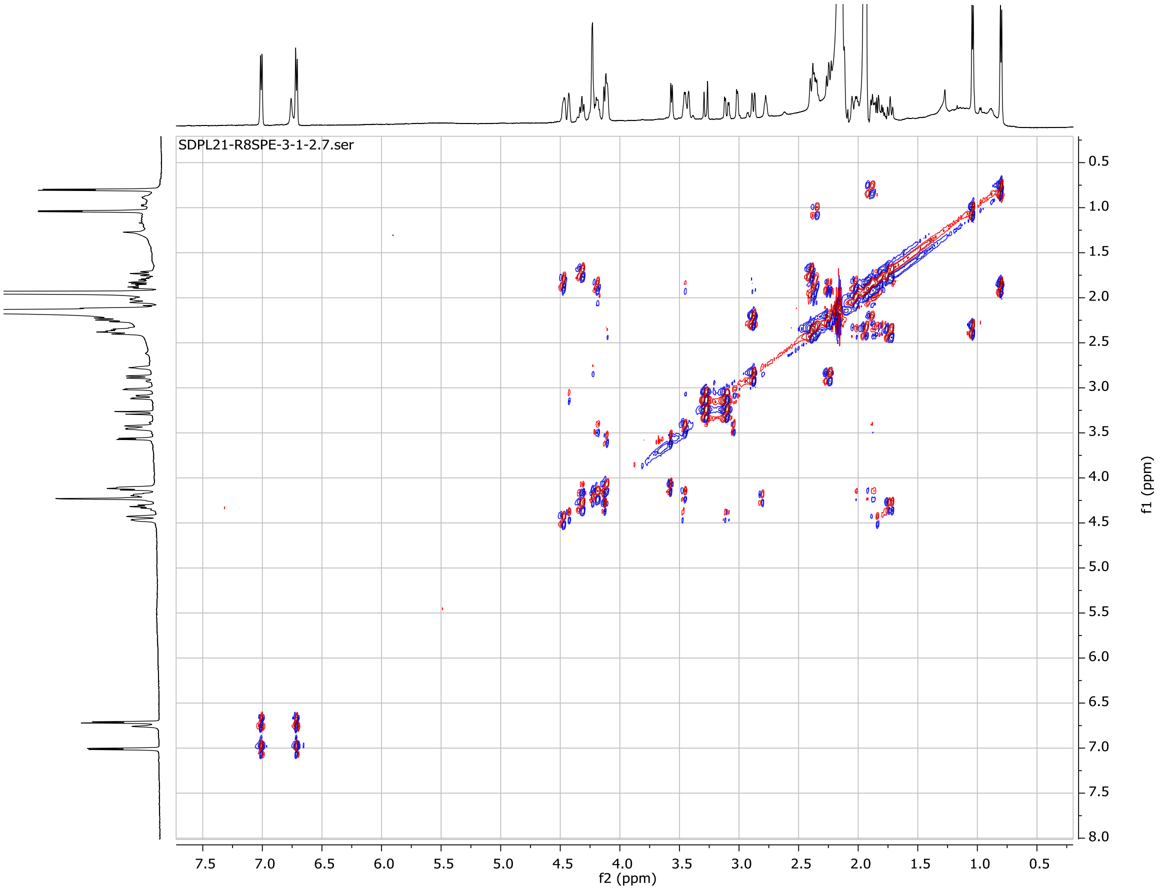

Supplementary Figure 44. COSY spectrum of 2 in CD_3CN (600 MHz).

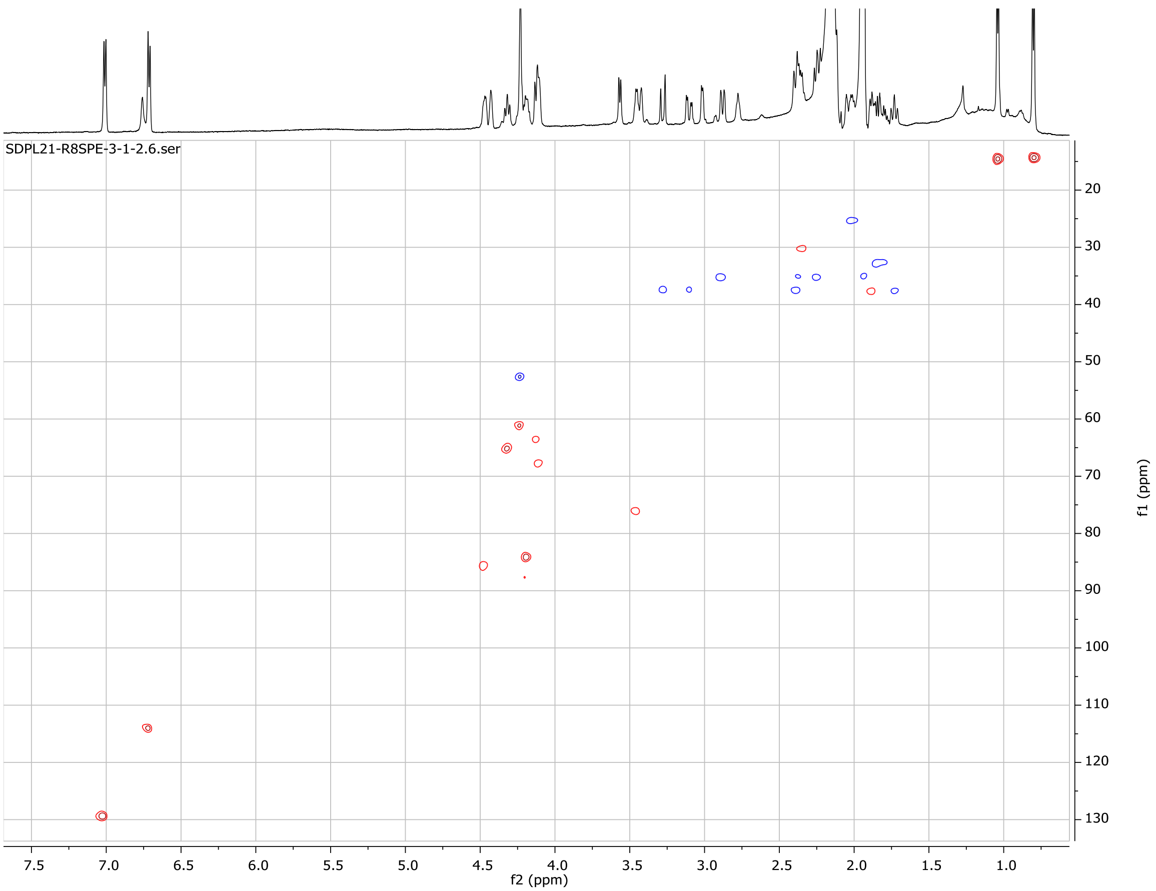

Supplementary Figure 45. HSQC spectrum of 2 in CD_3CN (600 MHz).

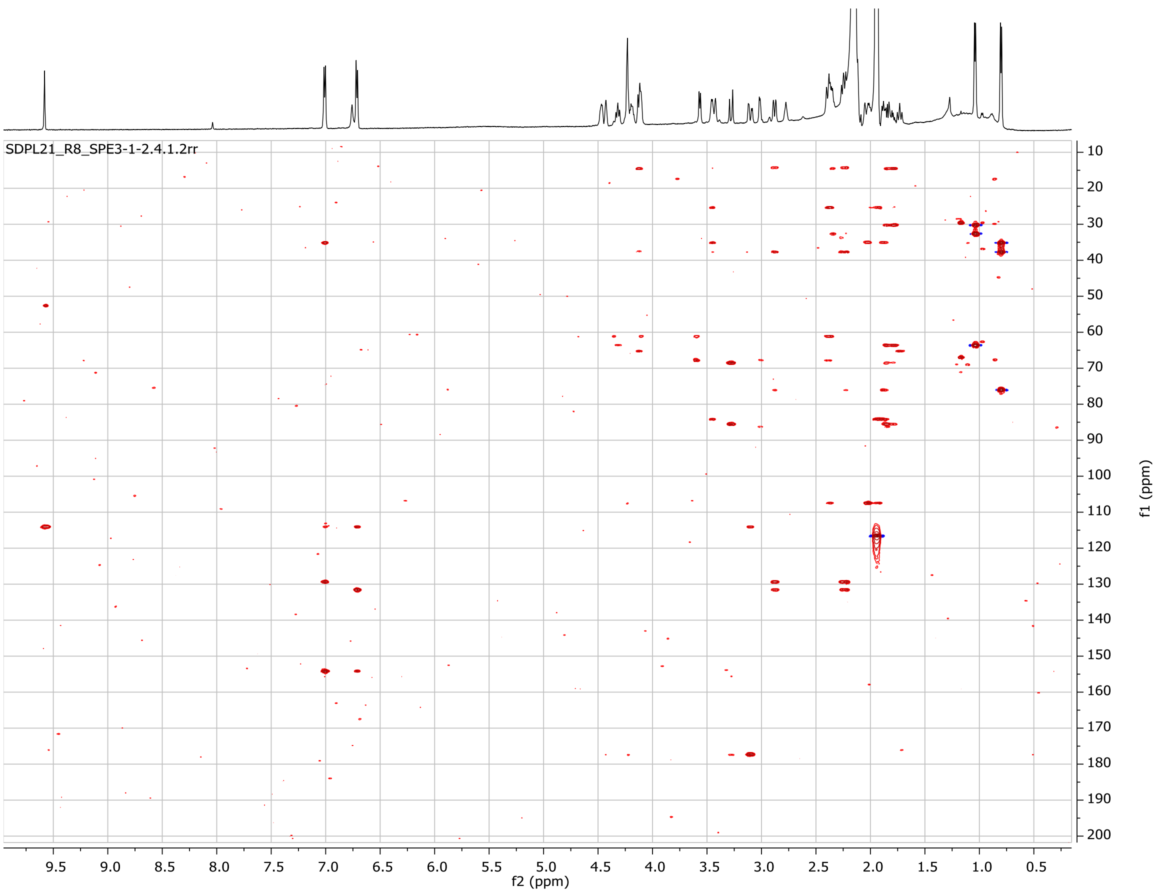

Supplementary Figure 46. HMBC spectrum of 2 in CD_3CN (600 MHz).

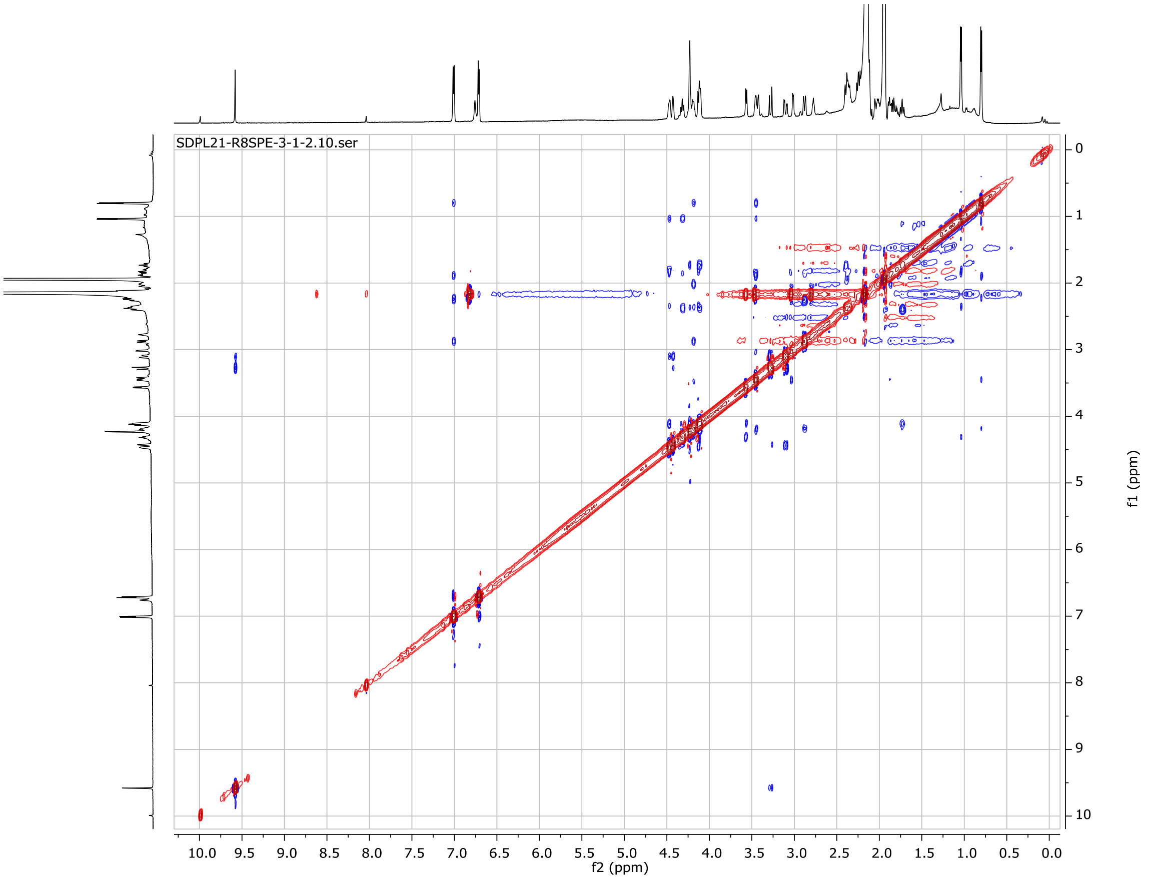

Supplementary Figure 47. NOESY spectrum of 2 in CD_3CN (600 MHz).

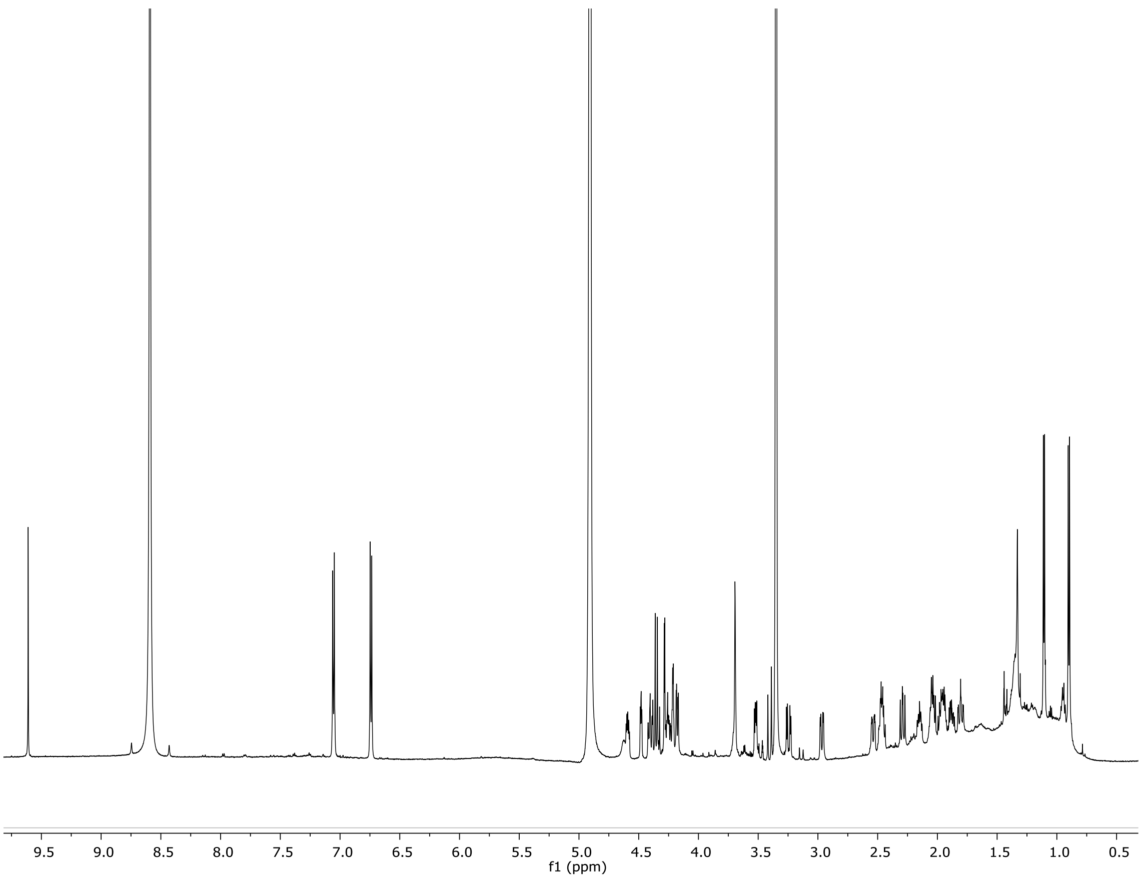

Supplementary Figure 48. ^1H NMR spectrum of 2 in CD_3OD (600 MHz).

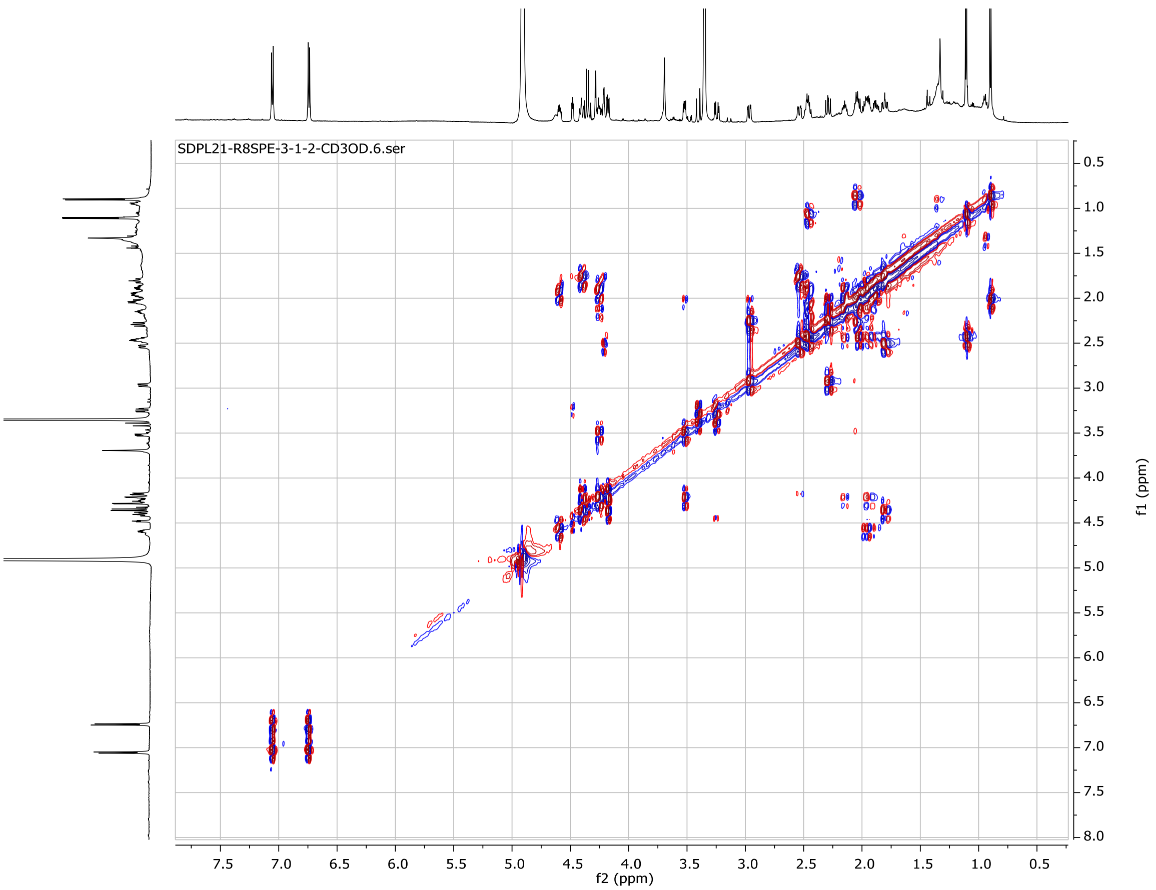

Supplementary Figure 49. COSY spectrum of 2 in CD_3OD (600 MHz).

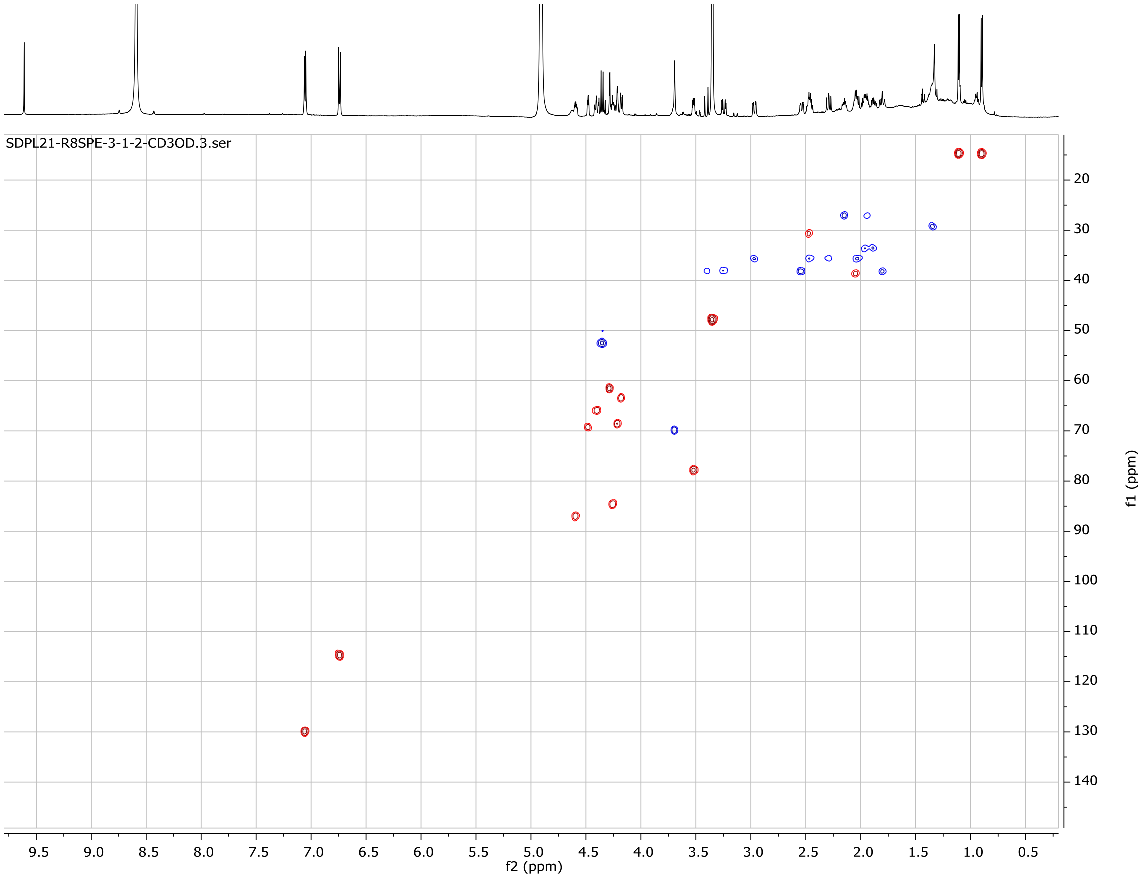

Supplementary Figure 50. HSQC spectrum of 2 in CD_3OD (600 MHz).

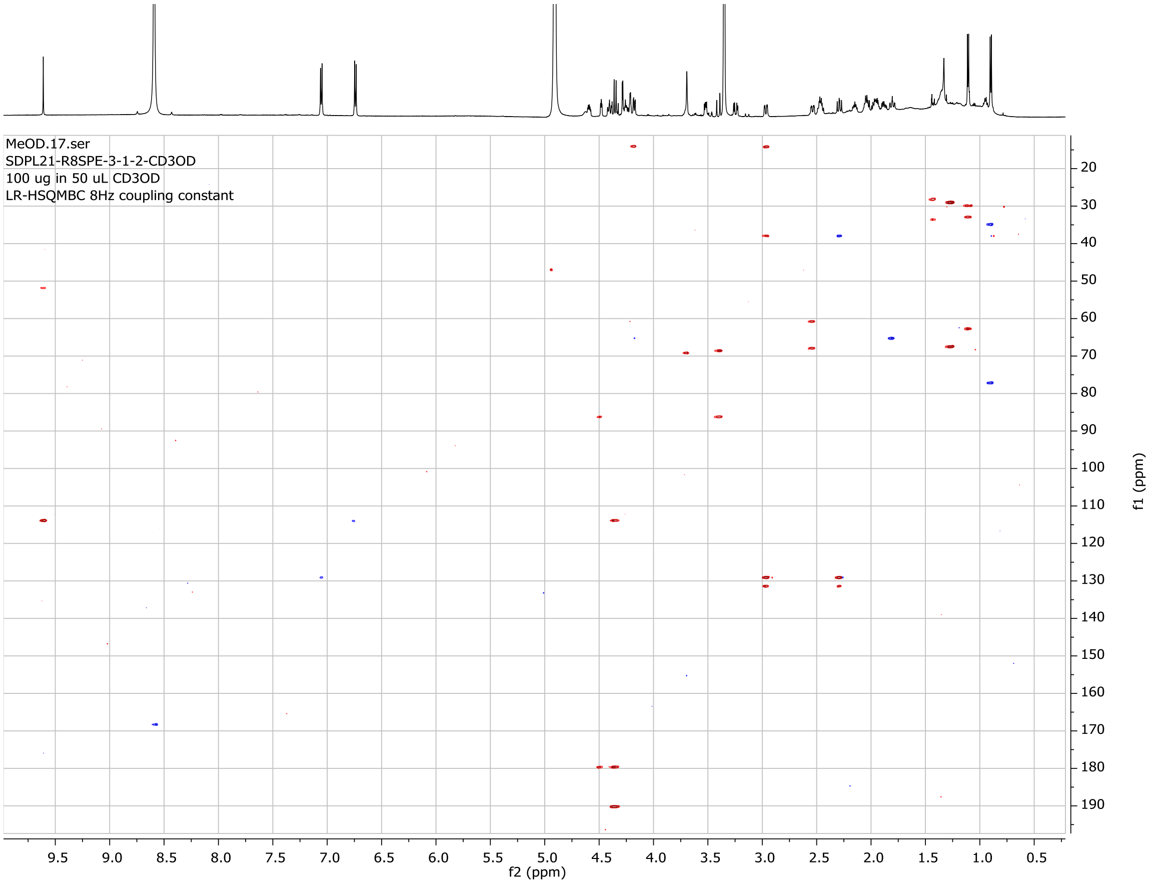

Supplementary Figure 51. LR-HSQMBC spectrum of 2 in CD_3OD (600 MHz).

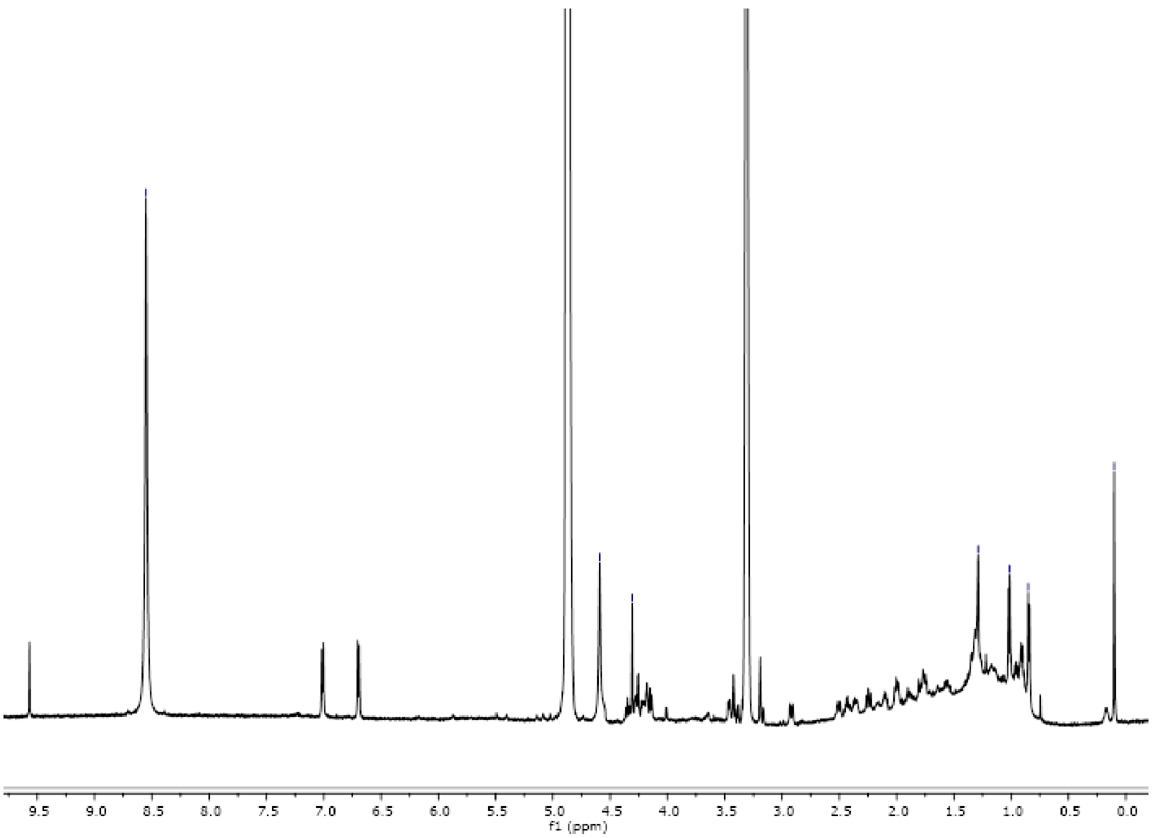

Supplementary Figure 52. ^{1}H NMR spectrum of cabrillospiral B (3) in CD_3OD (600 MHz).

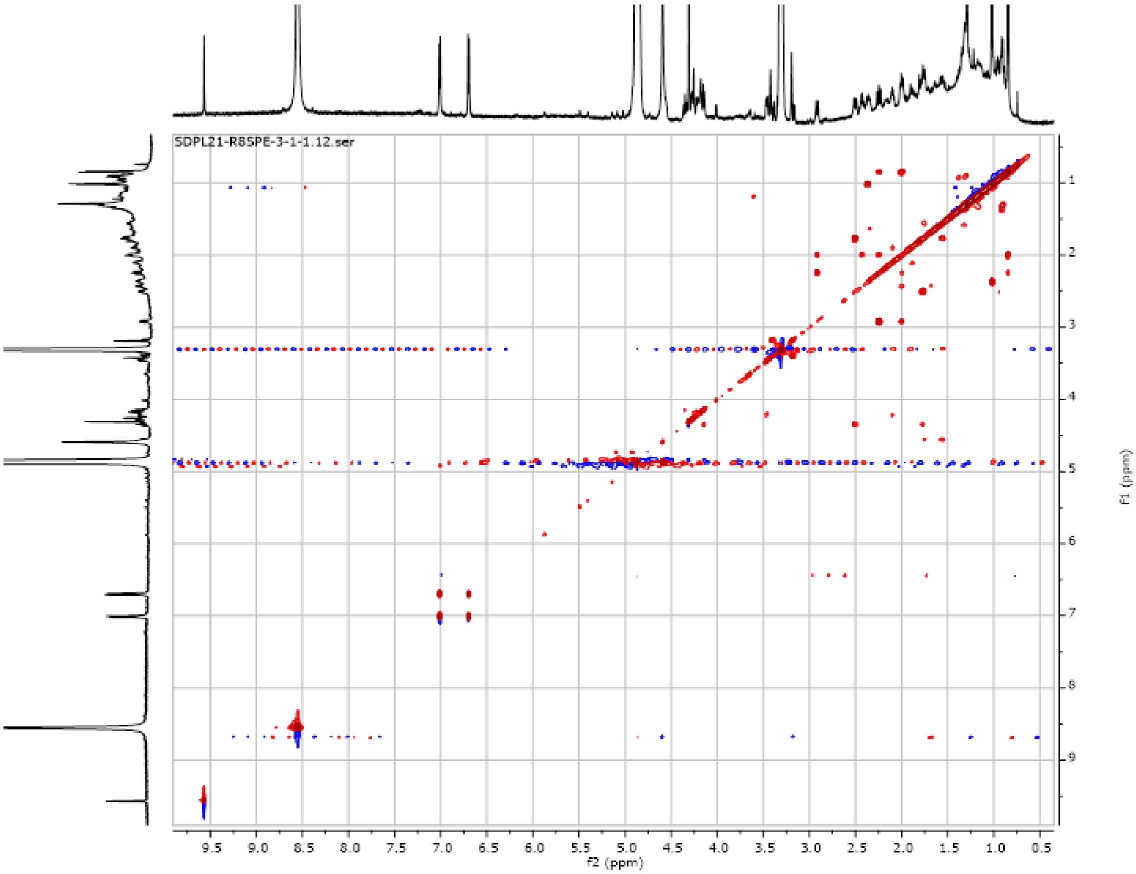

Supplementary Figure 53. COSY spectrum of 3 in CD_3OD (600 MHz).

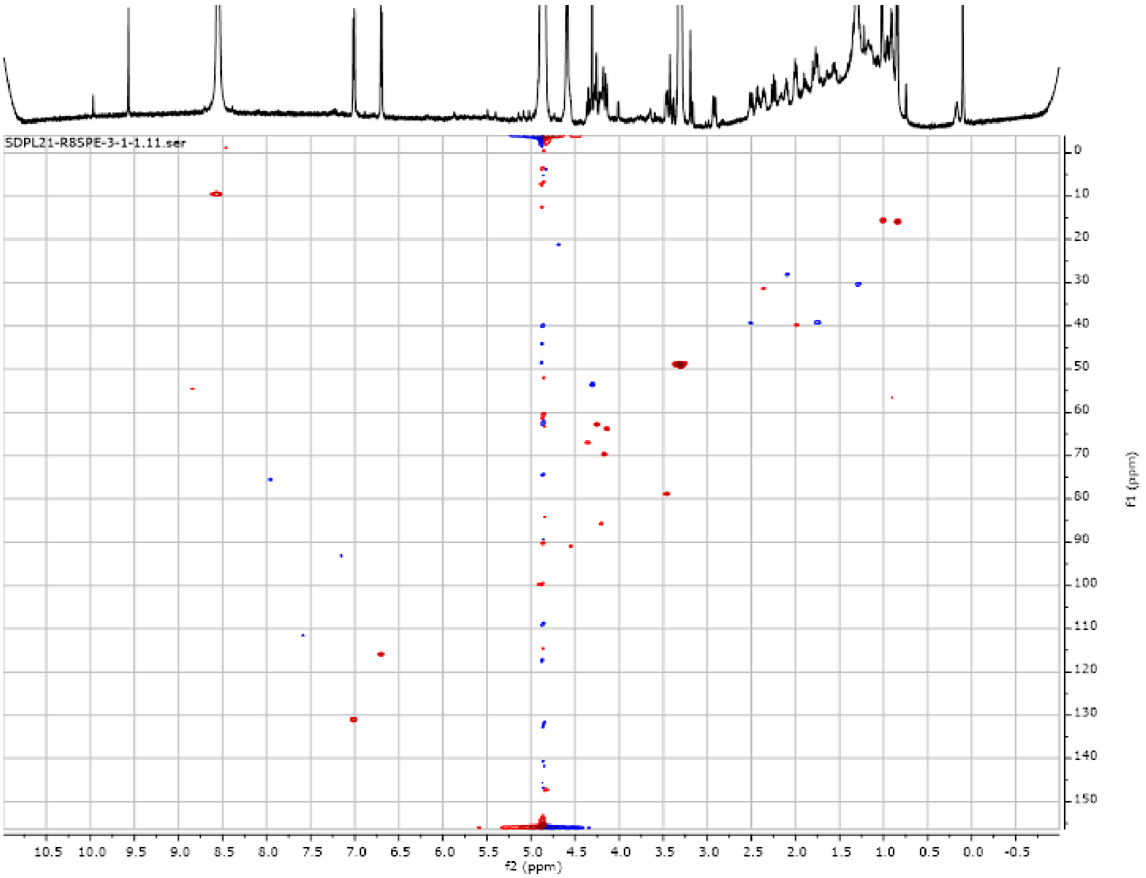

Supplementary Figure 54. HSQC spectrum of 3 in CD_3OD (600 MHz).

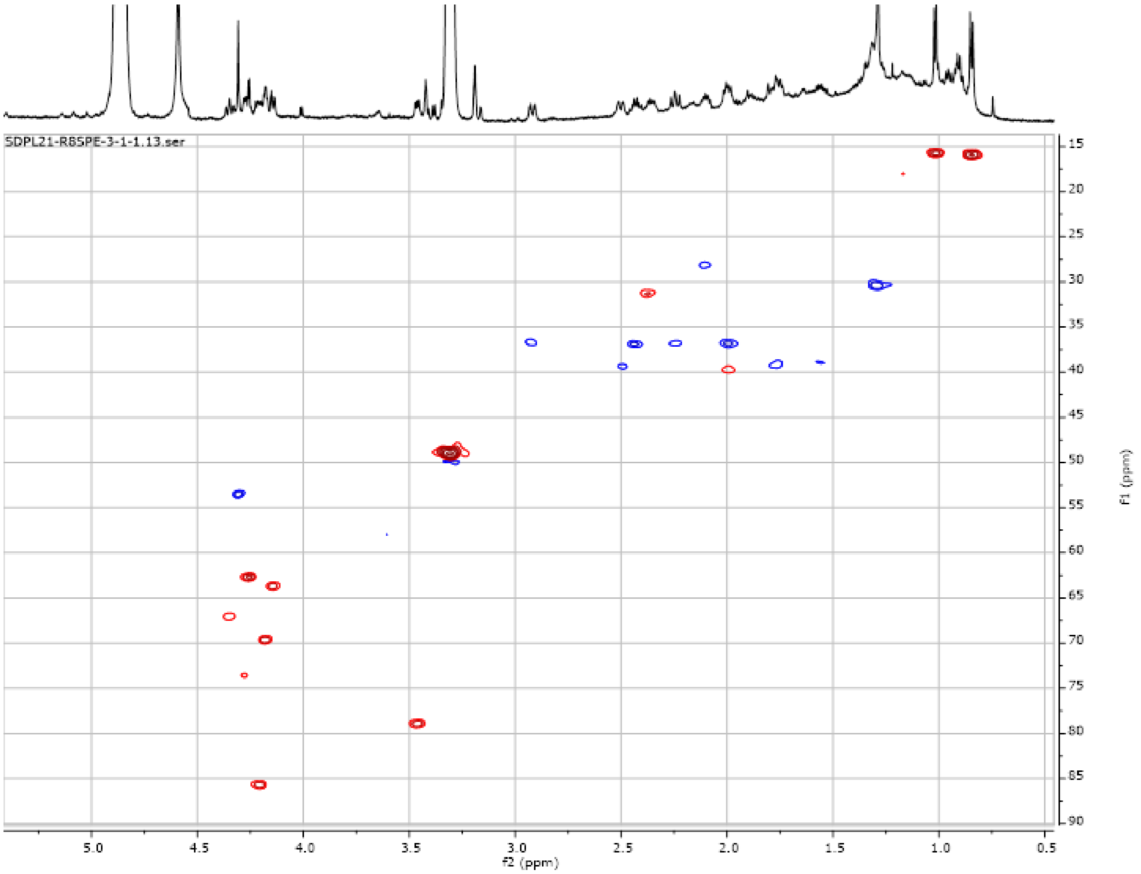

Supplementary Figure 55. HSQC spectrum of 3 in CD_3OD (600 MHz, upfield region).

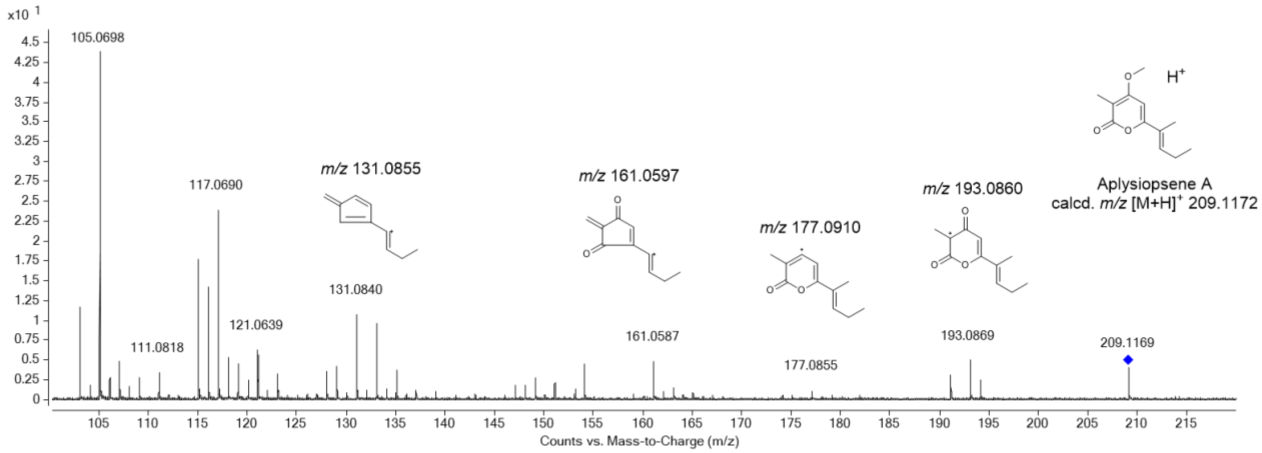

Supplementary Figure 56. Partially annotated ESI-MS/MS spectrum of aplysiopsene A.

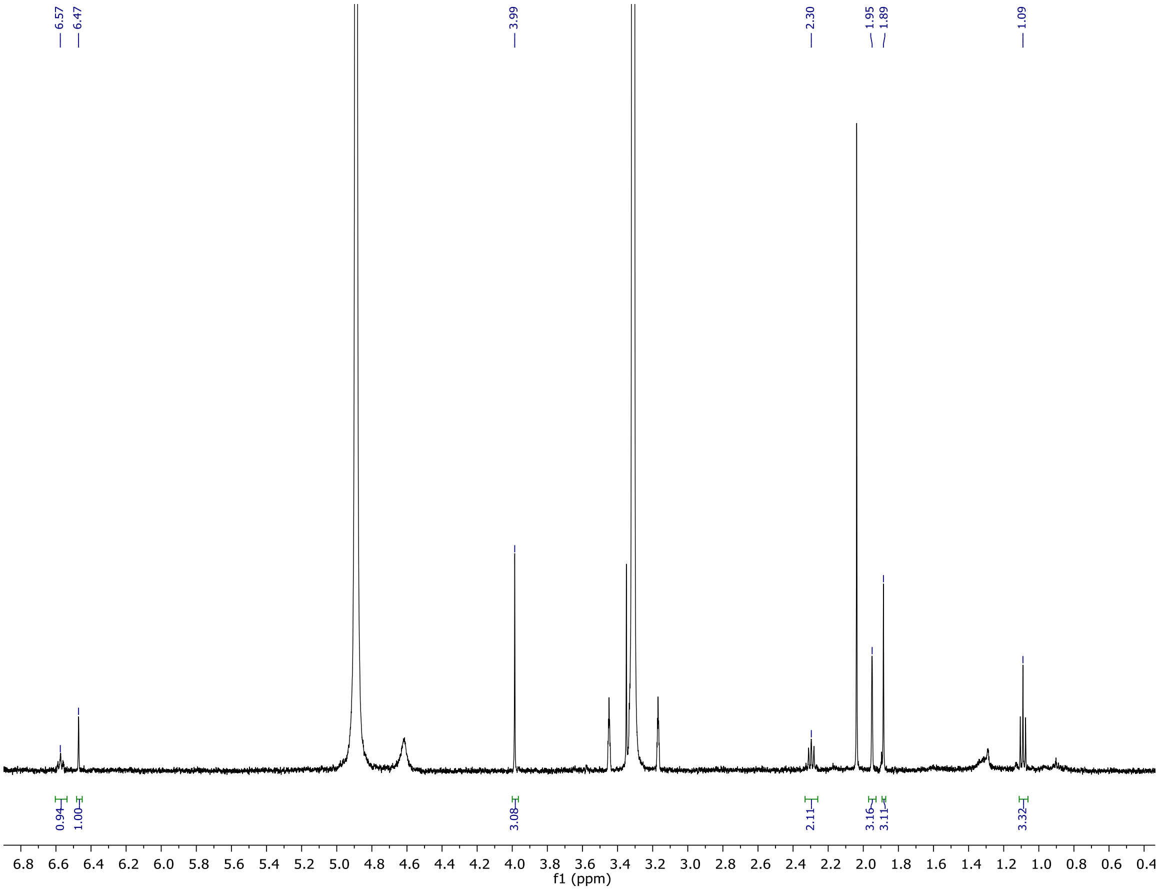

Supplementary Figure 57. ^{1}H NMR of aplysiopsene A in CD_3OD (500 MHz).

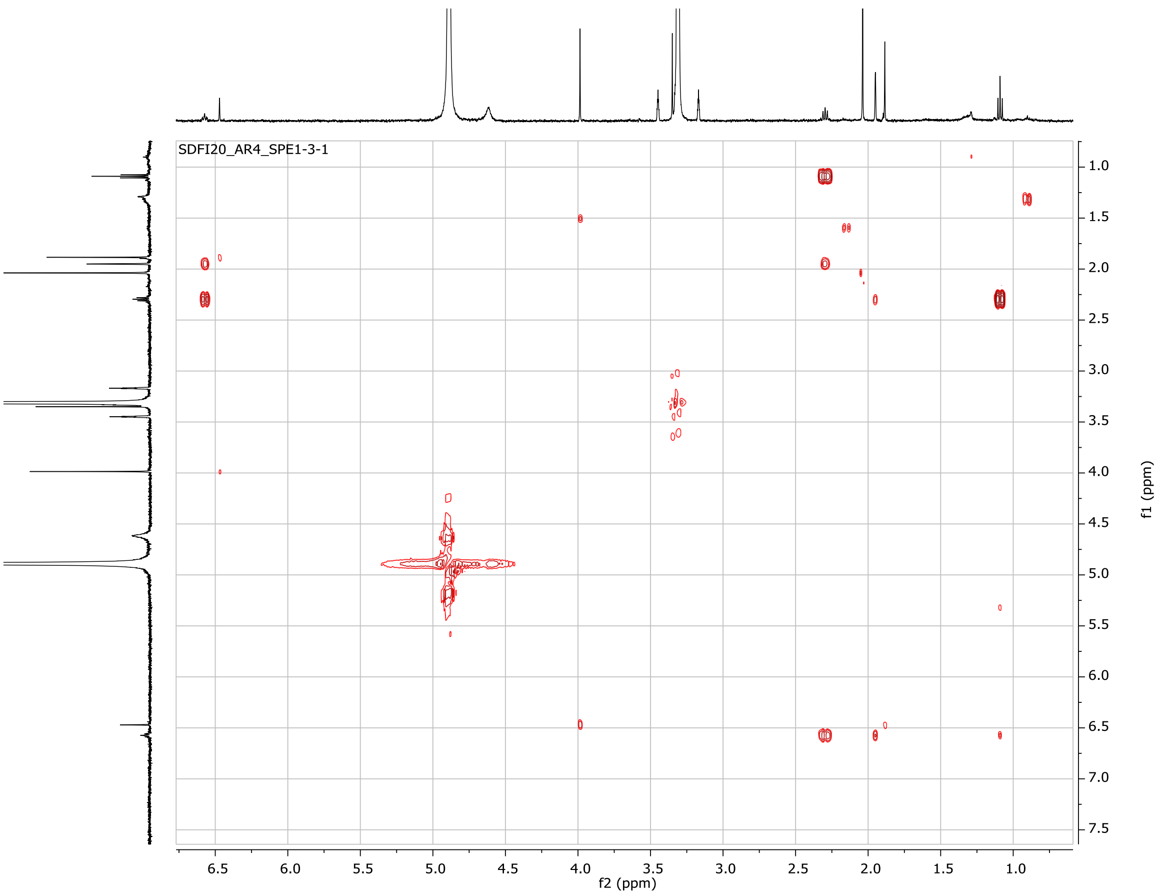

Supplementary Figure 58. COSY spectrum of aplysiopsene A in CD_3OD (500 MHz).

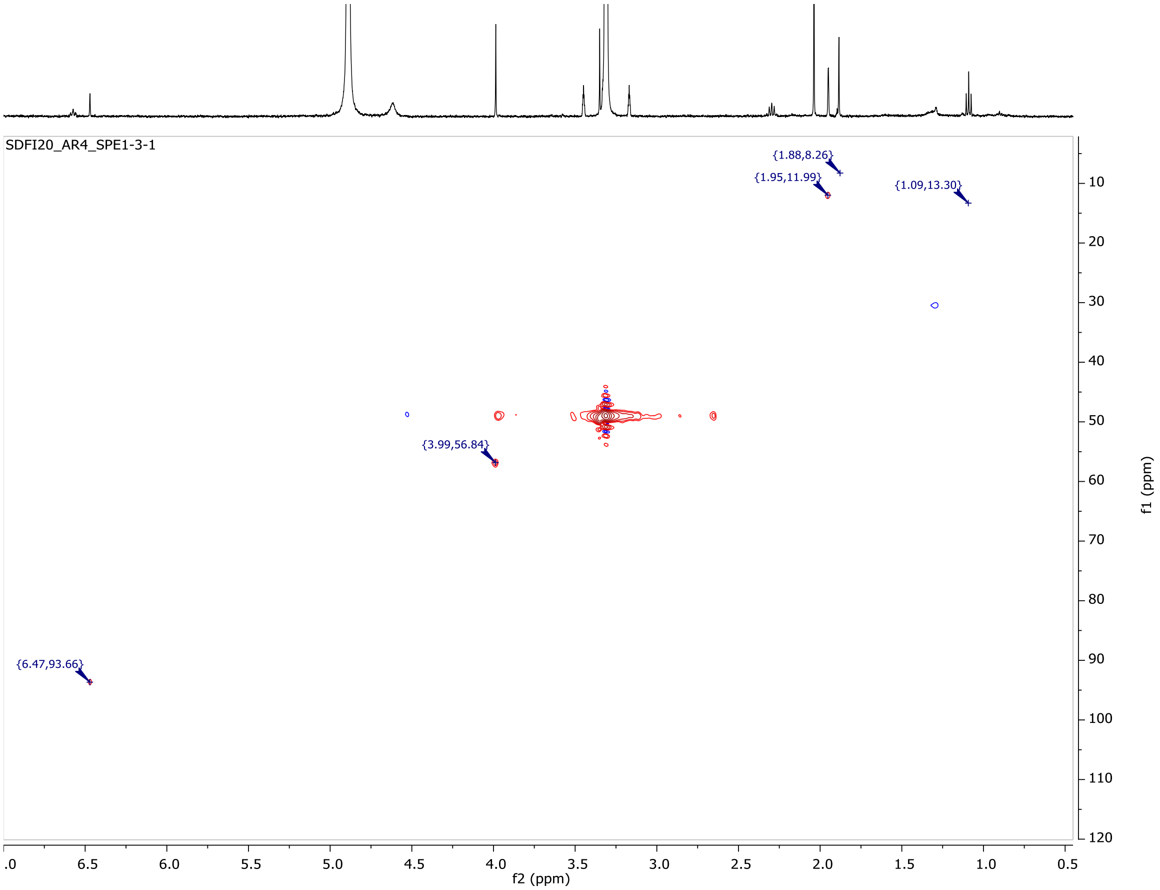

Supplementary Figure 59. HSQC spectrum of aplysiopsene A in CD_3OD (500 MHz).

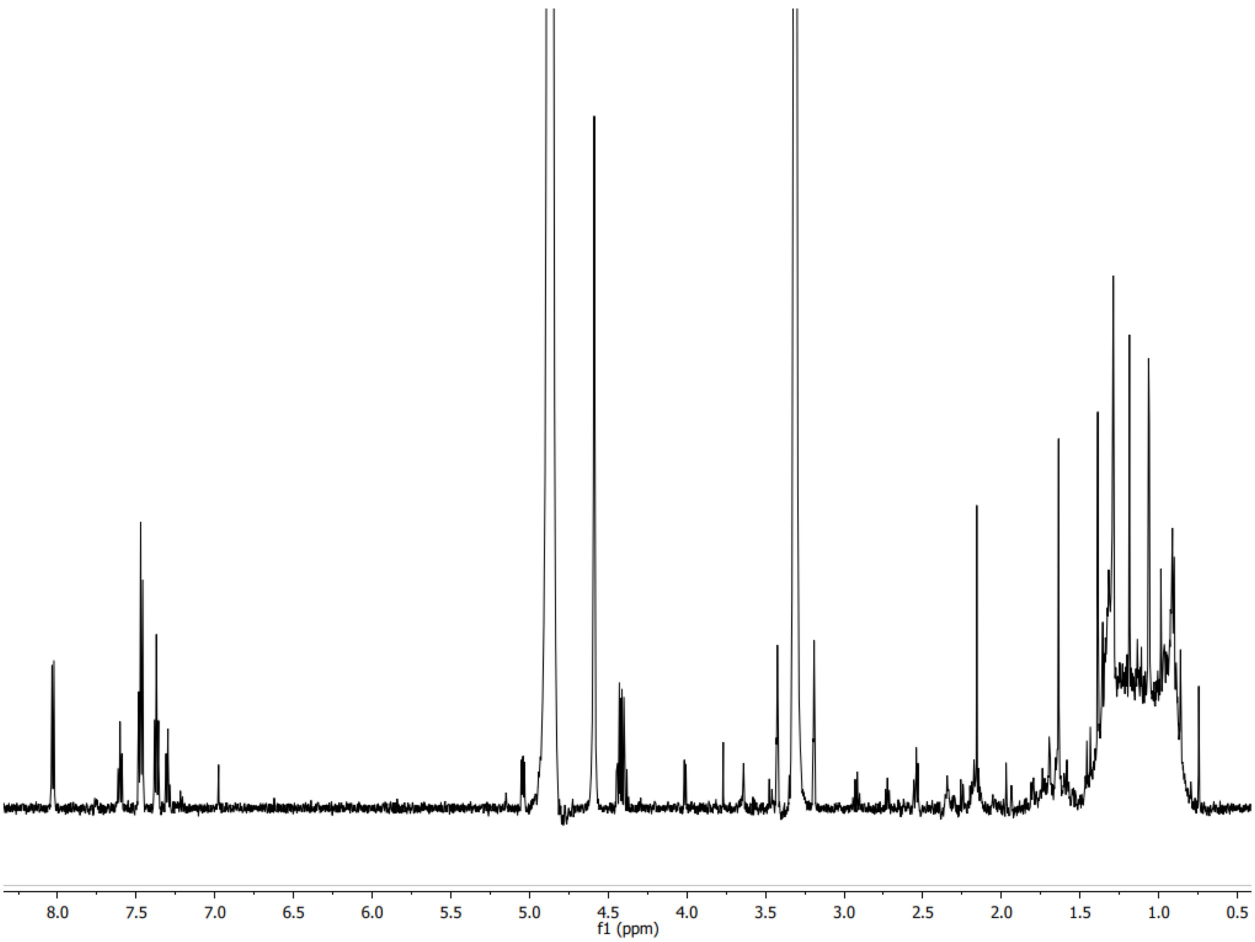

Supplementary Figure 60. ^{1}H NMR of the compound *m/z* 757 $[M+H]^{+}$ in CD_3OD (600 MHz).

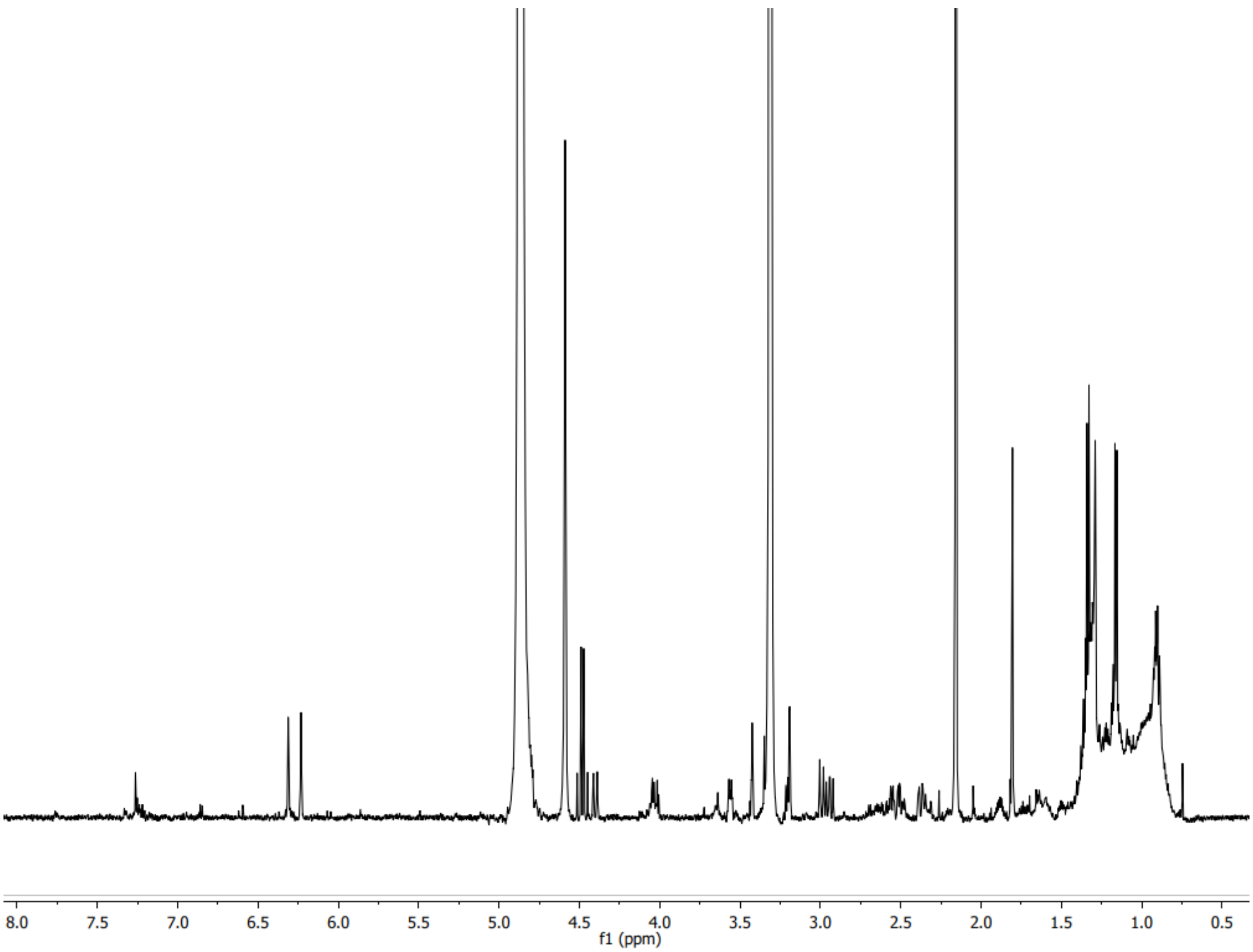

Supplementary Figure 61. ^{1}H NMR of the compound *m/z* 456 [M+Na]$^+$ in CD_3OD (600 MHz).

Supplementary Table 1. NMR data for cabrillostatin (1) in CD_3OD.

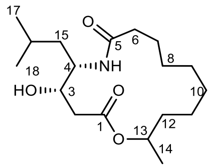

^{a}	δ_C ^{b}	δ_H (J in Hz)	COSY	HMBC
1	173.3, C			
2	37.5, CH_2	a 2.36, dd (7.7, 17.0)<br>b 2.54, dd (4.5, 17.0)	H-3<br>H-3	C-3, C-1<br>C-3 (w), C-1
3	68.9, CH	4.11, ddd (3.6, 4.5, 7.7)	H_2-2, H-4	
4	51.8, CH	4.08, dt (3.6, 10.4)	H-3, H_2-15	
5	175.7, C			
6	36.8, CH_2	a 2.18, m<br>b 2.20, m	H_2-7	C-5
7	26.1, CH_2	a 1.48, m<br>b 1.71, m	 H_2-6, H_2-8	
8	27.5, CH_2	ab 1.35, m	H_2-7,	
9	27.5, CH_2	ab 1.35, m	H-10b	
10	27.7, CH_2	a 1.16, m<br>b 1.35, m	 H_2-9	
11	22.9, CH_2	a 1.32, m<br>b 1.44, m	H_2-10<br>H_2-10	
12	34.9, CH_2	a 1.46, m<br>b 1.60, m	H-13	
13	71.2, CH	5.00, m	H-12a, H_3-14	
14	20.4, CH_3	1.20, d (6.3)	H-13	C-12, C-13
15	37.8, CH_2	a 1.35, m<br>b 1.45, m	H-4, H-16<br>H-4, H-16	
16	25.8, CH	1.64, m	H_3-17, H_3-18	
17	23.6, CH_3	0.96, d (6.7)	H-16	C-15, C-16, C-18
18	22.0, CH_3	0.90, d (6.5)	H-16	C-15, C-16, C-17

^{a} All assignments are based on extensive 1D and 2D NMR measurements (COSY, HSQC, HMBC).

^{b} Multiplicities determined by HSQC.

Supplementary Table 2. NMR data of cabrillospiral A (2) in CD_3CN.

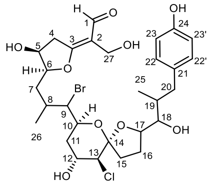

a	δC b	δH (J in Hz)	COSY	HMBC	NOESY
1	n. o., CHO	9.58, s		C-2, C-27	H_2-4
2	116.2, C				
3	179.4, C				
4	39.6, CH_2	a 3.10, dd (4.9, 17.5)<br>b 3.29, d (17.5)	H-5	C-2, C-3<br>C-3, C-5, C-6	H-5, H-6<br>H-5
5	70.5, CH	4.44, br s	H-4b	C-3 (weak)	H-4a, H-4b (weak), H-7a
6	87.7, CH	4.48, m	H-7a		H-8, H-9, H-4b, H_3-26
7	32.7, CH_2	a 1.79, m<br>b 1.85, m	 H-6	C-5, C-6, C-8, C-9<br>C-5, C-6, C-8, C-9	H-6, H-9
8	30.2, CH	2.35, m	H-9, H_3-26	C-7, C-9, C-10	
9	65.7, CH	4.13, m	H-10,	C-10, C-11, C-26	H-6
10	67.3, CH	4.32 br t (10.3)	H-9, H-11b	C-9 (weak)	H-11b, H_3-26
11	39.7, CH_2	a 1.73, br t (12.9)<br>b 2.39, br t (12.9)	H-10<br>H-12	 C-12	H-12, H-13
12	69.8, CH	4.11, m	H-11a	C-13	
12	OH	3.57 br d (7.5 Hz)	H-12	C-12, C-13	H-10, H-12
13	63.2, CH	4.23 m		C-12, C-14 (weak)	H-11a, H-15b
14	109.6, C				
15	37.1, CH_2	a 1.92, m<br>b 2.37, m	H_2-16	C-14, C-16, C-17<br>C-13, C-14, C-16	
16	27.3, CH_2	a 1.88, m<br>b 2.02, m	 H_2-15	C-15, C-17, C-18<br>C-14, C-15	
17	86.2, CH	4.20, m	H_2-16, H-18		H-15b, H-16b
18	78.3, CH	3.46 m	H-17, H-19	C-16, C-17, C-19, C-20	H-16a, H_3-25, H_3-26
19	39.8, CH	1.89, m	H-17, H_2-20, H_3-25	C-20	
20	37.3, CH_2	a 2.25 m<br>b 2.89 br d (13.0)	H-19<br>H-19	C-21, C-22/22′, C-19, C-25	
21	133.7, C				
22, 22′	131.4, CH	7.01, d (8.0)	H-23/23’	C-20, C-23/23′, C-24	
23, 23′	116.1, CH	6.72 d (8.0)	H-22/22’	C-21, C-22/22′, C-24	
24	154.2 C				
25	16.4, CH_3	0.80, d (6.8)	H-19	C-18, C-19, C-20	
26	16.6, CH_3	1.04, d (6.2)	H-8	C-7, C-8, C-9	
27	54.7, CH_2	4.24, br s		C-3 (weak)	

[a] All assignments are based on extensive 1D and 2D NMR measurements (COSY, HSQC, HMBC).
[b] Multiplicities determined by HSQC.

Supplementary Table 3. NMR data of cabrillospiral A (2) in CD_3OD.

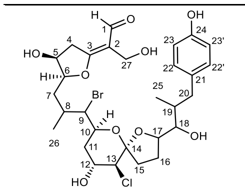

[a]	δ_C [b]	δ_H (J in Hz)	COSY	HMBC
1	192.1, C	9.57, s		C-2, C-27
2	115.8, C			
3	181.5, C			
4	39.3, CH_2	a 3.21, dd (4.9, 17.6)	H-5	
		b 3.36, d (17.6)		C-5, C-6
5	70.5, CH	4.43, dd (3.5, 4.9)	H-4b, H-6	C-3, C-6
6	88.2, CH	4.55, ddd (3.5, 4.5, 8.5)	H-5, H_2-7	
7	34.9, CH_2	a 1.85, m	H-6, H-8	
		b 1.92, m	H-6, H-8	C-9
8	31.8, CH	2.43, m	H-7, H-9, H-26	
9	64.6, CH	4.14, dd (2.2, 8.8)	H-8, H-10	C-10, C-11, C-26
10	67.2, CH	4.36 ddd (1.9, 8.8, 11.0)	H-9, H-11b	
11	39.4, CH_2	a 1.76, ddd (2.5, 11.0, 14.0)	H-10, H-12	C-10
		b 2.50, ddd (1.9, 3.6, 14.0)	H-12	C-12, C-13
12	69.8, CH	4.17, ddd (2.5, 3.2, 3.6)	H_2-11	C-10, C-13, C-14 (weak)
13	62.8, CH	4.24, d (3.2)		
14	109.3, C			
15	36.9, CH_2	a 1.99, m	H_2-16	
		b 2.43, m	H_2-16	
16	28.3, CH_2	a 1.90, m	H-15a, H-17	
		b 2.11, m	H-15a, H-17	
17	85.8, CH	4.21, m	H_2-16, H-18	C-16
18	79.0, CH	3.48, dd (4.0, 8.0)	H-17, H-18	C-16, C-17, C-20
19	39.9, CH	2.00, m	H-18, H_2-20, H_3-25	C-20
20	36.9, CH_2	a 2.24 dd (10.6, 13.3)	H-19	C-19, C-21, C-22/22’, C-25
		b 2.93 dd (3.5, 13.3)	H-19	C-19, C-21, C-22/22’, C-25
21	133.4, C			
22, 22′	131.1, CH	7.02, d (8.4)	H-23/23′	C-21, C-22/22’, C-24
23, 23′	115.9, CH	6.70 d (8.4)	H-22/22′	C-23/23′, C-24
24	156.2, C			
25	16.0, CH_3	0.86, d (6.7)		C-18, C-19, C-20
26	15.9, CH_3	1.07, d (6.5)		C-7, C-8, C-9
27	53.7, CH_2	a 4.30, d (11.3)		C-1, C-2, C-3
		b 4.33, d (11.3)		C-1, C-2, C-3

[a] All assignments are based on extensive 1D and 2D NMR measurements (COSY, HSQC, HMBC).

[b] Multiplicities determined by HSQC.

Supplementary Table 4. Comparison of ^{1}H and ^{13}C NMR data for cabrillospirals A (2) and B (3) in CD_3OD.

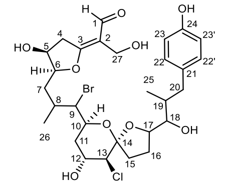

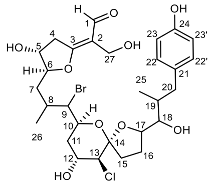

	Cabrillospiral A (**2**)		Cabrillospiral B (**3**)	
a	δ_C *b*	δ_H (*J* in Hz)	δ_C *b*	δ_H (*J* in Hz)
1	192.1, C	9.57, s	n.o.	9.57, s
2	115.8, C		n.o.	
3	181.5, C		n.o.	
4	39.3, CH_2	a 3.21, dd (4.9, 17.6)<br>b 3.36, d (17.6)	n.o.	n.o.
5	70.5, CH	4.43, dd (3.5, 4.9)	73.6, CH	4.28, dd (2.9, 5.9)
6	88.2, CH	4.55, ddd (3.5, 4.5, 8.5)	90.9, CH	4.55, m
7	34.9, CH_2	a 1.85, m<br>b 1.92, m	38.9, CH_2	a 1.55, m<br>b 1.76, m
8	31.8, CH	2.43, m	31.4, CH	2.38, m
9	64.6, CH	4.14, dd (2.2, 8.8)	63.7, CH	4.14, dd (2.1, 9.0)
10	67.2, CH	4.36 ddd (1.9, 8.8, 11.0)	67.1, CH	4.35, ddd (1.7, 9.0, 11.0)
11	39.4, CH_2	a 1.76, ddd (2.5, 11.0, 14.0)<br>b 2.50, ddd (1.9, 3.6, 14.0)	39.4, CH_2	a 1.78, m<br>b 2.50, br d (13.5)
12	69.8, CH	4.17, ddd (2.5, 3.2, 3.6)	69.7, CH	4.18, dd (3.2, 6.5)
13	62.8, CH	4.24, d (3.2)	62.7, CH	4.26, d (3.2)
14	109.3, C		n.o.	
15	36.9, CH_2	a 1.99, m<br>b 2.43, m	36.9, CH_2	a 1.99, m<br>b 2.43, m
16	28.3, CH_2	a 1.90, m<br>b 2.11, m	28.3, CH_2	a 1.89, m<br>b 2.11, m
17	85.8, CH	4.21, m	85.7, CH	4.21, m
18	79.0, CH	3.48, dd (4.0, 8.0)	79.0, CH	3.46, dd (4.0, 8.0)
19	39.9, CH	2.00, m	39.8, CH	2.00, m
20	36.9, CH_2	a 2.24 dd (10.6, 13.3)<br>b 2.93 dd (3.5, 13.3)	36.7, CH_2	a 2.24 dd (10.6, 13.3)<br>b 2.92 dd (3.5, 13.3)
21	133.4, C		n.o.	
22, 22′	131.1, CH	7.02, d (8.4)	131.1, CH	7.01, d (8.4)
23, 23′	115.9, CH	6.70 d (8.4)	115.9, CH	6.70 d (8.4)
24	156.2, C		n.o.	
25	16.0, CH_3	0.86, d (6.7)	15.9, CH_3	0.86, d (7.0)
26	15.9, CH_3	1.07, d (6.5)	15.7, CH_3	1.02, d (6.6)
27	53.7, CH_2	a 4.30, d (11.3)<br>b 4.33, d (11.3)	53.6, CH_2	ab 4.31, s

a All assignments are based on extensive 1D and 2D NMR measurements (COSY, HSQC, HMBC).

b Multiplicities determined by HSQC.

Supplementary Table 5. Cancer cell lines used for the high content screening.

Name	Cancer type	Mutation status	Reference compound classification accuracy
786-O	Kidney	VHL, p53, PTEN, and p16	0.942
A549	Lung	KRAS	0.974
AsPC1	Pancreas	KRAS	0.938
DU145	Prostate	p53	0.963
HCT116	Colon	KRAS	0.906667
HEPG2	Liver	p53	0.94
OVCAR4	Ovarian	TP53	0.9
U87MG	Brain	PTEN	0.966
WM164	Skin	BRAF	0.945

Supplementary Table 6. Reference compounds used for the high content screening.

Compound	Category	Targets	Pathway	Source well conc. in µM
Colchicine	MT	B-Tubulin	MT polymerization	5000
Docetaxel	MT	Microtubules	MT depolymerization	5000
Epothilone B	MT	Microtubules	MT depolymerization	5000
Nocodazole	MT	Î²-tubulin	MT polymerization	5000
Vinblastine	MT	Tubulin	MT polymerization	5000
AUY922	HSP90	HSP90Î±/Î²	HSP	5000
17-AAG	HSP90	Pan-HSP	HSP	5000
17-DMAG	HSP90	Pan-HSP	HSP	5000
Geldanamycin	HSP90	HSP90	HSP	5000
SNX2112	HSP90	HSP90Î±/Î²	HSP	5000
AZD8055	mTOR	mTOR	mTOR	5000
Deforolimus	mTOR	mTOR	mTOR	5000
INK128	mTOR	mTOR	mTOR	5000
Rapamycin	mTOR	mTOR	mTOR	5000
Torin 1	mTOR	mTORC1, mTORC2	mTOR	1000
Camptothecin	DNA	Topo I	Topoisomerases	5000
Eloxatin	DNA	DNA	DNA Synthesis	25000
Etoposide	DNA	Topo II	Topoisomerases	5000
Gemcitabine	DNA	Nucleic Acids	Nucleic Acid Synthesis	5000
Pemetrexed	DNA	DHFR, TS, GARFT	Nucleic Acid Synthesis	5000
Apicidin	HDAC	HDAC3	Epigenetic	5000
Oxamflatin	HDAC	HDAC	Epigenetic	5000
Panobinostat	HDAC	HDAC	Epigenetic	5000
SAHA	HDAC	HDAC	Epigenetic	5000
Aclacinomycin	Proteasome	Topo I, Topo II, 20S proteaome	Topoisomerases, Protein degradation	5000
Bortezomib	Proteasome	20S proteasome	Protein degradation	5000
MG-115	Proteasome	20S Proteasome, 26S Proteasome	Protein degradation	5000
MG132	Proteasome	proteasome, calpain	Protein degradation	5000

Supplementary Table 7. Results of bioactivity testing for 1-3 in the high content screening with nine cancer cell lines. Bioactivity was calculated as described in the Methods. P-values for compound-to-DMSO distances were calculated based on the empirical null distribution of DMSO-DMSO distances (one-sided, no adjustments for multiple comparisons).

Compound	Bioactive p value	Cell line	Compound	Bioactive p value	Cell Line
Cabrillospiral A	0.209294	U87MG	Cabrillospiral B	0.175548	A549
Cabrillospiral A	0.304065	U87MG	Cabrillostatin	1.55E-15	A549
Cabrillospiral A	0.1364	U87MG	Cabrillostatin	1.55E-15	A549
Cabrillospiral B	0.141661	U87MG	Cabrillostatin	1.55E-15	A549
Cabrillospiral B	0.045934	U87MG	Cabrillospiral A	0.434165	OVCAR4
Cabrillospiral B	0.004458	U87MG	Cabrillospiral A	0.671568	OVCAR4
Cabrillostatin	1.00E-16	U87MG	Cabrillospiral A	0.026876	OVCAR4
Cabrillostatin	1.00E-16	U87MG	Cabrillospiral B	0.721262	OVCAR4
Cabrillostatin	1.00E-16	U87MG	Cabrillospiral B	0.401609	OVCAR4
Cabrillospiral A	0.190758	AsPC1	Cabrillospiral B	0.5173	OVCAR4
Cabrillospiral A	0.362119	AsPC1	Cabrillostatin	3.28E-05	OVCAR4
Cabrillospiral A	0.256499	AsPC1	Cabrillostatin	1.28E-06	OVCAR4
Cabrillospiral B	0.39188	AsPC1	Cabrillostatin	0.000847	OVCAR4
Cabrillospiral B	0.263472	AsPC1	Cabrillospiral A	0.332002	HEPG2
Cabrillostatin	4.77E-15	AsPC1	Cabrillospiral A	0.376773	HEPG2
Cabrillostatin	4.77E-15	AsPC1	Cabrillospiral A	0.711658	HEPG2
Cabrillostatin	4.77E-15	AsPC1	Cabrillospiral B	0.194758	HEPG2
Cabrillospiral A	0.45305	WM164	Cabrillospiral B	0.399985	HEPG2
Cabrillospiral A	0.00022	WM164	Cabrillospiral B	0.64405	HEPG2
Cabrillospiral A	2.45E-05	WM164	Cabrillostatin	1.06E-08	HEPG2
Cabrillospiral B	5.57E-05	WM164	Cabrillostatin	0.039649	HEPG2
Cabrillospiral B	0.008047	WM164	Cabrillostatin	0.337498	HEPG2
Cabrillospiral B	2.47E-05	WM164	Cabrillospiral A	0.142646	786-O
Cabrillostatin	1.55E-15	WM164	Cabrillospiral A	0.191146	786-O
Cabrillostatin	1.55E-15	WM164	Cabrillospiral A	0.528463	786-O
Cabrillostatin	1.55E-15	WM164	Cabrillospiral B	0.014953	786-O
Cabrillospiral A	0.236295	DU145	Cabrillospiral B	0.285538	786-O
Cabrillospiral A	0.216205	DU145	Cabrillospiral B	0.260704	786-O
Cabrillospiral A	0.331292	DU145	Cabrillostatin	0.320406	786-O
Cabrillospiral B	0.422186	DU145	Cabrillostatin	0.007764	786-O
Cabrillospiral B	0.02437	DU145	Cabrillostatin	0.078936	786-O
Cabrillospiral B	0.594261	DU145	Cabrillospiral A	0.103913	HCT116
Cabrillostatin	1.00E-16	DU145	Cabrillospiral A	0.452732	HCT116
Cabrillostatin	6.68E-08	DU145	Cabrillospiral A	0.066401	HCT116
Cabrillostatin	4.41E-08	DU145	Cabrillospiral B	0.228838	HCT116

Cabrillospiral A	0.059789	A549	Cabrillospiral B	0.210873	HCT116
Cabrillospiral A	0.093785	A549	Cabrillospiral B	0.789221	HCT116
Cabrillospiral A	0.103537	A549	Cabrillostatin	3.35E-07	HCT116
Cabrillospiral B	0.550484	A549	Cabrillostatin	3.77E-12	HCT116
Cabrillospiral B	0.276498	A549	Cabrillostatin	5.08E-12	HCT116

Supplementary Table 8. Bioactivity profile for cabrillostatin (1) relative to reference compounds.

Cell line	Compound name	Predicted compound category	Confidence score
U87MG	Cabrillostatin	HDAC	0.097434563
U87MG	Cabrillostatin	HDAC	0.051281913
U87MG	Cabrillostatin	HDAC	0.065527331
AsPC1	Cabrillostatin	DMSO	0.057007311
AsPC1	Cabrillostatin	DMSO	0.068606556
AsPC1	Cabrillostatin	HDAC	0.099184119
WM164	Cabrillostatin	HDAC	0.003963972
WM164	Cabrillostatin	HDAC	0.029267674
WM164	Cabrillostatin	HDAC	0.016086107
DU145	Cabrillostatin	DMSO	0.004070139
DU145	Cabrillostatin	DMSO	0.002048989
DU145	Cabrillostatin	DMSO	0.003589487
A549	Cabrillostatin	HDAC	0.063783546
A549	Cabrillostatin	HDAC	0.060002645
A549	Cabrillostatin	HDAC	0.053045952
HCT116	Cabrillostatin	DMSO	0.021928432
HCT116	Cabrillostatin	HDAC	0.030559655
HCT116	Cabrillostatin	HDAC	0.025467136

Supplementary Table 9. Heart relevant drugs used as reference compounds in the IPSC-CM model.

Compound	Category	Targets	Pathway	Source well conc. µM
Metoprolol Tartrate	Neuronal Signaling	Adrenergic Receptor	Neuronal Signaling	10000
Dronedarone HCl	Transmembrane Transporters	Potassium Channel, Sodium Channel, Calcium Channel	Transmembrane Transporters	10000
Carvedilol	Neuronal Signaling	Adrenergic Receptor	Neuronal Signaling	10000
Amiodarone HCl	Transmembrane Transporters	Autophagy, Potassium Channel	Transmembrane Transporters	10000
Ibutilide Fumarate	Transmembrane Transporters	Sodium Channel	Transmembrane Transporters	10000
Diltiazem HCl	Transmembrane Transporters	Calcium Channel	Transmembrane Transporters	10000
Ajmaline	Cardiovascular Disease	Potassium Channel Sodium Channel	Cardiovascular Disease	10000
Atenolol	Neuronal Signaling	Adrenergic Receptor	Neuronal Signaling	10000
Adenosine 5'-monophosphate monohydrate	PI3K/Akt/mTOR	AMPK	PI3K/Akt/mTOR	10000
Sparteine	Others	Others	Others	10000
Adenosine disodium triphosphate	Cancer	Others	Cancer	10000
Entinostat (MS-275)	Epigenetics	HDAC	Epigenetics	10000
Nebivolol HCl	Neuronal Signaling	Adrenergic Receptor	Neuronal Signaling	10000
Bisoprolol fumarate	Neuronal Signaling	Adrenergic Receptor	Neuronal Signaling	10000
Lidocaine	Neuronal Signaling	Histamine Receptor	Neuronal Signaling	10000
Adenosine	GPCR & G Protein	Adenosine Receptor	GPCR & G Protein	10000
Dofetilide	Transmembrane Transporters	Potassium Channel	Transmembrane Transporters	10000
Lidocaine hydrochloride	Angiogenesis	EGFR	Angiogenesis	10000
Mexiletine HCl	Transmembrane Transporters	Sodium Channel	Transmembrane Transporters	10000
Digoxin	Transmembrane Transporters	Sodium Channel	Transmembrane Transporters	10000
Labetalol HCl	GPCR & G Protein	Adrenergic Receptor	GPCR & G Protein	10000

(R)-(+)-Atenolol HCl	Others	Adrenergic Receptor	Others	10000
Disopyramide Phosphate	Others	Others	Others	10000
Procainamide HCl	Transmembrane Transporters	DNA Methyltransferase, Sodium Channel	Transmembrane Transporters	10000
Verapamil HCl	Transmembrane Transporters	Calcium Channel	Transmembrane Transporters	10000
Propranolol HCl	GPCR & G Protein	Adrenergic Receptor	GPCR & G Protein	10000
Esmolol HCl	Neuronal Signaling	Adrenergic Receptor	Neuronal Signaling	10000
Timolol Maleate	Neuronal Signaling	Adrenergic Receptor	Neuronal Signaling	10000
Quinidine sulfate	Transmembrane Transporters	Sodium Channel	Transmembrane Transporters	10000
Propafenone HCl	Transmembrane Transporters	Sodium Channel	Transmembrane Transporters	10000
Sotalol HCl	Neuronal Signaling	Adrenergic Receptor	Neuronal Signaling	10000
Phenytoin Sodium	Transmembrane Transporters	Sodium Channel	Transmembrane Transporters	10000
Phenytoin	Transmembrane Transporters	Sodium Channel	Transmembrane Transporters	10000
Dronedarone	Others	Others	Others	10000
Carvedilol Phosphate	Others	Others	Others	10000
Esmolol	Neuronal Signaling	Adrenergic Receptor	Neuronal Signaling	10000
Nebivolol	Neuronal Signaling	Adrenergic Receptor	Neuronal Signaling	10000
(-)-Sparteine Sulfate	Transmembrane Transporters	Sodium Channel	Transmembrane Transporters	10000
Disopyramide	Transmembrane Transporters	Sodium Channel	Transmembrane Transporters	10000
Metoprolol	Neuronal Signaling	Adrenergic Receptor	Neuronal Signaling	10000

Supplementary Table 10. Compound annotations. A total of 5137 nodes were detected in the SMIRC extracts from four sites (Mission Bay, Cabrillo State Marine Reserve, Salton Sea, Scripps Pier). Of these, 325 (6.3% annotation rate) could be assigned to general compound classes (annotated) using GNPS classic molecular networking. After filtering redundant matches and duplicates, 230 unique compounds were identified. High cosine scores and low ppm *m/z* errors indicate the robustness of the library matches.

**Compound class**	**Number (%out of 230 matches)**	**Median cosine score**	**Median *m/z* error (ppm)**
Phosphatidylcholine lipids	41 (17.8%)	0.86	3.16
Fatty acids, amides, esters	27 (11.8 %)	0.82	5.8
Alkaloids and pseudoalkaloids	27 (11.8 %)	0.84	12.0
Terpenoids (diterpenoids, steroids, bile acids, etc.)	17 (7.4 %)	0.81	6.1
Flavonoids, lignans, coumarins	12 (5.2 %)	0.81	13.2
Peptides, amino acids	12 (5.2 %)	0.81	11.1
Others (incl. synthetic and non-classified)	94 (40.8 %)	0.83	8.3

Supplementary Table 11. MASST database matches for compounds captured using SMIRC.

Compound name or *m/z*	calcd. MF	Occurrence in SMIRC extracts	Number of MASST datasets hits, type of study
Cabrillostatin (**1**)	$C_{18}H_{33}NO_4$	all	11, DOM CA, deep water samples <500 m
Cabrillospirals **2**-**3**	$C_{29}H_{40}BrClO_9$	2021	none
Aplysiopsene A	$C_{12}H_{16}O_3$	2020-2023	2, Indonesian coral reef colonization experiments on ceramic plates, *Salinispora* sp.
757.463 $[M+H]^+$	$C_{42}H_{64}N_2O_{10}$	2021-2023	22, DOM, CA and tropical Pacific Ocean
727.438 $[M+H]^+$	$C_{41}H_{62}N_2O_9$	2021-2023	2, DOM, CA
743.431 $[M+H]^+$	$C_{41}H_{62}N_2O_{10}$	2021-2023	1, DOM, CA
769.464, $[M+H]^+$	$C_{43}H_{64}N_2O_{10}$	2021-2023	5, DOM, CA
785.462, $[M+H]^+$	$C_{43}H_{64}N_2O_{11}$	2021-2023	none
1031.560, $[M+H]^+$	$C_{54}H_{82}N_2O_{17}$	2021-2023	1, DOM, CA
1211.64, $[M+Na]^+$	$C_{63}H_{96}O_{21}$	2021-2023	none
836.4044 $[M+NH_4]^+$	$C_{42}H_{65}Cl_3O_9$	2022-2023	none
577.381, $[M+H]^+$	$C_{27}H_{52}N_4O_9$	2021-2023	7, DOM, CA
592.0393, $[M+H]^+$	$C_{26}H_{24}Cl_2BrN_3O_4$	2021	none
1086.6190, $[M+NH_4]^+$	$C_{52}H_{80}N_{10}O_{14}$	2021-2022	6, DOM, CA, South African Tunicate
456.1317 $[M+Na]^+$	$C_{20}H_{29}Cl_2NO_5$	2021-2023	3, DOM, CA
606.3572 $[M+H]^+$	$C_{34}H_{52}ClNO_6$	2022-2023	none
644.3936 $[M+H]^+$	$C_{34}H_{58}ClNO_8$	2022-2023	5, DOM CA, Coral reefs (Hawaii, Moorea)
488.257 $[M+H]^+$	$C_{23}H_{34}ClNO_8$	2021	none
913.4561 $[M+Na]^+$	$C_{47}H_{70}O_{16}$	2022-2023	none

Supplementary Table 12. Compounds detected in MASST datasets and corresponding MassIVE identifiers.

Compound name or *m/z*	MassIVE dataset identifiers
Cabrillostatin (**1**)	MSV000086926, MSV000087650, MSV000080176, MSV000088823, MSV000087735, MSV000087322, MSV000087588, MSV000084755, MSV000085852, MSV000085779
Aplysiopsene A	MSV000085159, MSV000078811
757.463 $[M+H]^+$	MSV000086926, MSV000084755, MSV000084741, MSV000084158, MSV000087006, MSV000082082, MSV000080015, MSV000080507, MSV000085480, MSV000088164, MSV000083632, MSV000082312, MSV000087650, MSV000085025, MSV000082952, MSV000088021, MSV000084744, MSV000083889, MSV000084119, MSV000086236, MSV000081731, MSV000085779
727.438 $[M+H]^+$	MSV000084741, MSV000084755
743.431 $[M+H]^+$	MSV000086926
769.464, $[M+H]^+$	MSV000086926, MSV000084755, MSV000087006, MSV000082312, MSV000084741
1031.560, $[M+H]^+$	MSV000084741
577.381, $[M+H]^+$	MSV000085480, MSV000084741, MSV000082312, MSV000085025, MSV000085786, MSV000087005
1086.6190, $[M+NH_4]^+$	MSV000086926, MSV000087006, MSV000085786, MSV000087449, MSV000083889, MSV000083888
456.1317 $[M+Na]^+$	MSV000082082, MSV000080015, MSV000085786
644.3936 $[M+H]^+$	MSV000084741, MSV000082084, MSV000082083, MSV000083889, MSV000085779

Supplementary Table 13. Cell painting dyes used for the phenotypic screening.

Material	Stock concentration	Final concentration [μl/ml]	Excitation wavelength [nm]
Phalloidin	2 units	0.5	568
Concanavalin A	5 mg/mL	20	488
SYTO 14 green	5 mM	0.6	490-515
Hoechst	10 mg/mL	0.5	385
Wheat-germ agglutinin	1 mg/mL	1.5	555
Mitotracker deep red	5 μM	0.5 μM	647

Supplementary Table 14. Cartesian coordinates for the minimal energy conformer of the model compound 4a.

H	-0.51794	-1.30903	-2.43251
C	-0.1071	-0.34098	-2.11972
C	-1.00943	0.31089	-1.06454
C	-0.51631	-0.38935	0.2097
O	0.9003	-0.56824	-0.02533
C	1.17615	-0.54902	-1.34511
H	-0.00186	0.29136	-3.00212
H	-2.06944	0.12149	-1.24125
C	2.44629	-0.6797	-1.78504
C	2.72098	-0.6719	-3.21322
H	1.85005	-0.60368	-3.89632
O	3.84676	-0.74426	-3.6846
O	-0.87174	1.71173	-1.02908
H	0.05701	1.92846	-0.86964
C	3.63201	-0.85335	-0.85848
H	4.00642	-1.88429	-0.95853
H	3.32039	-0.71679	0.17831
O	4.65392	0.08426	-1.1097
H	4.91415	-0.06455	-2.0316
C	-0.69965	0.35593	1.51782
H	-0.14954	1.30164	1.45854
H	-0.22952	-0.23202	2.31651
C	-2.16533	0.64612	1.87443
H	-2.5873	1.26011	1.06839
C	-2.22713	1.46639	3.16522
H	-1.66852	2.40318	3.06569
H	-1.80036	0.9077	4.00732
H	-3.26236	1.71671	3.41957
C	-3.00316	-0.63038	2.00115
H	-3.08146	-1.17463	1.0532
H	-4.02256	-0.39097	2.32095
H	-2.57181	-1.31215	2.74492
H	-0.96192	-1.39067	0.26508

Supplementary Table 15. Cartesian coordinates for the minimal energy conformer of the model compound 4b.

H	-0.48168	-1.57365	-2.54411
C	-0.48291	-1.67712	-1.46961
C	0.60139	-0.82971	-0.80094
O	0.06659	-0.57513	0.50198
C	-1.26872	-0.69358	0.54064
C	-1.75832	-1.12626	-0.82762
H	-2.1331	-0.26828	-1.37638
H	-2.53558	-1.87507	-0.79642
C	-1.95307	-0.45583	1.66255
C	-3.40902	-0.59657	1.67015
H	-3.89154	-0.87819	0.73193
O	-4.08284	-0.41428	2.64482
C	-1.30941	-0.03628	2.96705
H	-0.23702	-0.00658	2.8584
H	-1.64716	0.96423	3.22558
O	-1.58722	-0.92762	4.01005
H	-2.52052	-0.8949	4.17737
C	0.91822	0.47494	-1.52259
H	-0.01189	0.99888	-1.73211
H	1.35313	0.21888	-2.48713
C	1.86291	1.4288	-0.7738
H	1.4109	1.65643	0.18692
C	2.00045	2.73883	-1.55559
H	2.62645	3.44584	-1.0207
H	1.03457	3.20869	-1.7159
H	2.45284	2.56836	-2.52937
C	3.2407	0.81011	-0.51639
H	3.89543	1.52836	-0.03356
H	3.71441	0.50679	-1.44715
H	3.18489	-0.05814	0.13112
H	1.49565	-1.41649	-0.65558
O	-0.31562	-3.04344	-1.21796
H	-0.23287	-3.20204	-0.28568

Supplementary Table 16. Cartesian coordinates for the minimal energy conformer of the model compound 4c.

H	-0.066	-1.26787	-2.7347
C	-0.14796	-1.36253	-1.6512
C	0.98773	-0.62571	-0.92965
C	0.34636	-0.3863	0.44444
O	-1.04898	-0.15885	0.13454
C	-1.37205	-0.69979	-1.05757
H	-0.13711	-2.42875	-1.39276
H	0.39848	-1.31164	1.03212
C	-2.62284	-0.57886	-1.55269
C	-2.95785	-1.18992	-2.82927
H	-2.16357	-1.77414	-3.33701
O	-4.05832	-1.09529	-3.35329
C	0.86282	0.79169	1.24793
H	0.68342	1.70808	0.67499
H	0.2646	0.87012	2.16482
C	2.35467	0.70112	1.60273
H	2.91766	0.6672	0.66115
C	2.78171	1.96286	2.35685
H	2.58169	2.86358	1.76665
H	3.85281	1.93891	2.58373
H	2.23983	2.05461	3.30624
C	2.69013	-0.5532	2.41653
H	3.74489	-0.55082	2.71043
H	2.51312	-1.47659	1.8536
H	2.08829	-0.59777	3.333
C	-3.73385	0.15894	-0.83375
H	-3.33576	0.6724	0.04297
H	-4.47281	-0.57672	-0.47948
O	-4.34263	1.13843	-1.64436
H	-4.70132	0.65443	-2.40364
H	1.89567	-1.22562	-0.84923
O	1.3683	0.55748	-1.59106
H	0.58887	1.12054	-1.69307

Supplementary Table 17. Cartesian coordinates for the minimal energy conformer of the model compound 4d.

H	-0.83646	0.09099	-3.14188
C	-0.75975	0.31964	-2.08943
C	-1.84204	-0.40348	-1.28443
C	-1.17355	-0.5544	0.08352
O	0.21359	-0.69596	-0.23927
C	0.51469	-0.18762	-1.44323
H	-0.83514	1.39539	-1.96772
C	1.76848	-0.19196	-1.90317
C	2.06145	0.36891	-3.22198
H	1.22759	0.79601	-3.78292
O	3.15993	0.38316	-3.70195
C	2.94492	-0.75181	-1.13207
H	2.60872	-1.17544	-0.19921
H	3.63716	0.05455	-0.90294
O	3.6019	-1.77315	-1.82833
H	3.98193	-1.39219	-2.60972
C	-1.39436	0.6216	1.02774
H	-2.4562	0.65663	1.26508
H	-1.16434	1.54596	0.50224
C	-0.58102	0.58074	2.3315
H	0.47052	0.53352	2.0648
C	-0.90899	-0.64424	3.19125
H	-0.64871	-1.57334	2.69599
H	-1.96773	-0.67841	3.43744
H	-0.35576	-0.61259	4.12431
C	-0.81167	1.87056	3.12489
H	-0.54994	2.74798	2.54092
H	-0.20949	1.88355	4.02769
H	-1.85315	1.96723	3.42144
H	-1.47656	-1.48162	0.54569
H	-2.76357	0.15629	-1.2325
O	-2.17808	-1.63972	-1.84739
H	-1.42439	-2.21679	-1.85634

Supplementary Table 18. Coordinates for the dominant conformer of (12*R*,13*S*,14*S*)-5.

O	1.286	-0.084	-0.407
C	1.439	1.219	1.647
C	-0.304	-0.580	1.366
C	0.041	0.750	2.064
C	-0.027	-0.508	-0.148
C	1.598	1.202	0.132
H	2.192	0.565	2.100
H	0.960	1.960	-0.337
H	1.602	2.232	2.028
H	-1.352	-0.831	1.531
H	0.009	0.599	3.146
H	2.632	1.398	-0.158
O	-0.968	0.433	-0.646
C	-1.441	0.045	-1.949
H	-1.292	0.880	-2.637
H	-2.513	-0.163	-1.865
C	-0.646	-1.205	-2.324
H	0.258	-0.936	-2.877
H	-1.233	-1.898	-2.930
C	-0.276	-1.776	-0.954
H	0.601	-2.424	-0.968
H	-1.117	-2.324	-0.517
O	-0.962	1.709	1.777
H	-1.139	1.670	0.822
Cl	0.651	-1.932	2.105

Supplementary References

1 Nakao, Y., Fujita, M., Warabi, K., Matsunaga, S. & Fusetani, N. Miraziridine A, a Novel Cysteine Protease Inhibitor from the Marine Sponge *Theonella* aff. *mirabilis*[1]. *Journal of the American Chemical Society* **122**, 10462-10463 (2000). https://doi.org/10.1021/ja001859j

2 Vervoort, H., Fenical, W. & Epifanio, R. D. A. Tamandarins A and B: New Cytotoxic Depsipeptides from a Brazilian Ascidian of the Family Didemnidae. *The Journal of Organic Chemistry* **65**, 782-792 (2000). https://doi.org/10.1021/jo991425a

3 Umezawa, H. *et al.* Pepstatin, a new pepsin inhibitor produced by actinomycetes. *J Antibiot* **23**, 259-262 (1970).

4 Williamson, R. T., Buevich, A. V., Martin, G. E. & Parella, T. LR-HSQMBC: A Sensitive NMR Technique To Probe Very Long-Range Heteronuclear Coupling Pathways. *The Journal of Organic Chemistry* **79**, 3887-3894 (2014). https://doi.org/10.1021/jo500333u

5 Haasnoot, C. A. G., de Leeuw, F. A. A. M. & Altona, C. The relationship between proton-proton NMR coupling constants and substituent electronegativities—I: An empirical generalization of the karplus equation. *Tetrahedron* **36**, 2783-2792 (1980). https://doi.org/https://doi.org/10.1016/0040-4020(80)80155-4

6 Yasumoto, T. *et al.* Diarrhetic shellfish toxins. *Tetrahedron* **41**, 1019-1025 (1985). https://doi.org/10.1016/s0040-4020(01)96469-5

7 Juaristi, E. & Cuevas, G. Recent studies of the anomeric effect. *Tetrahedron* **48**, 5019-5087 (1992). https://doi.org/https://doi.org/10.1016/S0040-4020(01)90118-8

8 Suzuki, T., Beuzenberg, V., Mackenzie, L. & Quilliam, M. A. Liquid chromatography–mass spectrometry of spiroketal stereoisomers of pectenotoxins and the analysis of novel pectenotoxin isomers in the toxic dinoflagellate Dinophysis acuta from New Zealand. *Journal of Chromatography A* **992**, 141-150 (2003). https://doi.org/https://doi.org/10.1016/S0021-9673(03)00324-8
